# CPAG: software for leveraging pleiotropy in GWAS to reveal similarity between human traits links plasma fatty acids and intestinal inflammation

**DOI:** 10.1186/s13059-015-0722-1

**Published:** 2015-09-15

**Authors:** Liuyang Wang, Stefan H. Oehlers, Scott T. Espenschied, John F. Rawls, David M. Tobin, Dennis C. Ko

**Affiliations:** Department of Molecular Genetics and Microbiology, School of Medicine, Duke University, Durham, NC 27710 USA; Department of Medicine and the Center for Human Genome Variation, School of Medicine, Duke University, Durham, NC 27710 USA

## Abstract

Meta-analyses of genome-wide association studies (GWAS) have demonstrated that the same genetic variants can be associated with multiple diseases and other complex traits. We present software called CPAG (Cross-Phenotype Analysis of GWAS) to look for similarities between 700 traits, build trees with informative clusters, and highlight underlying pathways. Clusters are consistent with pre-defined groups and literature-based validation but also reveal novel connections. We report similarity between plasma palmitoleic acid and Crohn's disease and find that specific fatty acids exacerbate enterocolitis in zebrafish. CPAG will become increasingly powerful as more genetic variants are uncovered, leading to a deeper understanding of complex traits. CPAG is freely available at www.sourceforge.net/projects/CPAG/.

**Electronic supplementary material:**

The online version of this article (doi:10.1186/s13059-015-0722-1) contains supplementary material, which is available to authorized users.

## Background

During the past decade, genome-wide association studies (GWAS) have identified thousands of genetic variants associated with human traits and diseases. As of 4 September 2013, the National Human Genome Research Institute (NHGRI) Catalog of Published GWAS had manually curated more than 11,000 single nucleotide polymorphisms (SNPs) associated with over 700 traits from more than 1400 studies [1]. These studies have revealed important insights regarding how common variants can affect individual diseases and traits [2]. However, additional insights can be gained when the results of multiple GWAS or even all published GWAS are integrated together.

One striking finding from comparative analyses of GWAS is that pleiotropic SNPs are quite abundant across the human genome. Pleiotropy occurs when a genetic locus affects multiple different phenotypes, for example, by encoding a protein with multiple activities, having different roles in different cells, or by influencing multiple pathways. About 5 % of SNPs and 17 % of genes implicated in GWAS have been associated with multiple traits [3]. Some of these genes exhibit pleiotropy in the strict sense of affecting multiple seemingly unrelated phenotypes, while other SNPs and genes can perhaps be more correctly designated as participating in “cross-phenotype” associations [4]. Cross-phenotype associations may reflect pleiotropy or varying outcomes of a single biological activity in the context of different cell/tissue types and environmental triggers. Other cross-phenotype associations may reflect associations with phenotypes of different scales, such as the same SNPs affecting plasma metabolite concentrations and also disease risk. Cross-phenotype associations have particularly been noted in autoimmunity [5, 6]. For example, the *PTPN22* gene has been associated with rheumatoid arthritis [7], Crohn’s disease [8], systemic lupus erythematosus [9] and type 1 diabetes [10]. Cross-phenotype association analysis leveraging pleiotropy and similarity of traits can provide opportunities for understanding the shared genetic underpinnings among associated traits and diseases, revealing new insights into the pathophysiology of disease.

Previous studies have developed approaches to identify and characterize cross-phenotype associations (reviewed in [4]). These approaches fall broadly into multivariate frameworks that jointly analyze SNPs for multiple phenotypes and meta-analyses of traditional univariate SNP analyses. The prior category includes polygenic scoring and linear mixed-effect models that can assess the degree of pleiotropy between two phenotypes but do not hone in on specific variants. The multivariate approaches also include testing the association of SNPs with multiple phenotypes using a unified framework. However, multivariate approaches generally can only be applied when the same individuals have been scored for multiple phenotypes. In contrast, univariate approaches can be applied post hoc to GWAS that have already been conducted on different populations. Previous studies using this approach were valuable at pointing out the high amount of apparent pleiotropy in human SNPs [3], the enrichment of certain SNP classes in pleiotropic SNPs [3], and characterizing the degree of similarity using the Jaccard similarity index [11]. Very recently, Li et al. [12] calculated cosine similarity indices between traits and diseases in a private GWAS database, restricted to only genic SNPs, and validated cross-phenotype SNPs with electronic medical record mining. While these recent studies underscore the high level of interest in cross-phenotype associations, much work remains to be done. A systematic comparison of similarity indices for cross-phenotype analysis has not been carried out. Furthermore, most approaches to date have relied on networks for visualization, which can be difficult to interpret on such large datasets. Importantly, none of the existing methods allow for new, user-defined groups of SNPs or genes to be used to easily interrogate the interaction network. Finally, methods to study cross-phenotype associations have not been coupled to experimental methods to quickly test hypotheses.

In this study, we have developed and validated an integrated framework for cross-phenotype analysis of GWAS, CPAG. In addition to confirming overlap between known related diseases, our method revealed unexpected evidence of shared genetic architecture among previously seemingly disparate traits. Specifically, intrigued by the shared associations between GWAS of plasma levels of a fatty acid and Crohn’s disease, we tested the hypothesis that fatty acids could exacerbate intestinal inflammation using a zebrafish model. We have implemented CPAG in a user-friendly program that accepts user-defined lists of SNPs, allowing for easy visualization and interpretation of any genome-wide result in the context of all published GWAS.

## Results

### Cross-phenotype and pleiotropic SNPs are enriched in the NHGRI GWAS Catalog

**Fig. 1:**
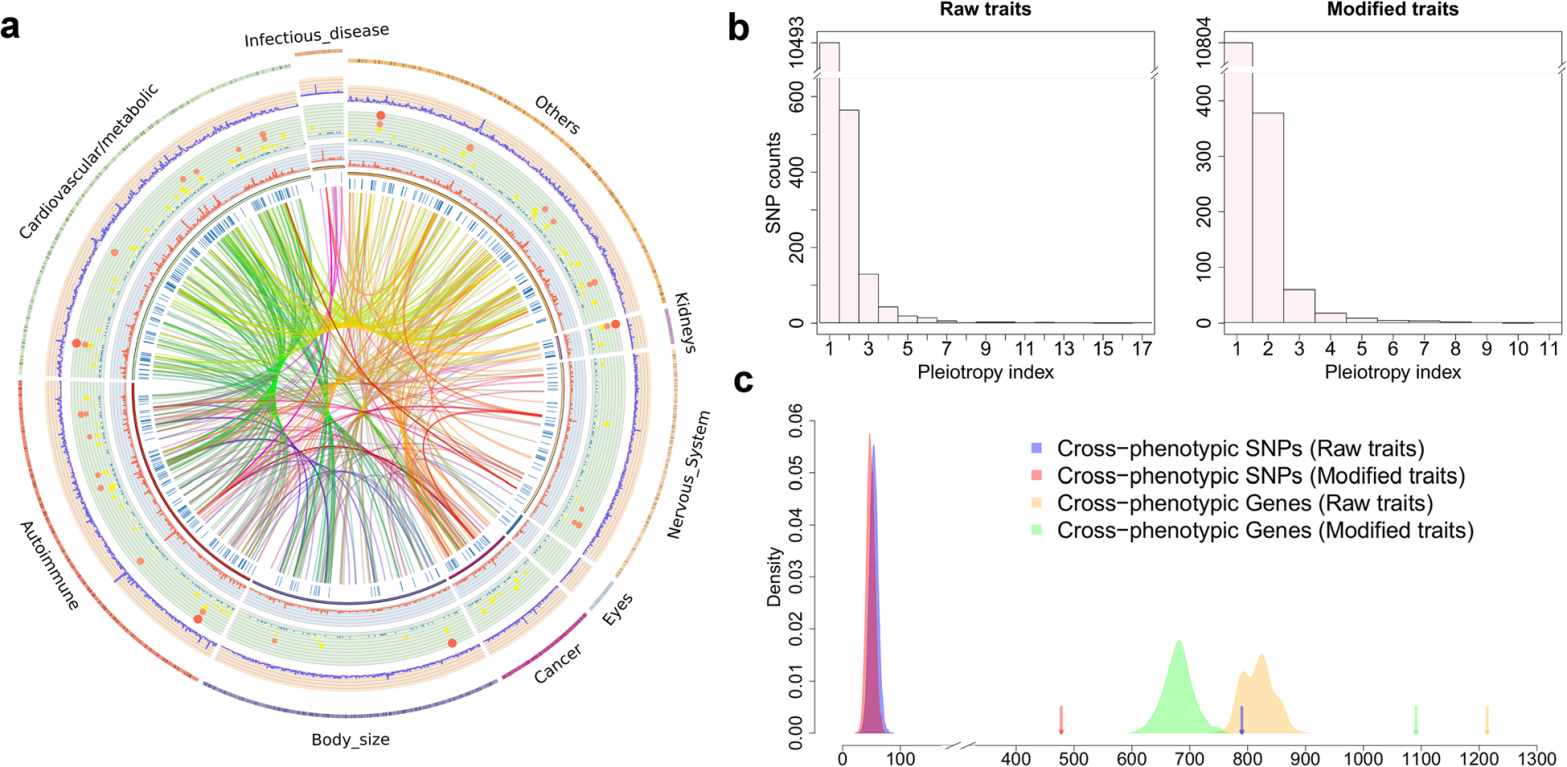
Cross-phenotype and pleiotropic SNPs in the NHGRI GWAS Catalog. **a** Circle plot of all NHGRI GWAS Catalog SNPs grouped into nine broad categories of traits. Moving from the outermost circle inward, the circles represent a linear representation of the karyotype of the human genome with different background colors for each trait category, density of GWAS SNPs per 5 Mb along the genome, scatter plot of “pleiotropic index” (the number of traits associated with each SNP), and density plot of cross-phenotype SNPs in 5-Mb windows along the genome. In the scatter plot of “pleiotropic index” larger circles and colors from *blue* to *yellow* to *orange* to *red* represent a SNP being associated with more traits. The *blue vertical lines* represent genomic positions of cross-phenotype SNPs within each pre-defined group and *inner rainbow lines* represent cross-phenotype SNPs connecting different groups, with the color indicative of the trait categories being connected. **b** Histogram of NHGRI GWAS Catalog SNPs based on “pleiotropic index”. Raw traits are directly from the NHGRI GWAS Catalog while modified traits have been manually curated to merge closely related phenotypes and to remove phenotype categories that had combined multiple diseases. A total of 789 SNPs 7.0 %) were associated with more than one trait for raw traits and 478 SNPs (4.2 %) were associated with more than one trait for modified traits. **c** Enrichment of cross-phenotype SNPs in the NHGRI GWAS Catalog. Distribution of cross-phenotype SNPs expected by chance (histograms) are plotted along with the observed number of cross-phenotype SNPs in the NHGRI GWAS Catalog (*arrows*). We used a permutation-based method to test whether there is significant enrichment of cross-phenotype SNPs/genes in the GWAS Catalog. We randomly resampled SNPs from a pool of unique SNPs and assigned to each disease and constructed the null distribution by repeating this process 10,000 times. Significant differences (*p* < 0.0001) were detected for both SNPs and genes

Before determining the degree of similarity among all human traits and diseases in the NHGRI GWAS Catalog, we assessed whether the degree of cross-phenotype associations was sufficient to warrant such an approach. We carried out a systematic analysis of all SNPs in the NHGRI GWAS Catalog and found that cross-phenotype SNPs are much greater than expected by chance. A total of 789 (7.0 %) SNPs are associated with more than one human trait. All SNPs in the GWAS Catalog can be depicted using a circle plot with lines connecting SNPs associated with multiple traits (Fig. 1a). We classified traits into nine broad categories, and the circle plot demonstrates that 40 % of cross-phenotype SNPs (2.8 % of all SNPs) connect traits in different categories. While most cross-phenotype SNPs are only associated with two traits (Fig. 1b), the SNP showing the most associations, rs1260326 (gene *GCKR*), is associated with 17 human traits (Additional file 1).

A permutation test demonstrated there was a highly significant enrichment of cross-phenotype SNPs in the NHGRI GWAS Catalog. We resampled SNPs from a pool of unique SNPs (HapMap phase 3 [13]) to randomly assign SNPs to each trait in the NHGRI GWAS Catalog and determined the fraction of cross-phenotype SNPs. The null distribution for cross-phenotype SNPs was constructed by repeating this process 10,000 times. For most permutations (95 %), the number of cross-phenotype SNPs fell between 40 and 69, and the greatest number of pleotropic SNPs reached in a single permutation was 86. Remarkably, the actual observed number of cross-phenotype SNPs in the GWAS Catalog is almost ten times more at 789 (Fig. 1c; *p* < 0.0001). Some traits within the NHGRI GWAS Catalog are clearly closely related (such as total cholesterol levels and low-density lipoprotein cholesterol levels), so the number of cross-phenotype SNPs is inflated compared with SNPs displaying pleiotropy in the strict sense. To reduce this inflation, closely related phenotypes were merged and phenotypes in the NHGRI GWAS Catalog that combined multiple diseases were removed, reducing the number of traits from 786 to 461 (termed “modified traits”). Even with this merging of related traits, there was still a substantial enrichment of pleiotropic SNPs (478 SNPs; Fig. 1c; *p* < 0.0001). Finally, we also performed a gene-based permutation test (restricted only to SNPs within genes as classified by the NHGRI GWAS Catalog) and a similar enrichment for pleiotropy was observed (1214 genes for raw and 1091 for modified traits; *p* < 0.0001 for raw or modified traits). These results demonstrate a clear enrichment of cross-phenotype SNPs in human traits and diseases.

### Cross-phenotype SNPs allow for identification of clusters of human traits

By employing the extensive cross-phenotype associations among SNPs associated with human traits, we developed methods to identify traits that are associated with the same genetic variants and to cluster traits to visualize this information.

First, we compared three methods for calculating SNP overlap between pairs of traits: 1) a SNP-based method that counts only exact SNP matches; 2) a SNP-based method corrected for linkage disequilibrium (LD) where SNPs with *r*^2^ > 0.6 for two or more traits are considered overlapping (called the SNP_LD method here); and 3) a gene-based method. The SNP-based method is the most conservative, because it requires that the exact same SNP be reported in the NHGRI GWAS Catalog for two different traits. The SNP-based method corrected for LD determines if SNPs that are in high LD (*r*^2^ > 0.6) were identified by different studies and includes these as overlapping SNPs in the similarity index. Neither SNP method makes any assumption about the gene being affected — this is an advantage as 1) any gene-based method is only as good as the prediction of which gene is being affected by the causal SNP and 2) about 45 % of SNPs reported in the NHGRI GWAS Catalog fall into intergenic regions based on NHGRI annotation [1]. However, assignment to genes does allow for further downstream analysis, such as gene-set enrichment analysis (GSEA; see below). In the gene-based similarity analysis, we used the mapped gene assignment from the NHGRI GWAS Catalog — SNPs within genes are assigned to the genes they are located in while intergenic SNPs are assigned to the genes on both sides of the intergenic region. Formally evaluating these three different approaches (SNPs, SNP_LD, and gene-based methods) revealed that the SNP_LD method identified the greatest fraction of overlapping trait pairs with significant similarity (*p* < 0.05 after Bonferroni correction) (Figure S1a in Additional file 2). Furthermore, examining the same trait pairs revealed that the *p* values from the SNP_LD method were in general lower than the other two methods (Figure S1b in Additional file 2). Therefore, the SNP_LD method reveals the largest fraction of trait pairs with significant similarity and is the most robust of the three methods. SNP_LD was used for the remainder of analyses in this manuscript.

**Fig. 2:**
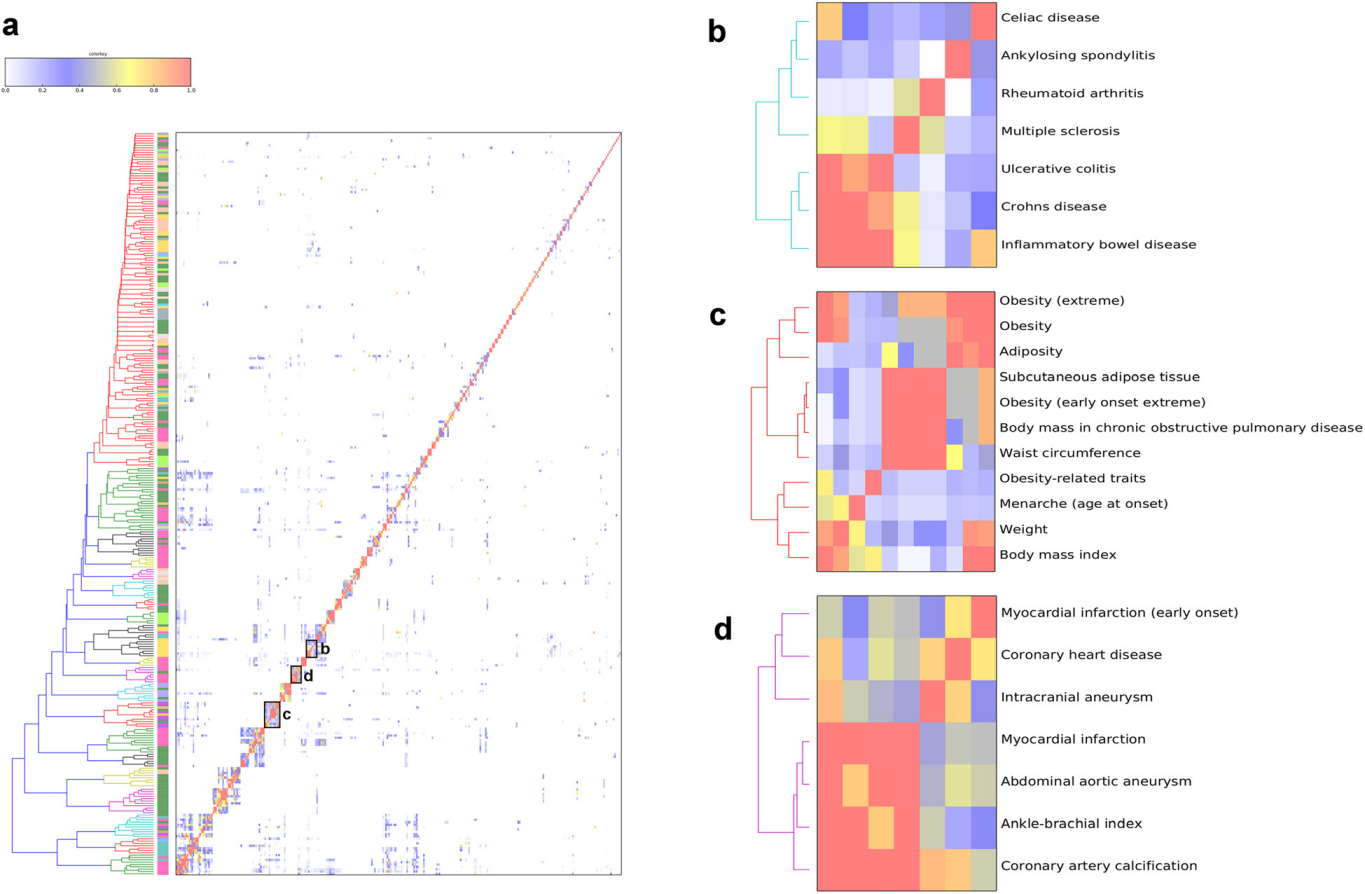
Hierarchical clustering of NHGRI human traits. **a** Dendrogram and heat map of similarity for pairwise human traits. The hierarchical dendrogram was constructed based on the Chao–Sorensen similarity index, and significance of similarity was measured using a hypergeometric test implemented in the CPAG program. A total of 317 traits having at least one significant association (*p* < 0.05) against other traits were included. Colors in the heat map are based on the Chao–Sorensen index and scaled according to the color key. Colored blocks along the y-axis of the heat map are indicative of the nine assigned categories of traits. Enlarged versions of an autoimmune cluster (**b**), obesity cluster (**c**) and atherosclerosis cluster (**d**) are shown. Additional file 4 contains a higher resolution dendrogram and heat map with all 317 traits labeled

Next, a similarity index was required to quantify the magnitude of overlap for all trait pairs and allow for generation of similarity matrices, heat maps, and clustering. As different similarity indices can have profound consequences in assessing and visualizing similarity [14], we compared several similarity indices (Jaccard, Sorensen, Chao–Jaccard, Chao–Sorensen, Morisita, Morisita–Horn, Pearson correlation coefficient, cosine, Simpson, geometric, and connection specificity index (CSI)). The similarity index for a pair of traits was used as a distance measure for constructing a heat map and tree based on hierarchical clustering. The significance of overlap was assessed using Fisher’s exact test, as well as with empirical *p* values based on permutation, and only traits with statistically significant similarity after multiple-test correction were used in clustering. This analysis was performed on all SNPs with reported *p* < 1 × 10^−7^ in the NHGRI GWAS Catalog (Fig. 2a; Additional files 3 and 4). This threshold was chosen as it excluded possible false positive SNPs with less significant *p* values in the catalog but did not result in a reduction of significant trait pairs as observed when the *p* value threshold decreased beyond *p* < 1 × 10^−7^ (Additional file 5). The result is a searchable table and a GWAS similarity tree of 341 human traits based solely on shared genetic architecture.

Clusters identified by CPAG are broadly in agreement with known biology. Clusters of cholesterol-related traits, type 2 diabetes, pigmentation, hematological traits, obesity, kidney function, atherosclerosis, cell adhesion, and autoimmunity are readily discernable. Within larger clusters, known relationships are also observed. In an autoimmune cluster (Fig. 2b), Crohn’s disease and ulcerative colitis, known subtypes of inflammatory bowel disease, are tightly clustered (18 shared LD-corrected SNPs; Bonferroni-corrected *p* < 1.2 × 10^−49^). An obesity cluster (Fig. 2c) is notable for not only including several different measures of adiposity but also “Menarche (age of onset)”. Obesity is well known to be associated with early menarche [15]. An atherosclerosis cluster (Fig. 2d) contains both measures of atherosclerosis severity (coronary artery calcification, coronary heart disease, ankle-brachial index) as well as acute consequences of atherosclerosis (myocardial infarction, abdominal aortic aneurysm, and intracranial aneurysm). Similar clusters were seen when we adjusted the *p* value threshold and consequently the number of included SNPs was altered (Additional file 5), but not surprisingly, the number of traits in clusters decreased when the *p* value threshold was more stringent (Additional file 6). We conclude that hierarchical clustering based on similarity indices resulted in informative groupings that agreed with prior knowledge.

However, the clustering results varied substantially based on which similarity index was used (Additional files 7, 8, 9, 10, 11, 12, 13, 14, 15, and 16). Therefore, we conducted statistical comparisons of the similarity indices and the trees generated using them.

### Comparison of CPAG clusters generated by different similarity indices

We evaluated 11 different similarity indices for their performance in CPAG. The best methods should have 1) minimum heterogeneity of clusters based on their predefined classification, as we expect diseases from the same group to cluster together, and 2) maximum size of clusters, as a method that has very small clusters would not provide as much insight. We defined heterogeneity as discordance of observed disease groups with predefined disease groups, and applied entropy methods to compute the heterogeneity of the tree.

**Fig. 3:**
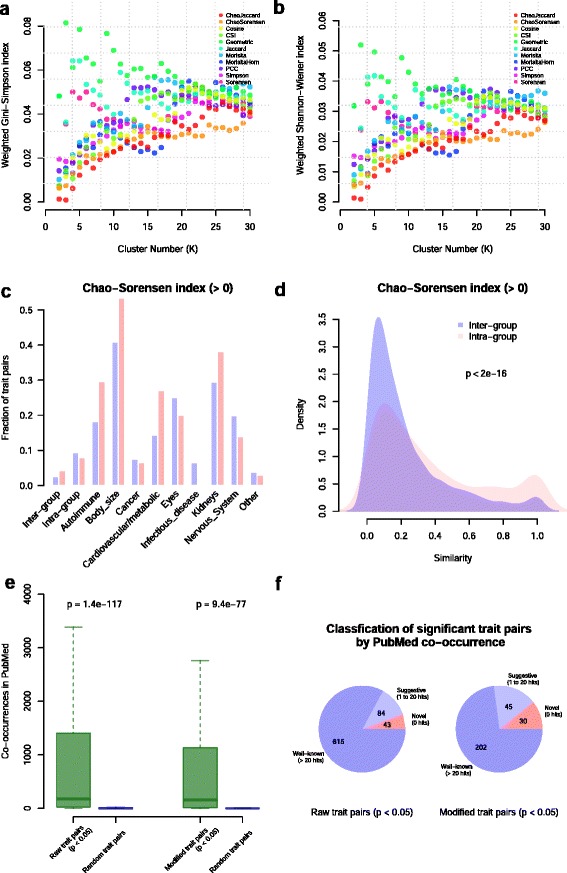
Entropy-based comparisons of similarity indices and computational validation of CPAG clusters. **a** Weighted heterogeneity versus cluster number using the Gini–Simpson index. We calculated weighted heterogeneity using equation ${\mathrm{H}}_{\mathrm{e}}^{\hbox{'}}=\frac{1}{\mathrm{K}}{\displaystyle \sum}\frac{{\mathrm{H}}_{\mathrm{e}}}{{\mathrm{N}}_{\mathrm{i}}}$ , which can be interpreted as average heterogeneity per cluster per disease. The weighted heterogeneity captures variation of both cluster size and heterogeneity. **b** Weighted heterogeneity versus cluster number using Shannon–Wiener entropy index. Either entropy index indicates the Chao–Sorenson index results in the largest and least heterogeneous clusters based on the nine pre-defined trait categories. **c** The fraction of trait pairs with similarity > 0 for raw traits (*blue*) and modified traits (*pink*) is greater within pre-defined categories (*Intra-group*) than between categories (*Inter-group*). The fractions vary across different trait groups, indicating greater similarity among some groups of traits compared with others. **d** Distribution of non-zero similarity values for inter-group and intra-group for raw traits shows greater similarity for comparisons within pre-defined groups. The *p* value was calculated using Kolmogorov-Smirnov test. **e** Published literature supports the association of pairwise traits identified by CPAG. We searched PubMed using each trait pair and recorded the number of co-occurrences in titles and abstracts. The box plots represent the distribution of co-occurrences for raw or modified significant trait pairs compared with the co-occurrence distributions of 10,000 random trait pairs. We found significantly lower co-occurrences for both raw and modified traits based on the Mann–Whitney rank sum test. **f** CPAG reveals both well-established and novel trait pairs. The pie charts represent the fractions of trait pairs for three different categories: novel trait pairs with no co-occurrences in PubMed, suggestive trait pairs with co-occurrences between 1 and 20, and well-known trait pairs with >20 co-occurrences. The number of trait pairs within each category is given within each pie segment. Lists of potentially novel trait pairs are provided in Additional files 19 and 20. *PCC* Pearson correlation coefficient

We found that the method that produced the tree with the lowest weighted heterogeneity (heterogeneity/median cluster size) was Chao–Sorensen (Fig. 3a, b; Additional file 17). This was especially apparent at a higher number of clusters (K > 18). With very low cluster number, Chao–Jaccard had the lowest weighted heterogeneity, but identifying such few clusters from such a large tree and heat map has limited utility. The Pearson correlation coefficient gave the most heterogeneous clusters by objectively using the entropy methods, and separated traits expected to cluster together (such as Crohn’s disease and ulcerative colitis). Other similarity indices, including those implemented in other methods for assessing SNP and gene similarity such as CSI [16] and cosine [12], tended to exhibit higher weighted heterogeneity.

The Chao–Sorensen and Chao–Jaccard similarity indices, which are commonly used in ecology research for studying community species diversity, use a probabilistic model to modify the traditional Sorensen and Jaccard indices [17]. The modified estimators are less biased to sample size and incorporate the effects of unobserved shared members and replicated associations, and published simulations indicate they outperform other methods [18, 19]. Our results support this, and therefore we used Chao–Sorensen for the remainder of our analysis in quantifying the strength of similarity and performing clustering analysis using the SNP_LD algorithm, while statistical significance for SNP_LD similarity was evaluated using Fisher’s exact test and a permutation-based test. To our knowledge, this is the first application of the Chao–Sorensen similarity index to studying human genetics.

### Computational validation shows CPAG agrees with previously known disease relationships but also reveals novel connections

To assess the validity and value of categorizing traits by CPAG, we used both computational and experimental validation. We determined how similarity index values differed within predetermined trait categories versus between trait categories. The prediction is that if CPAG categorization is indicative of shared biology, then similarity indices will be greater within groups compared with between groups. Indeed, the fraction of traits with similarity > 0 (i.e., those traits that share SNPs with other traits) was higher for intra-group trait pairs compared with inter-group trait pairs (Fig. 3c). Furthermore, for trait pairs that do have similarity > 0, the distribution of similarity values is skewed towards higher values for intra-group trait pairs compared with inter-group trait pairs (Fig. 3d; Additional file 18; *p* < 2.2 × 10^−16^ and *p* < 2.05 × 10^−9^ for raw traits and modified traits, respectively). Therefore, both the fraction of trait pairs showing overlap as well as the amount of similarity for these pairs is greater within predefined disease groups. Thus, categorization of traits by CPAG is well in agreement with trait categorization based on medical knowledge.

Trait pairs which have statistically significant similarity (*p* < 0.05 after Bonferroni correction) are more often mentioned together in PubMed abstracts than random trait pairs (Fig. 3e). This was true with both raw (*p* = 1.4 × 10^−117^) and modified trait pairs (*p* = 9.4 × 10^−77^). Of the 277 significant modified trait pairs, 202 (73 %) had >20 co-occurrences in PubMed, indicating that there is likely well-known similarity between these pairs of traits (Fig. 3f). While being mentioned together in an abstract does not necessarily mean the two traits are truly related, the large number of trait pairs that have a high number of co-occurrences in abstracts certainly supports the contention that trait pairs detected by CPAG are biologically relevant. However, many traits pairs with statistically significant similarity were not found to co-occur in PubMed. Thirty of the significant modified trait pairs (11 %) had no co-occurrences in PubMed, indicating potentially novel associations (Fig. 3f; Additional files 19 and 20). Thus, while there are significantly more co-occurrences than would be expected based on chance, there are still many potentially novel associations that should undergo further testing to determine their biological and clinical relevance.

### Similarity can occur for multiple reasons and is supported by published evidence

While the density distributions of intra-group and inter-group comparisons and the co-occurrence in literature data suggests that clustering based on shared GWAS SNPs is recapitulating known disease categorization, the value of finding connections between traits and diseases is exemplified by examination of individual overlapping trait pairs. Cross-phenotype associations can occur for multiple reasons but we broadly classify them into four categories.

#### Category 1

**Fig. 4:**
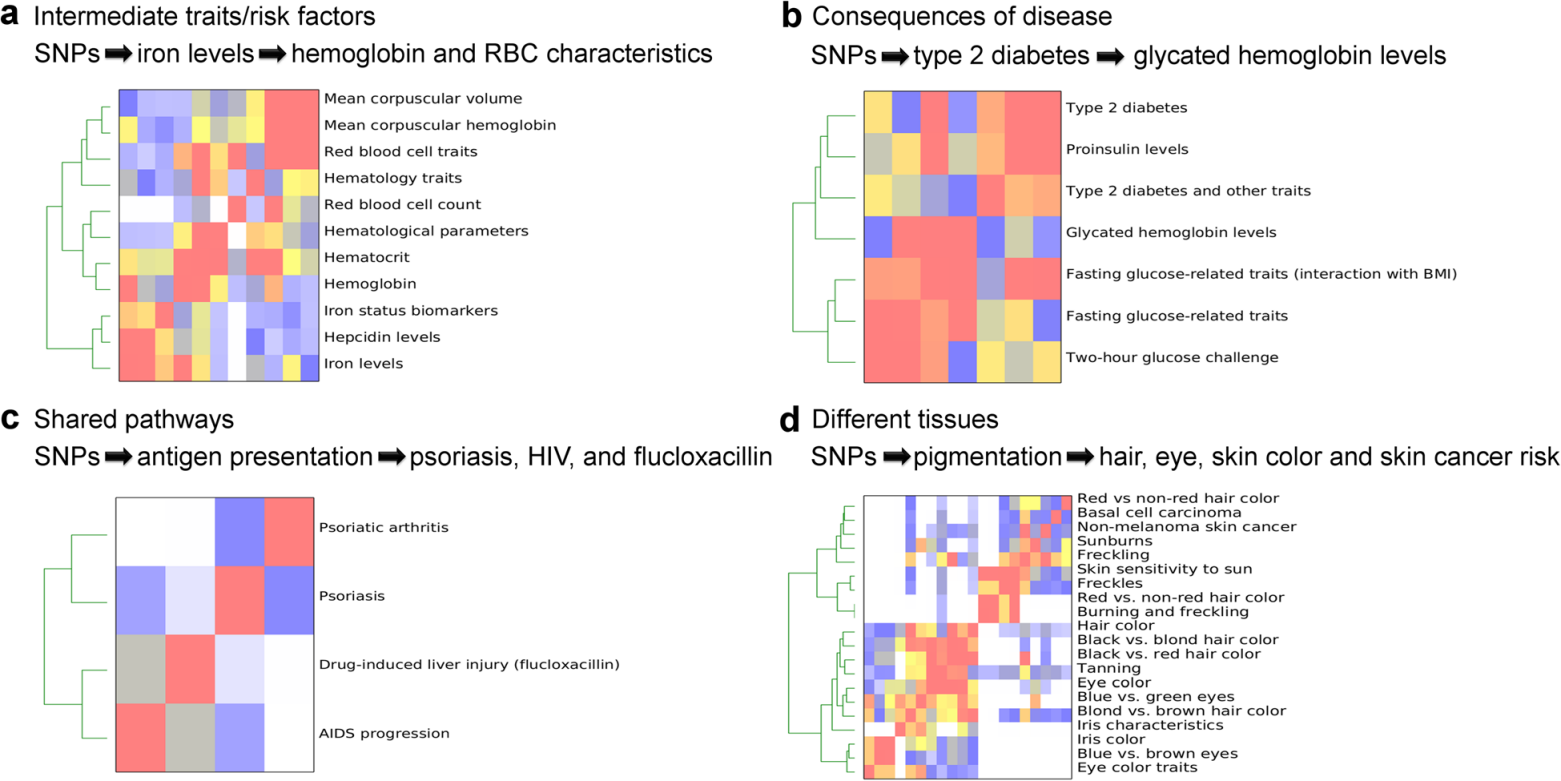
CPAG identifies clusters attributable to four broad models of trait similarity. **a** Similarity due to an intermediate trait being a risk factor for a disease or other more complex trait, as exemplified by iron levels affecting hemoglobin and red blood cell (RBC) traits. **b** Similarity due to a trait being a consequence of a disease, as exemplified by glycated hemoglobin (hemoglobin A1C) being caused by increased plasma glucose and type 2 diabetes. **c** Similarity due to a single pathway being associated with two or more diseases, as exemplified by possibly antigen presentation affecting both psoriasis and AIDS progression. **d** Similarity due to the same genes having effects in different tissues or on different signaling pathways, as exemplified by similarity in pigmentation traits in skin, eyes, and hair

Cross-phenotype associations can be due to SNP similarity between an intermediate trait/risk factor and disease. An intermediate trait (such as plasma levels of a metabolite) can be a risk factor for a disease. For example, the iron-related traits are clustered with anemia and red blood cell traits because genetic variants alter iron levels which then subsequently affect hemoglobin and red blood cell production (Fig. 4a).

#### Category 2

Cross-phenotype associations can be due to SNP similarity between a disease and a consequence of disease. This is the reverse scenario for the first class, where a trait is actually a result of the disease. For example, increased glycated hemoglobin (hemoglobin A1C) levels are a consequence of type 2 diabetes and high plasma glucose concentrations. Because of this, it is a commonly used clinical marker for monitoring plasma glucose control in patients [20]. Thus, it is not surprising that glycated hemoglobin clusters with type 2 diabetes, proinsulin levels, and fasting plasma glucose (Fig. 4b).

#### Category 3

Cross-phenotype associations can be due to SNP similarity between two traits affected by the same gene/pathway. This occurs when a SNP affects a gene (by altering the protein coding sequence or expression level, for example) that acts in a single pathway but that is manifest in two or more diseases. For example, we observed significant overlap between SNPs associated with psoriasis and AIDS progression (Fig. 4c; *p* = 6.81 × 10^−6^ after Bonferroni correction). This connection is driven by two different variants in the HLA region (*r*^2^ = 0.43 in CEU HapMap phase 3 population), and others have noted several additional variants in the same region that predispose to psoriasis and protect against HIV [21]. One plausible explanation is that these genetic variants regulate antigen presentation which then impacts two diseases in different ways — in one case controlling viral infection and in the other case regulating autoimmunity.

#### Category 4

Cross-phenotype associations can be due to SNP similarity between two traits affected by the same gene having effects in different tissues or on different pathways. For example, there is a large cluster of traits for hair color, eye color, skin color, tanning, and skin cancer (Fig. 4d). This cluster is driven by overlapping SNPs affecting genes involved in pigmentation, such as the melanocortin 1 receptor, a G-protein coupled receptor that stimulates melanin production in hair, eyes, and skin. While this example involves the same gene having effects in different tissues, other cross-phenotype associations may involve the same gene having effects on different pathways. For example, rs4420638 is a SNP in the apolipoprotein gene cluster on chromosome 19 that is associated with 13 traits in the NHGRI GWAS Catalog. While many of these traits are related lipid traits (including total cholesterol, low-density lipoprotein cholesterol, high-density lipoprotein cholesterol, and triglycerides), others include longevity, age-related macular degeneration, and Alzheimer’s disease. Apolipoproteins are key components of lipoproteins that mediate lipid trafficking and uptake. This role can explain their association with lipid traits, but their association with other traits may involve their roles in other pathways, such as neuronal survival, inflammatory signaling, and amyloid binding [22].

### GSEA of shared SNPs elucidates pathways responsible for the trait similarity

While the causal relationship among traits with GWAS overlap may be obvious, for many connections, the reason for the overlap may be unknown. To provide insight as to what is driving the similarity, CPAG provides lists of overlapping SNPs and genes for each pair of traits. Furthermore, overlapping genes are automatically examined by GSEA using the Molecular Signatures Database “curated gene set” (set C2) to reveal possible pathways that may be shared between the two traits. We relied on Fisher’s exact test based on SNP_LD to identify trait pairs that were significantly similar, but used the mapped genes (based on NHGRI GWAS Catalog) to provide the overlapping genes to query the C2 dataset and reveal if particular pathways were driving the similarity. For example, the traits of “D-dimer levels” and “venous thromboembolism” have significant overlap (*p* = 4.9 × 10^−7^ after Bonferroni correction) and GSEA reveals that this is being driven by coagulation pathways (Biocarta_extrinsic pathway, *p* = 7.0 × 10^−7^). The SNPs that cause the enrichment in coagulation pathways implicate the factor V coagulation factor and fibrinogen alpha and gamma chains. D-dimers are fibrin degradation products from clots that are used as a marker for active coagulation. The overlap between these two traits indicates that genetic variants that affect fibrinogen and clot formation alter risk of venous thromboembolism, which is reflected in altered D-dimer levels.

A second example involves the overlap between Crohn’s disease and psoriasis. While both diseases have been the subject of multiple large GWAS, they only overlap with two SNPs (*p* = 0.33 after Bonferroni correction). However, the genes implicated by the two SNPs are both in the interleukin (IL)-23 pathway (*IL23R* and *TYK2*; *p* = 0.0001). The analysis suggests that IL-23 signaling is important for risk of both Crohn’s disease and psoriasis. In fact, ustekinumab is a monoclonal antibody against IL-23 that is approved for use against psoriasis [23, 24] but has also shown promise in treating Crohn’s disease [25, 26]. Thus, CPAG not only suggests that the etiology of Crohn’s disease and psoriasis may share some genetic underpinnings, but highlights that the mechanism likely involves the IL-23 pathway.

### Testing a CPAG-generated hypothesis in zebrafish reveals plasma fatty acids worsen intestinal inflammation

Possibly the greatest utility from CPAG comes from overlap where two traits not known to be closely related demonstrate similar genetic associations. Such similarity may be most useful when it occurs between a molecular trait and a disease trait because modulation of the molecular trait may affect the risk or severity of the disease. One of the 43 novel connections revealed by CPAG and the PubMed co-occurrence analysis (Fig. 3f; Additional file 19) was GWAS overlap of plasma palmitoleic acid levels with Crohn’s disease (*p* = 0.0006 after Bonferroni correction). Previous GWAS for plasma levels of four specific fatty acids [palmitic acid (16:0), stearic acid (18:0), palmitoleic acid (16:1n-7), and oleic acid (18:1n-9)] identified five SNPs associated with palmitoleic acid levels [27]. Intriguingly, two of these are also among the 163 SNPs associated with risk of Crohn’s disease (rs102275 near *FADS1* and rs780093 near *GCKR*; overlap of 0.0006 expected by chance for >3000-fold enrichment), and the directions of effect indicate high palmitoleic acid could be associated with increased disease risk. The incidence of Crohn’s disease is higher in countries with a high fat diet, suggestive that fat intake and lipid metabolism might play an important role in Crohn’s disease risk [28]. Furthermore, dietary questionnaire studies indicate that high fat intake, including monounsaturated fatty acids, is associated with increased Crohn’s disease risk [29, 30]. Metabolomic measurements have also revealed that several plasma fatty acids trend towards being elevated in Crohn’s disease patients [31, 32]. Therefore, dietary studies and correlations from metabolomics are suggestive of a link between fatty acids and Crohn’s disease risk, but no human genetic susceptibility loci underlying and connecting the two had been previously reported. Elevated plasma fatty acids might be a factor that increases risk of Crohn’s disease, or a consequence of altered lipid absorption/metabolism due to gut inflammation, or a further downstream consequence of the complex pathophysiology of Crohn’s disease.

**Fig. 5:**
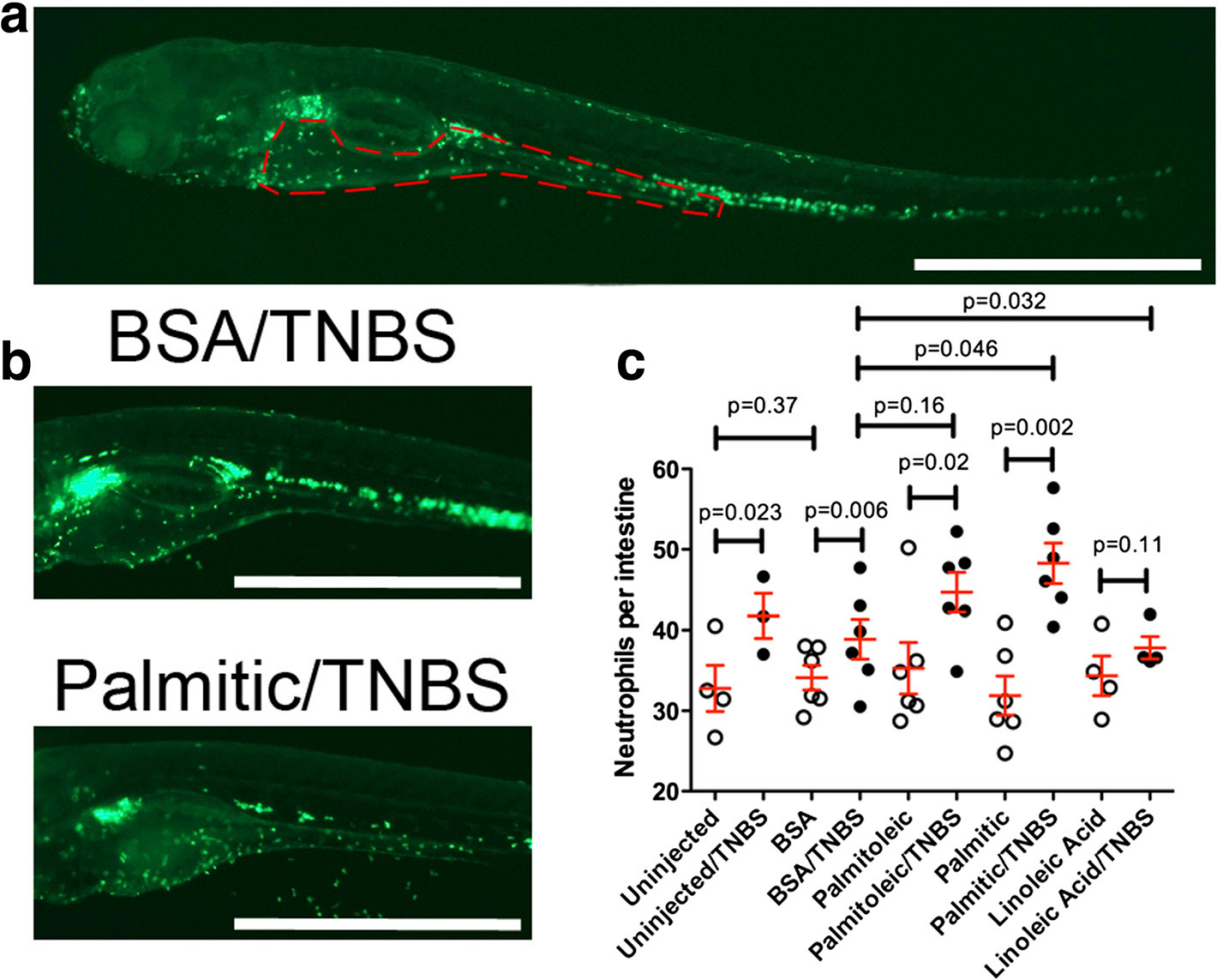
Exogenous serum fatty acid exacerbates colitis in zebrafish larvae. **a** *Tg(lyzC:EGFP)* ^nz117^ larvae with *red outline* demarcating the edge of the intestine. Enhanced green fluorescent protein in these fish, under the control of the lysozyme C promoter, mark mature neutrophils, which are a marker of inflammation. **b** Representative images of *Tg(lyzC:EGFP)* ^nz117^ larvae. Scale bar indicates 1 mm. **c** Quantification of intestinal neutrophils in 6 days post-fertilization (dpf ) zebrafish larvae exposed to 25–30 μg/ml TNBS from 3 dpf. Bar graphs are the mean ± standard error of the mean of six independent experiments (except uninjected and linoleic acid were from four independent experiments) with an average of 14.3 larvae evaluated in each group in each experiment. *P* values are from paired t-tests using the means of each group from each experiment. The number of total larvae evaluated in each group was: *Uninjected*, 48; *Uninjected/TNBS*, 45; *BSA*, 75; *BSA/TNBS*, 61; *Palmitic acid*, 91; *Palmitic/TNBS*, 93; *Palmitoleic acid*, 83; *Palmitoleic acid/TNBS*, 98; *Linoleic acid*, 50; *Linoleic acid/TNBS*, 70

To test whether increased plasma fatty acid was sufficient to exacerbate intestinal inflammation, we utilized a zebrafish model. Trinitrobenzene sulfonic acid (TNBS) is a commonly used chemical injury method to induce colitis in mice [33] and more recently has been used to establish an enterocolitis model in zebrafish larvae [34]. We injected three different fatty acids (palmitoleic acid, palmitic acid, and linoleic acid) bound to bovine serum albumin (BSA) as a carrier into the tail vein of 3-day post-fertilization zebrafish larvae and measured the inflammatory response by quantification of neutrophil recruitment to the intestine following 3 days of TNBS exposure. In the control animals not exposed to TNBS, injection of BSA alone or any of the fatty acids bound to BSA did not result in any increase in neutrophil recruitment over baseline (Fig. 5; *p* = 0.37 for uninjected versus BSA; all other pairwise comparisons of uninjected versus fatty acid or BSA versus fatty acid were also not significantly different). In these experiments, low dose TNBS exposure in BSA-injected larvae resulted in a moderate increase in inflammation [mean ± standard error of the mean (SEM) neutrophils/intestine of five experiments increased from 33.4 ± 1.6 to 40.6 ± 2.2; *p* = 0.006]. However, neutrophil recruitment to the intestines of TNBS-exposed, palmitic acid-injected larvae was even greater (49.1 ± 2.2; *p* = 0.046 compared with TNBS-exposed, BSA-injected). TNBS-exposed, palmitoleic acid-injected larvae also demonstrated an increase in neutrophil recruitment over TNBS-exposed, BSA-injected, but the increase did not reach statistical significance (45.1 ± 3.0; *p* = 0.16). In contrast, linoleic acid actually resulted in slightly less neutrophil recruitment than BSA with TNBS exposure (37.8 ± 1.4; *p* = 0.03), indicating that different fatty acid species have distinct capacities to modulate intestinal inflammation. The decrease in neutrophil recruitment with linoleic acid is in agreement with past studies indicating that linoleic acid can have an anti-inflammatory effect in Crohn’s disease [35]. In summary, the data fit a model whereby fatty acids are not sufficient to induce intestinal inflammation but can modulate inflammation in the context of the TNBS enterocolitis model. While the specificity of the fatty acid effect was not exactly as we had predicted (i.e., the increase was stronger with palmitic than palmitoleic), the results demonstrate that connections between molecular and disease traits revealed by CPAG can be quickly validated experimentally in animal models. The similarity between palmitoleic acid and Crohn’s disease was the first connection we tested with this CPAG plus model organism approach, but we suspect that further mining CPAG results will reveal additional new connections that warrant further experimental testing (see Additional files 19 and 20 for other potentially novel associations).

### Querying CPAG clusters with user-generated lists

**Fig. 6:**
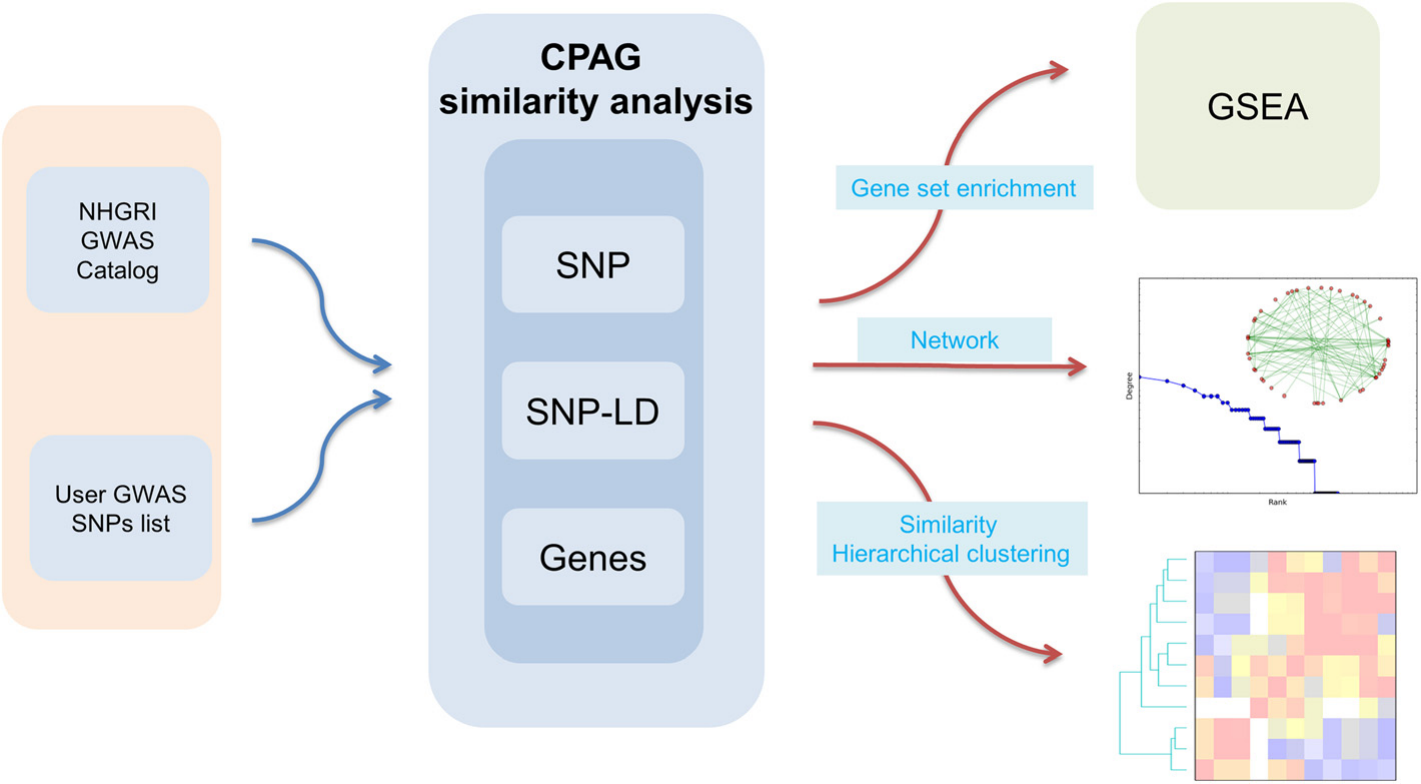
CPAG software. Workflow depicting how CPAG software detects trait similarity and provides a means for visualizing and mining similarity for hypothesis generation. User-generated lists of SNPs are used as input to make comparisons with the entire NHGRI GWAS Catalog based on SNPs, LD-corrected SNPs, or genes. In addition to a text read-out of similarity that includes a description of the SNPs, strength and significance of similarity, and GSEA of pathways underlying the similarity, results can be visualized by hierarchical clustering or by networks. CPAG software can be downloaded at [36]

We packaged all of the functionality described in the preceding sections into a stand-alone CPAG application (Fig. 6; software available at [36]). The software generates similarity matrices, results files of trait similarity, pathway analysis, tree diagrams, and lists of cross-phenotype SNPs and genes using the NHGRI GWAS Catalog. Importantly, we have equipped the software to also incorporate user-generated lists of SNPs. Thus, researchers who generate new GWAS data or any other list of related SNPs can determine which human traits are most related to their list based on the results of all previously published GWAS. For example, previously we carried out GWAS of *Salmonella-*induced cell death (pyroptosis) in 350 human cell lines [37, 38]. Pyroptosis is a pro-inflammatory process mediated by caspase-1 activation by inflammasome complexes [39]. We found that the most significant similarity to the pyroptosis list was observed with early onset myocardial infarction (MI; *p* = 0.003 after Bonferroni correction). Three (of nine) SNPs associated with early onset MI at genome-wide significance were also associated with pyroptosis at the *p* < 0.01 level, and the directions of effect are consistent with a greater pro-inflammatory response being associated with greater risk of early onset MI (Table 1). This directionality is consistent with several published reports implicating inflammation and NLRP3 inflammasome activation of caspase-1 with MI and cardiac reperfusion injury [40–43].

**Table 1:** SNPs associated with both early onset MI and *Salmonella-*induced pyroptosis

			Early onset MI		Salmonella-induced pyroptosis		
SNP	Chromosome	Gene	*P* value	Risk allele	*P* value	High pyroptosis allele	Concordant risk
rs6725887	2	*WDR12*	1.00E-08	C	0.0088	C	Yes
rs9305545, rs9982601	21	-	6.00E-11	T	0.0036	G (T for rs9305545)	Yes
rs12526453, rs2327621	6	*PHACTR1*	1.00E-09	C	0.005	G (? for rs12526453)	?
Summary			Observed	Expected	Enrichment	*P* value	*P* value (Bonferroni)
			3	0.08	37.5	5.9 × 10^−5^	0.003

*P* values for early onset MI association are from the NHGRI GWAS Catalog [1], while *p* values for pyroptosis were from [37]. Two SNPs are given in the same line where the lowest *p* value for early-onset MI and *Salmonella-*induced pyroptosis in the LD region are not the same SNP. The concordance of risk alleles for the two SNPs was determined by examining the direction of association for the early-onset MI SNP in the pyroptosis dataset. SNP rs12526453 shows no association with pyroptosis (despite rs2327621 showing an association), so the direction of effect is undetermined. *P* value for the significance of overlap was calculated with Fisher’s exact test

## Discussion

As the number of human traits that have been studied with genome-wide association has rapidly increased, methods are needed to interpret new studies in light of previous results. Any GWAS meta-analysis approach that combines different studies is limited by heterogeneity in regards to quality of genotyping, subject categorization and phenotypes, population sizes, and definition of traits. However, examining all published GWAS in light of one another creates a valuable opportunity to find unexpected and potentially medically useful connections between an incredible range of phenotypes. The CPAG approach facilitates finding these connections by combining similarity indices from ecology, hierarchical clustering, and gene set enrichment in a format that can be easily explored for biological insight. Combining CPAG with assays in zebrafish and other experimental models allows for rapid hypothesis generation and testing. Validating and characterizing individual instances of overlap will lead to an increased understanding of pleiotropy, shared genetic pathways, and relationships between traits previously thought to be unrelated.

In addition to the heterogeneity of the studies included in CPAG analysis, other limitations for our method are worth pointing out. GWAS to date have primarily been conducted on populations of European ancestry, and therefore are limited to SNP panels and the LD estimates in those populations. Flexibility in setting *p* value thresholds within CPAG is limited by the fact that GWAS often only report the top hits in publications, instead of providing *p* values for all SNPs in the database. The current necessity of setting a *p* value threshold in CPAG points to the possibility of using multivariate approaches incorporating all SNPs or gene–gene and gene–environment interactions into the framework in the future. For our validation studies, the PubMed co-occurrence analysis is limited by stringent text queries that exactly match the categories in the NHGRI GWAS Catalog (with some words such as “traits” being removed). The incorporation of natural language processing could result in a reduction of false positive “novel” associations. In regards to our experimental testing of a CPAG-generated hypothesis, the zebrafish-TNBS exposure model of Crohn's disease recapitulates a microbiota-dependent and pharmacologically responsive enterocolitis with key features of innate immune cell recruitment, cytokine production, and small intestinal shortening. Notably, there are limitations to this model, most importantly, the lack of adaptive immunity and relatively weak changes to epithelial morphology. However, CPAG still represents a substantive advance in identifying and understanding cross-phenotype associations, and improvements will overcome those limitations in future versions.

Hypotheses generated using CPAG could have profound consequences in medicine. Our finding of overlap between plasma palmitoleic acid and Crohn’s disease led us to test the effects of exogenous fatty acids in a zebrafish enterocolitis model. Although the fatty acid specificity we observed was not what we had initially predicted, our results do indicate that some fatty acids could contribute directly to intestinal inflammation. It is important to note that the GWAS for plasma fatty acid levels showing the similarity with Crohn’s disease included only four fatty acids [27]. Similarly, metabolomics of Crohn’s disease have demonstrated lipid abnormalities [31, 32], but broader metabolite panels could potentially reveal more specific, functionally important alterations. Therefore, both more detailed lipidomic GWAS and Crohn’s disease patient profiling, as well as more extensive in vivo testing of other lipid species in models such as zebrafish, are now warranted. We speculate that increasing the depth of our understanding of the dyslipidemias present in Crohn’s disease and the functional effects of individual lipid components on intestinal inflammation could eventually lead to active modulation of plasma fatty acid levels in management of Crohn’s disease. This could be accomplished nutritionally or perhaps by modulating expression of the genes revealed by the shared SNPs between plasma palmitoleic acid and Crohn’s disease. In fact, it has been shown that disruption of one of the genes implicated by the overlapping SNPs, *FADS2* (a fatty acid desaturase), results in both altered lipid profiles as well as the development of intestinal ulcers and inflammation in mice [44].

Similarity of GWAS signatures may also be a means of identifying diseases that could be targeted by the same drugs. Psoriasis and Crohn’s disease sharing variants in the IL-23 pathway as described above is a good example of this. In fact, CPAG also detected that IL-23 pathway variants were also shared with ankylosing spondylitis. Thus, all three autoimmune diseases could perhaps be treated with IL-23 inhibitors.

Possibly the most useful traits identified by CPAG are molecular and cellular traits that could be targeted in treating diseases and/or used as biomarkers in diagnosis/prognosis. We have been using a screening platform called Hi-HOST (high-throughput human in vitro susceptibility testing) to carry out GWAS of cellular host–pathogen phenotypes [37, 38, 45]. In addition to learning about human variation in infectious disease susceptibility, focusing on pathogens allows us to probe variation in basic cellular pathways that have likely been under natural selection in humans. By applying Hi-HOST to a broad range of pathogens and integrating the results with GWAS of disease with CPAG, our goal is to create an interpretive catalog of how human genetic variation affects cell biology to impact disease. While the work presented here demonstrates the utility of CPAG, its full potential will require further contributions from the research community to provide additional molecular and cellular traits that can be connected to disease physiology through cross-phenotype associations.

## Materials and methods

### NHGRI GWAS Catalog data

The data used in our analyses, comprising a total of 11,288 SNPs associated with 886 (raw) traits from 1408 publications, were downloaded from the NHGRI GWAS Catalog [1] on 4 September 2013. To reduce possible false positive hits while retaining the power to detect the greatest number of significantly similar trait pairs, the analyses in this study were done primarily on the subset of SNPs with pre-computed *p* < 1 × 10^−7^ (4737 SNPs). Altering the number of SNPs included in the analysis by relaxing the *p* value threshold (to 10^−5^, 11,284 SNPs) barely changed the number of trait pairs that had significant similarity (defined as *p* < 0.01, Fisher’s exact test with Bonferroni correction) (Additional file 5). We interpret this to mean that the method is robust against additional false positive SNPs introduced in relaxing the *p* value threshold to well below genome-wide significance. In contrast, making the threshold more stringent (to 10^−20^, 813 SNPs) resulted in a progressive reduction in the number of significant trait pairs, limiting the chance for discovery as traits and true-positive SNPs are removed from the analysis (Additional file 5).

Analysis was done on both “raw” traits and “modified” traits made by merging together phenotypes that were subclasses of the same disease (for example, multiple age-related macular degeneration phenotypes were merged) and phenotypes that were closely related (such as systolic blood pressure and diastolic blood pressure). NHGRI GWAS Catalog traits that combined multiple diseases or biomarkers into a single group (such as “Crohn’s disease and celiac disease” or “protein biomarker”) were also excluded. Each trait was assigned to one or two broad categories (autoimmune, infectious disease, cardiovascular/metabolic, body size, eyes, kidneys, nervous system, cancer, or others) based on medical knowledge of the authors prior to running the CPAG analysis (Additional file 21).

### Identification of cross-phenotype SNPs

We used three methods to count the shared SNPs among different traits: 1) overlap by trait-associated SNPs; 2) overlap by SNPs corrected for LD (SNP_LD); and 3) overlap by genes. The LD correction allows for SNPs in high LD to still be counted as overlapping in examining trait pairs and also prevents multiple SNPs in the same LD peak from inflating the observed number of cross-phenotype SNPs. We calculated pairwise LD for all SNPs based on the 1000 Genomes Project [46] CEU population (using PLINK v.1.9 [47]), and counted SNPs as overlapping when *r*^*2*^ > 0.6 and also merged overlapping SNPs into a single group when *r*^*2*^ > 0.6 within a single trait. For overlap by genes, SNP-gene assignments were made based on the “mapped genes” assignment from the NHGRI GWAS Catalog.

### Permutation analysis for calculating abundance of cross-phenotype associations

A permutation method was applied to estimate the abundance of cross-phenotype SNPs in the NHGRI GWAS Catalog. We determined the observed number of cross-phenotype SNPs in the NHGRI GWAS Catalog and compared this with a null distribution. The null distribution was constructed by sampling an equal number of SNPs from HapMap 3 release 2 panel [13], randomly assigning them to traits in the NHGRI GWAS Catalog until reaching the same number of unique associations, determining the number of cross-phenotype associations, and repeating the process 10,000 times. We also carried out the same analyses based on genes, with genes randomly sampled from the human gene pool (22,836 Ensembl coding genes). This analysis was restricted only to SNPs located within genes, as the gene being affected by each SNP is not known with high confidence for most GWAS SNPs and especially intergenic SNPs.

### Diseases similarity indices

To determine the most robust similarity index to use in CPAG, we calculated the similarity matrix using 11 methods (Jaccard [48], Sorensen [49], Chao–Jaccard [17], Chao–Sorensen [17], Morisita [50], Morisita–Horn [51], Pearson correlation coefficient, cosine, Simpson, geometric, and CSI [52]). We primarily used the Chao–Sorensen index [17] to quantify the similarity between two traits. Chao–Sorensen applies a probability model and incorporates the effects of unseen samples (or SNPs/genes).

Given two traits which have *n*_1_ and *n*_2_ associated SNPs, respectively, they have *k* overlapping SNPs (*k* > 0). The probability of *k* overlapping under the hypergeometric distribution is *P*_*k*_*= P*(*X = k|N*_*t*_, *n*_1_, *n*_2_), where:

$$ P\left(X = k\Big|{N}_t,{n}_1,{n}_2\right)=\frac{\left({}_k^{n_2}\right)\left({}_{n_1-k}^{N_t-{n}_2}\right)}{\left({}_{n_1}^{N_t}\right)} $$

Here the *N*_*t*_ represents the total number of SNPs in the NHGRI GWAS Catalog, *n*_1_ and *n*_2_ are the number of SNPs (or genes) associated with the two diseases, and *k* the number overlapping in the sample. The *p* value for ≥ *k* overlapping is equal to:

$$ P\left(X\ge k\right) = 1 - {\sum}_{i=0}^kP\left(X=i\Big|{N}_t,\ {n}_1,{n}_2\right) $$

The similarity indices were calculated as described below:

#### Chao-Sorensen and Chao-Jaccard index

In contrast to traditional methods that depend only on data indicating presence or absence, Chao et al. [17] modified the simple similarity indices (U and V) by considering the abundance of components using a sophistical probabilistic model. Their modified estimates of U and V, which increase robustness by taking into consideration unseen shared components, are:where *X*_*i*_ is the number of SNPs for SNP i for trait 1, *Y*_*i*_ is the number of SNPs for SNP i for trait 2, *k* is the number of shared SNPs for traits 1 and 2, *n*_*1*_ is the total number of SNPs associated with traits 1, *n*_*2*_ is the total number of SNPs associated with traits 2, *f*_*+1*_ is the number of shared SNPs present once for trait 1, *f*_*+2*_ is the number of shared SNPs present twice for trait 1, *f*_*1+*_ is the number of shared SNPs present once for trait 2, and *f*_*2+*_ is the number of shared SNPs present twice for trait 2.

With modified Û and $\widehat{\mathrm{V}}$ , their proposed extended Jaccard estimator is:

$$ {S}_{CJ}=\frac{\widehat{U}\widehat{V}}{\widehat{U}\kern0.5em +\kern0.5em \widehat{V}\kern0.5em -\kern0.5em \widehat{U}\widehat{V}} $$

and extended Sorensen estimator is:

$$ {S}_{CS}=\frac{2\widehat{U}\widehat{V}}{\widehat{U}\kern0.5em +\kern0.5em \widehat{V}} $$

#### Jaccard index

$$ {S}_J=\frac{k}{n_1+{n}_2-k} $$

#### Sorensen index

$$ {\mathrm{S}}_{\mathrm{s}}=\frac{2\mathrm{k}}{{\mathrm{n}}_1+{\mathrm{n}}_2} $$

#### Cosine index

$$ {\mathrm{S}}_{\mathrm{C}}=\frac{\mathrm{k}}{\sqrt{{\mathrm{n}}_1 \times {\mathrm{n}}_2}} $$

#### Simpson index

$$ {S}_{Sim}=\frac{k}{ \min \left({n}_1,{n}_2\right)} $$

#### Geometric index

$$ {\mathrm{S}}_{\mathrm{G}}=\frac{{\mathrm{k}}^2}{\sqrt{{\mathrm{n}}_1 \times {\mathrm{n}}_2}\ } $$

#### Pearson correlation coefficient index

$$ {S}_P=\frac{k\times {n}_y-{n}_1\times {n}_2}{\sqrt{n_1\times {n}_2\times \left({n}_y-{n}_1\right)\times \left({n}_y-{n}_2\right)\ }} $$

where *n*_*y*_ is the total number of SNPs.

#### Connection specificity index

$$ {S}_{CSI} = \frac{\#\  traits\  connected\ to\ 1\  and\ 2\  with\ PCC<\left(PC{C}_{12}-0.05\right)}{N_E} $$

where *N*_*E*_ represents the number of all traits.

#### Morisita index

$$ {S}_M=\frac{2{\displaystyle {\sum}_{i=1}^{S_t}}{X}_i{Y}_i\ }{\frac{n_2}{n_1-1}{\displaystyle {\sum}_{i=1}^{S_t}}{X}_i\left({X}_i-1\right)+\frac{n_1}{n_2-1}{\displaystyle {\sum}_{i=1}^{S_t}}{Y}_i\left({Y}_i-1\right)} $$

where *S*_*t*_ = *n*_*1*_ + *n*_*2*_ − *k* represents the total number of all unique SNPs for traits 1 and 2.

#### Morisita–Horn index

$$ {S}_{MH}=\frac{2{\displaystyle {\sum}_{i=1}^{S_t}}{X}_i{Y}_i\ }{\frac{n_2}{n_1}{\displaystyle {\sum}_{i=1}^{S_t}}{X}_i^2+\frac{n_1}{n_2}{\displaystyle {\sum}_{i=1}^{S_t}}{Y}_i^2\ } $$

where *S*_*t*_ = *n*_*1*_ + *n*_*2*_ − *k* represents the total number of all unique SNPs for traits 1 and 2.

### Clustering of traits and evaluation of heterogeneity

We constructed similarity matrices among all pairwise traits with the above 11 methods. We then applied hierarchical clustering to detect relationships among diseases and identified disease clusters. We used entropy methods to estimate average heterogeneity of hierarchical trees with the Gini–Simpson and Shannon–Wiener index. With K maximum clusters for the tree from each index, we calculated the average heterogeneity using the following equation:

$$ {E}_{\mathrm{K}}=\frac{1}{K}{\displaystyle \sum_{i=1}^K}{E}_{\mathrm{i}} $$

where *E*_*i*_ is the heterogeneity for the i-th cluster.

For the Gini–Simpson entropy index, the total heterogeneity *E*_*i*_ was computed using:

$$ {E}_i=1-{\displaystyle \sum_{\mathrm{j}=1}^{\mathrm{n}}}{P}_{\mathrm{j}}^2 $$

where *P*_*j*_ is the fraction of the j-th distinct pre-defined disease group in cluster i with a total of *n* distinct disease groups.

For the Shannon–Wiener index, the *E*_*i*_ was computed using:

$$ {E}_i=-{\displaystyle \sum_{\mathrm{j}=1}^{\mathrm{n}}}{P}_{\mathrm{j}} \ln {P}_{\mathrm{j}} $$

To cancel effects of varying cluster size (e.g., larger cluster size will have a bias for greater heterogeneity), we also computed the weighted mean *E*_K_ ':

$$ {E}_{\mathrm{K}}\hbox{'}=\frac{1}{\mathrm{K}}{\displaystyle \sum_{i=1}^K}\frac{1}{N_i}{E}_{\mathrm{i}} $$

where *N*_*i*_ is number of traits for the i-th cluster given *K* maximum clusters on the tree. The *E*_i_ ' was calculated with the same methods as *E*_i_.

### Significance of overlap among pairwise diseases

We assessed the significance of overlapping SNPs or genes among each trait pair using two approaches: 1) theoretical *p* values from the hypergeometric distribution, and 2) empirical *p* values from permutation tests. The *p* values were corrected by Bonferroni correction.

The probability of *k* overlapping is depicted as *P*_*k*_ = *P*( *X* = *k*|*N*_*t*_, *n*_1_, *n*_2_), where:

$$ P\left(\ X=k\Big|{N}_t,\kern0.02em {n}_1,\kern0.02em {n}_2\right)=\frac{\left(\begin{array}{c}\hfill {n}_2\hfill \\ {}\hfill k\hfill \end{array}\right)\left(\begin{array}{c}\hfill {N}_t-{n}_2\hfill \\ {}\hfill {n}_1-k\hfill \end{array}\right)}{\left(\begin{array}{c}\hfill {N}_t\hfill \\ {}\hfill {n}_1\hfill \end{array}\right)} $$

where *N*_*t*_ is the total number of SNPs in the NHGRI GWAS Catalog, *n*_1_ and *n*_2_ are the number of SNPs (or genes) associated with the two traits, and *k* the number of overlapping SNPs (or genes) in the sample. The *p* value for more than *k* overlapping is equal to:

$$ P\left(X\ge k\right) = 1 - {\sum}_{i=1}^k{P}_i, $$

which is analogous to the one-tailed Fisher’s exact test.

The expected overlapping $E\left(\overline{k}\right)$ under the hypergeometric distribution is:

$$ E\left(\overline{k}\right) = {n}_1\frac{n_2}{N_t} $$

and the variance is:

$$ V\left(\overline{k}\right) = {n}_1\frac{n_2}{N_t}\frac{N_t - {n}_2}{N_t}\kern0.5em \frac{N_t-{n}_1}{N_t-1} $$

For the empirical *p* value, we randomly sampled *n*_1_ and *n*_2_ SNPs (or genes) from HapMap 3 release 2 panel for traits 1 and 2 and counted the observed overlapping SNPs. We replicated this process for 1000 times to construct the null distribution (and therefore the lowest possible value is *p* < 0.001 in our analysis but the number of permutations could be increased to obtain greater precision in the empirical *p* value). Empirical *p* values were obtained by counting the number of times overlap was more than observed counts.

### PubMed co-occurrence analysis

Trait pairs for which the degree of GWAS similarity was statistically significant based on *p* < 0.05 (Fisher’s exact test, Bonferroni corrected) were queried against PubMed using an in-house python script (available upon request). Filtering prior to analysis included modifying trait names by removing any parenthetical text and any general text indicating measurement, such as the words “level”, “measurements”, “phenotypes”, “plasma”, “biomarkers”, “parameters”, “traits”, and “serum”. Also, “Crohns disease” and “Alzheimers disease” were altered to include their apostrophes to improve the number of PubMed hits for these diseases. Any trait names absent from PubMed were not included in the analysis.

Trait pairs with statistically significant overlap were compared with 10,000 random trait pairs, resampled from raw (or modified) traits of the NHGRI GWAS Catalog. The PubMed co-occurrences for the significant trait pairs and random trait pairs were evaluated by the Mann–Whitney rank sum test. Trait pairs were categorized as ‘novel’ for no co-occurrences of trait pairs, ‘suggestive’ for between 1 and 20 co-occurrences, and ‘well-known’ for trait pairs with more than 20 co-occurrences. PubMed queries were conducted on 20 March 2015.

### Pathway enrichment analyses for trait similarity

Pathway data were downloaded from GSEA/MSigDB [53]. Interferon-induced pathways [54] were also included in the analysis. We used Fisher’s exact test to identify whether the overlapping genes among pairwise traits were enriched in particular pathways. The *p* values were calculated with the following equation:

$$ p = 1-\kern0.5em {\sum}_{i=1}^k\frac{\left(\begin{array}{c}\hfill {n}_a\hfill \\ {}\hfill i\hfill \end{array}\right)\left(\begin{array}{c}\hfill {N}_t-{n}_a\hfill \\ {}\hfill {n}_b-i\ \hfill \end{array}\right)}{\left(\begin{array}{c}\hfill {N}_t\hfill \\ {}\hfill {n}_a\hfill \end{array}\right)}, $$

where *k* represents the number of shared genes for a disease pair overlapping with a pathway *i*, and *n*_*a*_ denotes the number of genes overlapping for each disease pair, and *n*_*b*_ denotes the number of genes in pathway *i*, and *N*_*t*_ represents the total number of human genes (22,836, total number of protein coding genes in Ensembl genes v.75). All *p* values were subjected to Bonferroni correction.

### Zebrafish enterocolitis model

All experiments using zebrafish were performed using protocols approved by the Animal Studies Committee of Duke University Medical Center (protocols A180-11-07 and A165-13-06). This approval process ensures experiments will provide significant new knowledge and are conducted as responsibly and humanely as possible. Analytical standard grade linoleic acid, palmitic acid and palmitoleic acid were purchased from Sigma (62230, 76119, and 76169). BSA Fraction V, 7.5 % solution, was purchased from Gibco (15260–037) and used as a carrier protein to stabilize fatty acids in solution. Linoleic acid was dissolved in 100 % methanol to make a 75 mM stock solution. Palmitic acid and palmitoleic acid were dissolved in 100 % ethanol to make a 75 mM stock solution. Fatty acids were diluted to 7.5 mM in BSA solution, and mixtures were subsequently vortexed for 5 min, aliquoted and stored at −20°C. While zebrafish plasma fatty acid concentrations have not been reported to our knowledge, de Almeida et al. [55] place human plasma fatty acid levels in the millimolar range (with saturated fatty acids measured at 4.5 mM, monounsaturated fatty acids at about 2 mM, and polyunsaturated fatty acids at 6.1 mM). This puts our maximum achievable dose of 7.5 mM within expected physiologic ranges. Additionally, we did not observe neutrophil recruitment in untreated larvae that had been injected with our experimental dose of conjugated fatty acids, suggesting a lack of pathological effect. Transgenic *Tg(lyzC:EGFP)*^*nz117*^ or *Tg(lyzC:DsRed)*^*nz50*^ zebrafish larvae [56] were randomized into treatment groups and injected with 10 nl of 7.5 mM fatty acid containing solution at 3 days post-fertilization intravenously into the posterior caudal vein. Low dose TNBS exposure was carried out to induce weak intestinal inflammation with 30 μg/ml TNBS in E3 media in groups of 10–30 larvae [57]. Larvae were maintained at 28.5°C in a dark incubator. After 3 days of exposure, larvae were anesthetized in tricaine, imaged with epifluorescence on a Zeiss Observer Z1 inverted microscope, and intestinal neutrophils were manually counted.

## Additional files

Additional file 1: Table S1. SNPs exhibiting high levels of cross-phenotype associations. Only SNPs associated with more than five diseases/traits (51 SNPs) are shown. (DOCX 20 kb) Additional file 2: Figure S1. Comparison of SNP, SNP_LD, and gene-based similarity approaches. **a** The fraction of trait pairs with significant overlap is greatest using the SNP_LD method. Trait overlap for the NHGRI GWAS Catalog was evaluated based on exact SNP overlap, SNP overlap taking LD into consideration (SNPs with *r* ^*2*^ > 0.6 are considered overlapping), and by genes (SNPs assigned to genes by NHGRI GWAS Catalog). Significance of overlap was measured using Fisher’s exact test and *p* values were Bonferroni-corrected for multiple-test comparisons. **b** Comparing *p* values for trait pairs reveals lower *p* values using the SNP_LD method. For trait pairs with overlap detected by two of the methods the –log(*p* value) was plotted for each method. While the –log(*p* values) were correlated, they deviated towards greater significance for the SNP_LD method. (PDF 114 kb) Additional file 3: Table S2. CPAG results based on NHGRI GWAS Catalog (last visit on 4 September 2013). Listed are associated disease–trait pairs and counts of overlap based on the SNP, SNP_LD, and gene-based methods. Based on these counts, a series of statistical tests were done to test the significance of the similarity, including Fisher’s exact test, an empirical permutations test (10,000 permutations), hypergeometric test and binomial test. The last three columns list the overlapping genes, SNPs and SNPs in LD. (XLSX 1076 kb) Additional file 4: Figure S2. Hierarchical clustering of NHGRI human traits based on Chao–Sorensen index. The hierarchical dendrogram and heat map of similarity for pairwise human traits were constructed based on the Chao–Sorensen similarity index, and significance of similarity was measured using a hypergeometric test implemented in the CPAG program. Only traits having at least one significant association (*p* < 0.05) against other traits are shown here. Colors in the heat map are based on the similarity index and scaled according to the color key. Colored blocks along the y-axis of the heat map and color of text for trait names are indicative of the nine assigned categories of traits. (PDF 79 kb) Additional file 5: Figure S3. Relationship between the *p* value threshold for SNP inclusion and the number of significant trait pairs discovered by CPAG. Increasing the stringency of the *p* value threshold from 10^−5^ to 10^−20^ decreases the number of SNPs included in the CPAG analysis from 11,284 to 813. A similar number of statistically significant trait pairs is detected for a threshold of 10^−5^ to 10^−8^, but there was a decline in the number of significant pairs as the *p* value threshold was decreased to result in fewer included SNPs. Therefore, the detection of statistically significant pairs is robust against increasing false positive SNPs as the *p* value threshold is made less stringent, while making the *p* value threshold increasingly stringent decreases the discovery of significant trait pairs. (PDF 354 kb) Additional file 6: Figure S4. Relationship between the *p* value threshold for SNP inclusion and trait clustering. **a** Similar clusters for obesity (I), autoimmunity (II), and atherosclerosis (III) are observed with the different *p* value thresholds. Therefore, the detection of informative clusters is robust to varying the number of SNPs in the analysis, although traits are lost as the *p* value threshold is made more stringent. In all cases, the number of clusters (k) in the analysis was set to 20. The locations of the three clusters were also marked in the entire dendrogram and heat map of pairwise human traits for the *p* value threshold of 10^−5^ (**b**), 10^−7^ (**c**) and 10^−10^ (**d**). (ZIP 3188 kb) Additional file 7: Figure S5. Hierarchical clustering of NHGRI human traits based on Chao–Jaccard index. The hierarchical dendrogram and heat map of similarity for pairwise human traits were constructed based on the Chao–Jaccard similarity index, and significance of similarity was measured using a hypergeometric test implemented in the CPAG program. Only traits having at least one significant association (*p* < 0.05) against other traits are shown here. Colors in the heat map are based on the similarity index and scaled according to the color key. Colored blocks along the y-axis of the heat map and color of text for trait names are indicative of the nine assigned categories of traits. (PDF 78 kb) Additional file 8: Figure S6. Hierarchical clustering of NHGRI human traits based on Sorensen index. The hierarchical dendrogram and heat map of similarity for pairwise human traits were constructed based on the Sorensen similarity index, and significance of similarity was measured using a hypergeometric test implemented in the CPAG program. Only traits having at least one significant association (*p* < 0.05) against other traits are shown here. Colors in the heat map are based on the similarity index and scaled according to the color key. Colored blocks along the y-axis of the heat map and color of text for trait names are indicative of the nine assigned categories of traits. (PDF 76 kb) Additional file 9: Figure S7. Hierarchical clustering of NHGRI human traits based on Simpson index. The hierarchical dendrogram and heat map of similarity for pairwise human traits were constructed based on the Simpson similarity index, and significance of similarity was measured using a hypergeometric test implemented in the CPAG program. Only traits having at least one significant association (*p* < 0.05) against other traits are shown here. Colors in the heat map are based on the similarity index and scaled according to the color key. Colored blocks along the y-axis of the heat map and color of text for trait names are indicative of the nine assigned categories of traits. (PDF 75 kb) Additional file 10: Figure S8. Hierarchical clustering of NHGRI human traits based on Pearson correlation coefficient (PCC) index. The hierarchical dendrogram and heat map of similarity for pairwise human traits were constructed based on the PCC similarity index, and significance of similarity was measured using a hypergeometric test implemented in the CPAG program. Only traits having at least one significant association (*p* < 0.05) against other traits are shown here. Colors in the heat map are based on the similarity index and scaled according to the color key. Colored blocks along the y-axis of the heat map and color of text for trait names are indicative of the nine assigned categories of traits. (PDF 82 kb) Additional file 11: Figure S9. Hierarchical clustering of NHGRI human traits based on Morisita-Horn index. The hierarchical dendrogram and heat map of similarity for pairwise human traits were constructed based on the Morisita-Horn similarity index, and significance of similarity was measured using a hypergeometric test implemented in CPAG program. Only traits having at least one significant association (p < 0.05) against other traits are shown here. Colors in the heat map are based on the similarity index and scaled according to the color key. Colored blocks along the y-axis of the heat map and color of text for trait names are indicative of the nine assigned categories of traits. (PDF 67 kb) Additional file 12: Figure S10. Hierarchical clustering of NHGRI human traits based on Jaccard index. The hierarchical dendrogram and heat map of similarity for pairwise human traits were constructed based on the Jaccard similarity index, and significance of similarity was measured using a hypergeometric test implemented in the CPAG program. Only traits having at least one significant association (*p* < 0.05) against other traits are shown here. Colors in the heat map are based on the similarity index and scaled according to the color key. Colored blocks along the y-axis of the heat map and color of text for trait names are indicative of the nine assigned categories of traits. (PDF 75 kb) Additional file 13: Figure S11. Hierarchical clustering of NHGRI human traits based on Morisita index. The hierarchical dendrogram and heat map of similarity for pairwise human traits were constructed based on the Morisita similarity index, and significance of similarity was measured using a hypergeometric test implemented in the CPAG program. Only traits having at least one significant association (*p* < 0.05) against other traits are shown here. Colors in the heat map are based on the similarity index and scaled according to the color key. Colored blocks along the y-axis of the heat map and color of text for trait names are indicative of the nine assigned categories of traits. (PDF 70 kb) Additional file 14: Figure S12. Hierarchical clustering of NHGRI human traits based on connection specificity index (CSI). The hierarchical dendrogram and heat map of similarity for pairwise human traits were constructed based on the CSI, and significance of similarity was measured using a hypergeometric test implemented in the CPAG program. Only traits having at least one significant association (*p* < 0.05) against other traits are shown here. Colors in the heat map are based on the similarity index and scaled according to the color key. Colored blocks along the y-axis of the heat map and color of text for trait names are indicative of the nine assigned categories of traits. (PDF 142 kb) Additional file 15: Figure S13. Hierarchical clustering of NHGRI human traits based on Cosine index. The hierarchical dendrogram and heat map of similarity for pairwise human traits were constructed based on the Cosine similarity index, and significance of similarity was measured using a hypergeometric test implemented in CPAG program. Only traits having at least one significant association (p < 0.05) against other traits are shown here. Colors in the heat map are based on the similarity index and scaled according to the color key. Colored blocks along the y-axis of the heat map and color of text for trait names are indicative of the nine assigned categories of traits. (PDF 77 kb) Additional file 16: Figure S14. Hierarchical clustering of NHGRI human traits based on geometric index. The hierarchical dendrogram and heat map of similarity for pairwise human traits were constructed based on the geometric similarity index, and significance of similarity was measured using a hypergeometric test implemented in the CPAG program. Only traits having at least one significant association (*p* < 0.05) against other traits are shown here. Colors in the heat map are based on the similarity index and scaled according to the color key. Colored blocks along the y-axis of the heat map and color of text for trait names are indicative of the nine assigned categories of traits. (PDF 73 kb) Additional file 17: Figure S15. Entropy-based comparisons of 11 similarity indices. a) Average Gini-Simpson entropy index and Shannon-Wiener entropy index were calculated for each cluster number K for each similarity index. Both entropy indices are unweighted without considering effect of cluster size. The Chao-Sorensen similarity index had the least heterogeneity across different K for both entropy indices. b) Average and median cluster size were plotted against cluster number K. For Chao-Sorensen, the cluster sizes have slight variation while K increases from 2 to 30, and also generates medium to large cluster. We removed the largest cluster of the hierarchical clustering tree from this analysis to reduce possible bias to the average cluster size. (TIFF 346 kb) Additional file 18: Figure S16. Density distributions of non-zero similarity values for inter-group pairwise comparisons and for pairwise comparisons within predefined groups. Pairwise comparisons within seven pre-defined groups generally show higher similarity than pairwise comparisons of traits between the pre-defined groups (inter-group). (PDF 25 kb) Additional file 19: Table S3. Potentially novel raw trait pairs revealed by CPAG and a lack of any co-occurrences in PubMed (last visit on 20 March 2015). The two raw traits with significant similarity (*p* < 0.05, Fisher’s exact test after Bonferroni correction) are listed as “*Trait1*” and “*Trait2*”. The text was modified to remove general terms such as “levels” to broaden the PubMed query (see "Materials and methods"). The number of PubMed hits for each individual trait is given. Out of 741 raw trait pairs with *p* < 0.05, these 43 had no PubMed co-occurrences. The trait pair we tested experimentally in zebrafish (Crohn’s disease and palmitoleic acid plasma levels) is highlighted in *orange*. (DOCX 20 kb) Additional file 20: Table S4. Potentially novel modified trait pairs revealed by CPAG and a lack of any co-occurrences in PubMed (last visit on 20 March 2015). The two modified traits with significant similarity (*p* < 0.05, Fisher’s exact test after Bonferroni correction) are listed as “*Trait1*” and “*Trait2*”. The text was modified to remove general terms such as “levels” to broaden the PubMed query (see "Materials and methods"). The number of PubMed hits for each individual trait is given. Out of 277 modified trait pairs with *p* < 0.05, these 30 had no PubMed co-occurrences. (DOCX 19 kb) Additional file 21: Table S5. NHGRI raw trait names, modified names and their pre-defined groups. NHGRI disease names (“*Raw traits*”) are from the NHGRI GWAS Catalog. Closely related phenotypes were merged and phenotypes in the NHGRI GWAS Catalog that combined multiple diseases were removed for “modified phenotypes”. Each trait was assigned to one or two broad categories (autoimmune, infectious disease, cardiovascular/metabolic, body size, eyes, kidneys, nervous system, cancer, or other) based on medical knowledge of the authors prior to running the CPAG analysis. (DOCX 104 kb)

## Abbreviations

BSA: bovine serum albumin
CPAG: Cross-Phenotype Analysis of GWAS
CSI: connection specificity index
GSEA: gene-set enrichment analysis
GWAS: genome-wide association study
Hi-HOST: high-throughput human in vitro susceptibility testing
IL: interleukin
LD: linkage disequilibrium
MI: myocardial infarction
NHGRI: National Human Genome Research Institute
SEM: standard error of the mean
SNP: single-nucleotide polymorphism
TNBS: trinitrobenzene sulfonic acid
