# Supplementary material for: CPAG: software for leveraging pleiotropy in GWAS to reveal similarity between human traits links plasma fatty acids and intestinal inflammation
Source: Genome Biol. 2015 Sep 15;16(1):190. doi: 10.1186/s13059-015-0722-1 (PMC4570686; doi:10.1186/s13059-015-0722-1)
Supplement: Additional file 1: Table S1. — SNPs exhibiting high levels of cross-phenotype associations. Only SNPs associated with more than five diseases/traits (51 SNPs) are shown. (DOCX 20 kb) [file 13059_2015_722_MOESM1_ESM.docx]

**Table S1**. **SNPs exhibiting high levels of cross-phenotype associations.** 51 SNPs were associated with more than 5 traits in the NHGRI GWAS Catalog.

| rsID | Associated Traits (>=5) | Gene | Chromosome | position | function |
| --- | --- | --- | --- | --- | --- |
| rs1260326 | 17 | GCKR | 2 | 27730940 | missense |
| rs1800562 | 14 | HFE | 6 | 26093141 | intron, missense |
| rs4420638 | 13 | APOC1 | 19 | 45422946 | downstream-500B |
| rs964184 | 12 | ZNF259 | 11 | 1.17E+08 | intron |
| rs780094 | 12 | GCKR | 2 | 27741237 | intron |
| rs3184504 | 11 | SH2B3 | 12 | 1.12E+08 | missense |
| rs3764261 | 10 |  | 16 | 56993324 |  |
| rs2075650 | 10 | TOMM40 | 19 | 45395619 | intron |
| rs855791 | 9 | TMPRSS6 | 22 | 37462936 | missense |
| rs780093 | 9 | GCKR | 2 | 27742603 | intron |
| rs1805007 | 9 | MC1R | 16 | 89986117 | missense |
| rs12203592 | 9 | IRF4 | 6 | 396321 | intron |
| rs174547 | 8 | FADS1 | 11 | 61570783 | intron |
| rs7903146 | 7 | TCF7L2 | 10 | 1.15E+08 | intron |
| rs7775698 | 7 |  | 6 | 1.35E+08 |  |
| rs671 | 7 | ALDH2 | 12 | 1.12E+08 | missense |
| rs2736100 | 7 | TERT | 5 | 1286516 | intron |
| rs2074356 | 7 | HECTD4 | 12 | 1.13E+08 | intron |
| rs102275 | 7 | TMEM258 | 11 | 61557803 | intron |
| rs9987289 | 6 | LOC157273 | 8 | 9183358 | intron |
| rs9399137 | 6 |  | 6 | 1.35E+08 |  |
| rs7574865 | 6 | STAT4 | 2 | 1.92E+08 | intron |
| rs653178 | 6 | ATXN2 | 12 | 1.12E+08 | intron |
| rs6511720 | 6 | LDLR | 19 | 11202306 | intron |
| rs646776 | 6 | CELSR2 | 1 | 1.1E+08 | downstream-500B |
| rs560887 | 6 | G6PC2 | 2 | 1.7E+08 | intron |
| rs505922 | 6 |  | 9 | 1.36E+08 |  |
| rs2476601 | 6 | PTPN22 | 1 | 1.14E+08 | missense |
| rs173539 | 6 |  | 16 | 56988044 |  |
| rs1532085 | 6 |  | 15 | 58683366 |  |
| rs1393350 | 6 | TYR | 11 | 89011046 | intron |
| rs13107325 | 6 | SLC39A8 | 4 | 1.03E+08 | missense |
| rs12913832 | 6 | HERC2 | 15 | 28365618 | intron |
| rs867186 | 5 | PROCR | 20 | 33764554 | missense |
| rs8050136 | 5 | FTO | 16 | 53816275 | intron |
| rs7524102 | 5 |  | 1 | 22698447 |  |
| rs7203560 | 5 | NPRL3 | 16 | 184390 | intron |
| rs579459 | 5 |  | 9 | 1.36E+08 |  |
| rs445925 | 5 |  | 19 | 45415640 |  |
| rs401681 | 5 | CLPTM1L | 5 | 1322087 | intron |
| rs3024505 | 5 |  | 1 | 2.07E+08 |  |
| rs2108622 | 5 | CYP4F2 | 19 | 15990431 | missense |
| rs1893217 | 5 | PTPN2 | 18 | 12809340 | intron |
| rs174546 | 5 | FADS1 | 11 | 61569830 | UTR-3’ |
| rs12896399 | 5 |  | 14 | 92773663 |  |
| rs11209026 | 5 | IL23R | 1 | 67705958 | missense |
| rs11066280 | 5 | HECTD4 | 12 | 1.13E+08 | intron |
| rs11065987 | 5 |  | 12 | 1.12E+08 |  |
| rs10830963 | 5 | MTNR1B | 11 | 92708710 | intron |
| rs10468017 | 5 |  | 15 | 58678512 |  |
| rs10455872 | 5 | LPA | 6 | 1.61E+08 | intron |
