# Supplementary figures and images for: CPAG: software for leveraging pleiotropy in GWAS to reveal similarity between human traits links plasma fatty acids and intestinal inflammation
Source: Genome Biol. 2015 Sep 15;16(1):190. doi: 10.1186/s13059-015-0722-1 (PMC4570686; doi:10.1186/s13059-015-0722-1)

**a**Fraction of overlapping trait pairs with  $p < 0.05$ 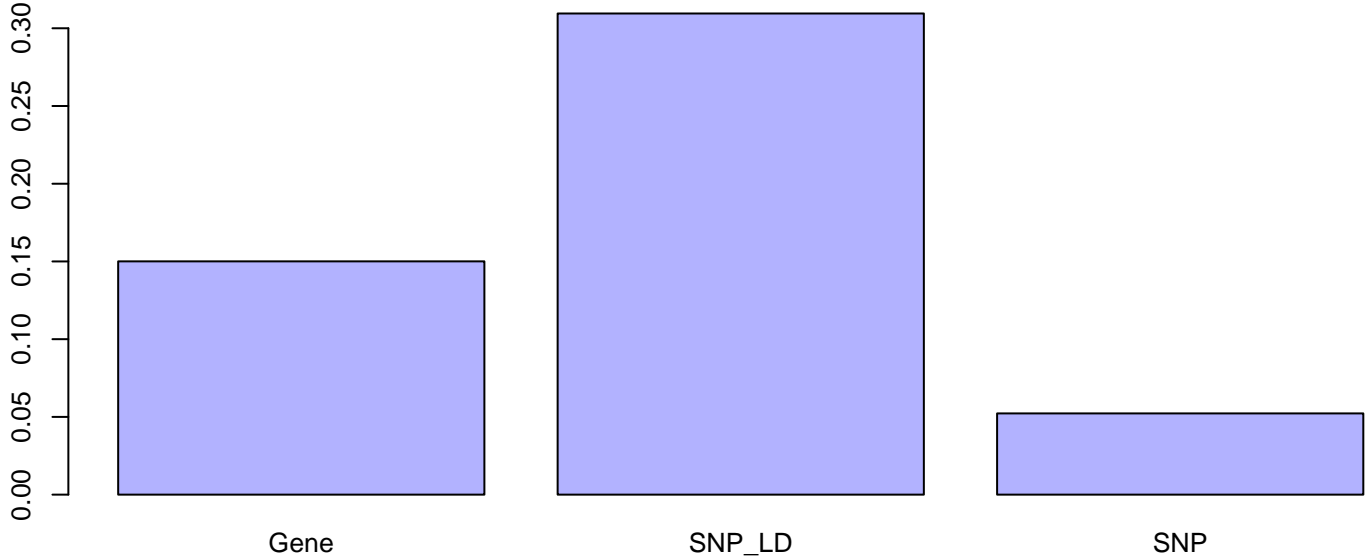**b**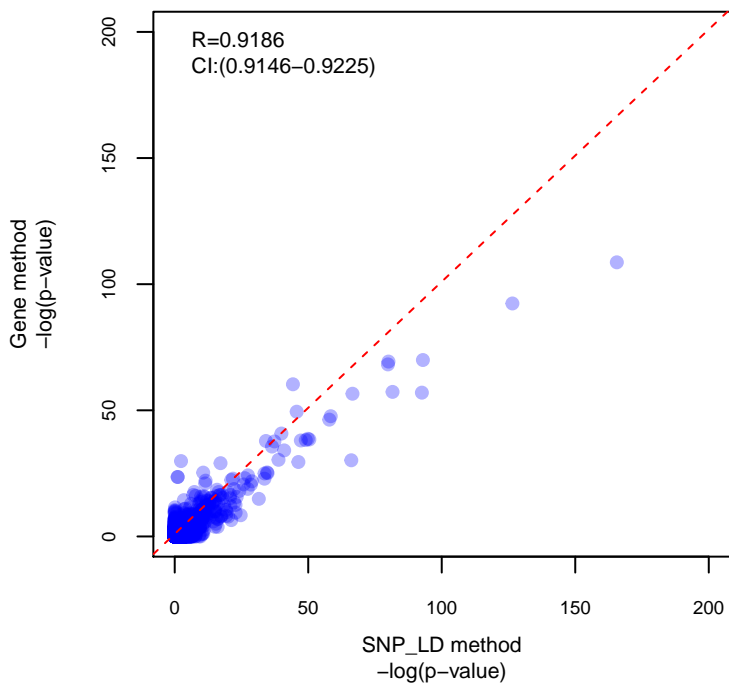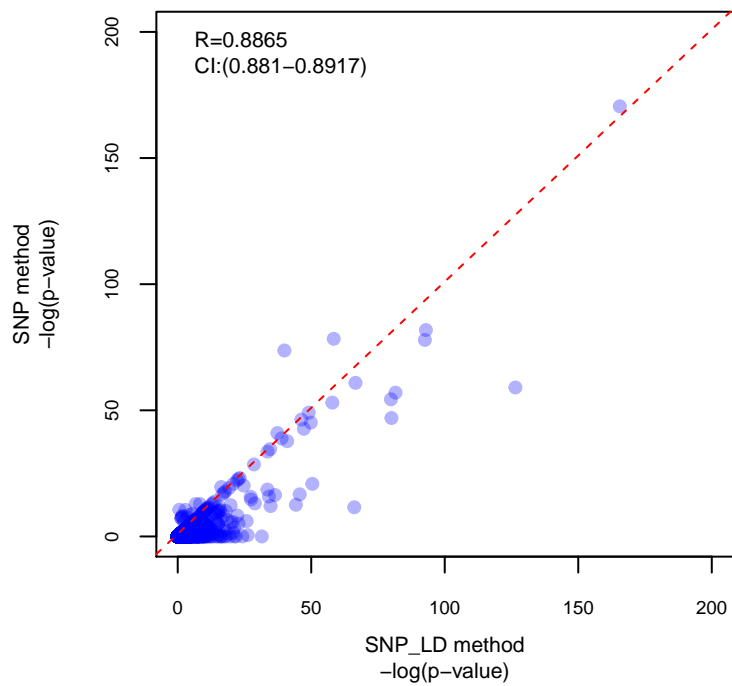

Supplement: Additional file 2: Figure S1. — Comparison of SNP, SNP_LD, and gene-based similarity approaches. a The fraction of trait pairs with significant overlap is greatest using the SNP_LD method. Trait overlap for the NHGRI GWAS Catalog was evaluated based on exact SNP overlap, SNP overlap taking LD into consideration (SNPs with r 2 > 0.6 are considered overlapping), and by genes (SNPs assigned to genes by NHGRI GWAS Catalog). Significance of overlap was measured using Fisher’s exact test and p values were Bonferroni-corrected for multiple-test comparisons. b Comparing p values for trait pairs reveals lower p values using the SNP_LD method. For trait pairs with overlap detected by two of the methods the –log(p value) was plotted for each method. While the –log(p values) were correlated, they deviated towards greater significance for the SNP_LD method. (PDF 114 kb) [file 13059_2015_722_MOESM2_ESM.pdf]

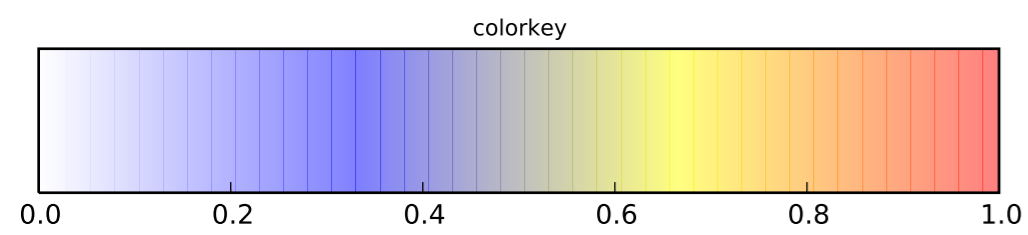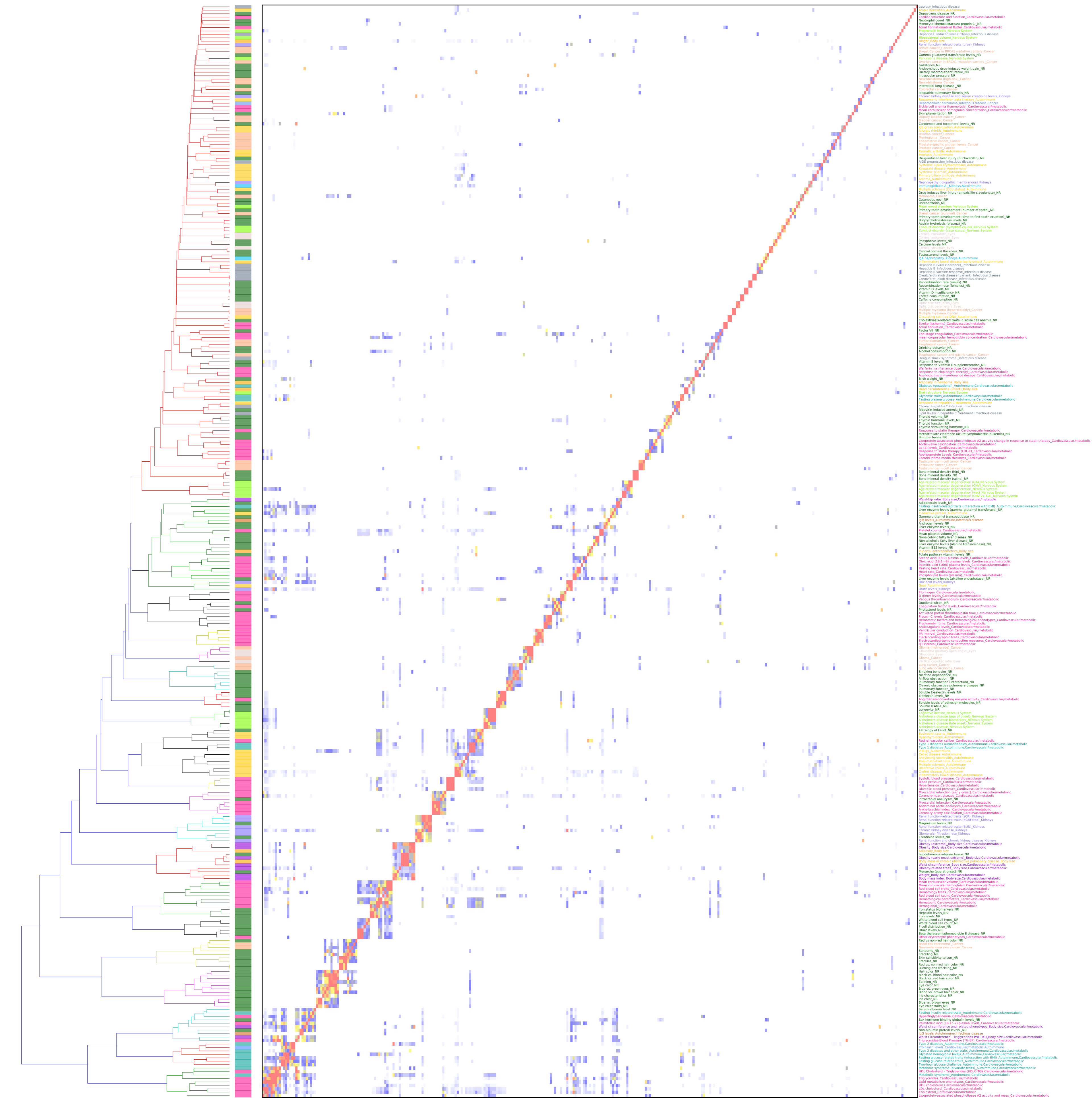

Supplement: Additional file 4: Figure S2. — Hierarchical clustering of NHGRI human traits based on Chao–Sorensen index. The hierarchical dendrogram and heat map of similarity for pairwise human traits were constructed based on the Chao–Sorensen similarity index, and significance of similarity was measured using a hypergeometric test implemented in the CPAG program. Only traits having at least one significant association (p < 0.05) against other traits are shown here. Colors in the heat map are based on the similarity index and scaled according to the color key. Colored blocks along the y-axis of the heat map and color of text for trait names are indicative of the nine assigned categories of traits. (PDF 79 kb) [file 13059_2015_722_MOESM4_ESM.pdf]

Number of SNPs

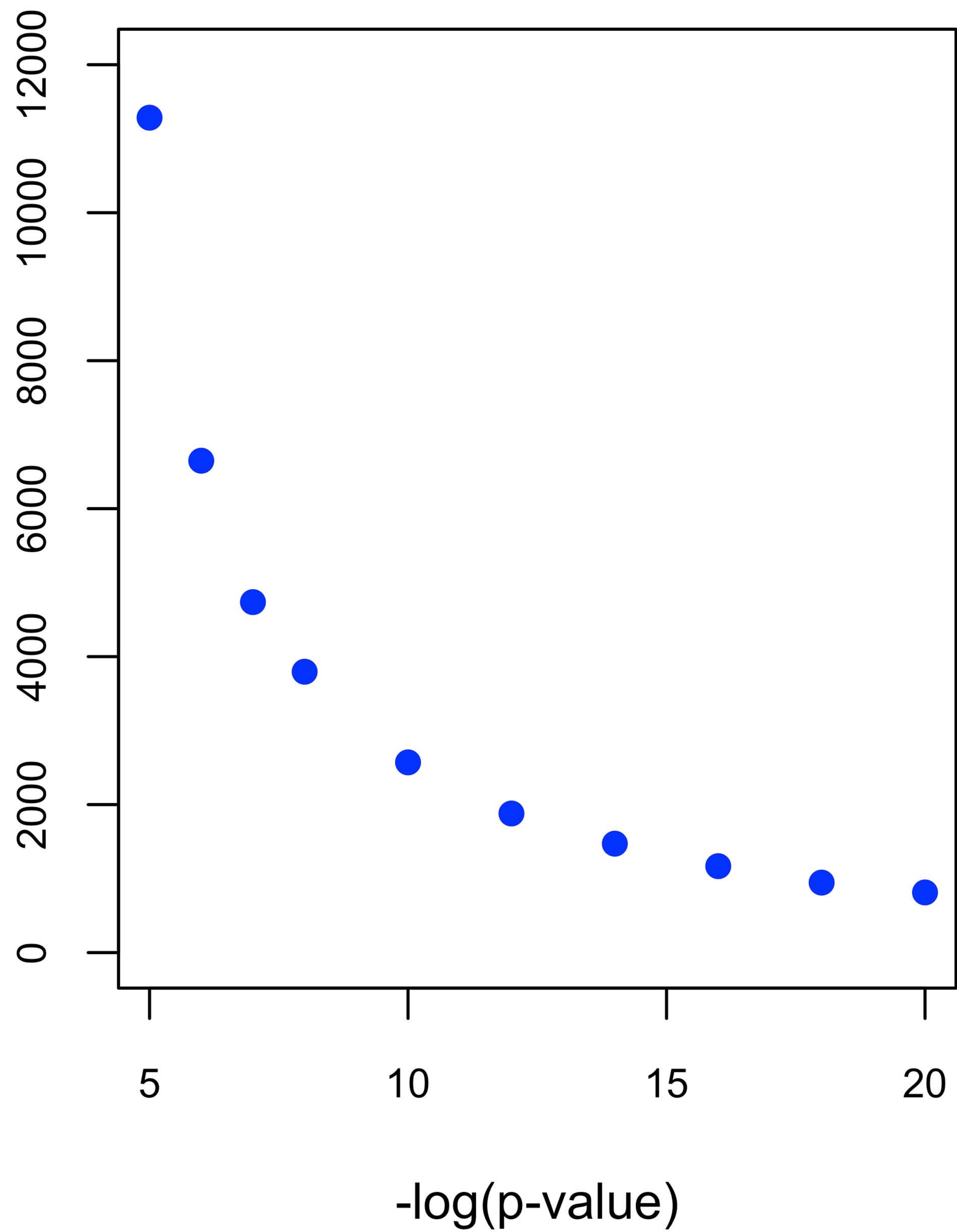

Number of trait pairs ( $p < 0.01$ )

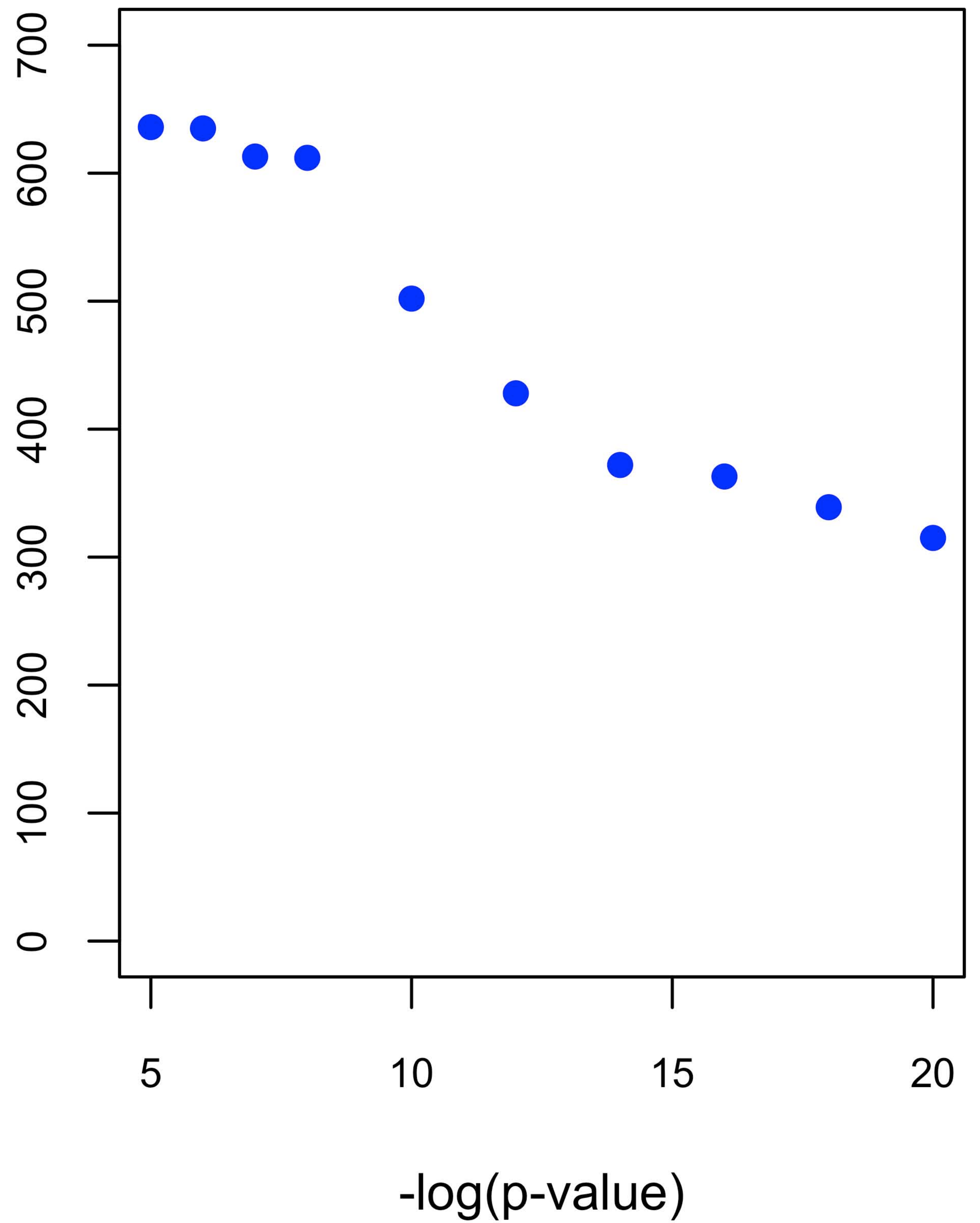

Supplement: Additional file 5: Figure S3. — Relationship between the p value threshold for SNP inclusion and the number of significant trait pairs discovered by CPAG. Increasing the stringency of the p value threshold from 10−5 to 10−20 decreases the number of SNPs included in the CPAG analysis from 11,284 to 813. A similar number of statistically significant trait pairs is detected for a threshold of 10−5 to 10−8, but there was a decline in the number of significant pairs as the p value threshold was decreased to result in fewer included SNPs. Therefore, the detection of statistically significant pairs is robust against increasing false positive SNPs as the p value threshold is made less stringent, while making the p value threshold increasingly stringent decreases the discovery of significant trait pairs. (PDF 354 kb) [file 13059_2015_722_MOESM5_ESM.pdf]

# SNPs

included:  $p < 1 \times 10^{-5}$

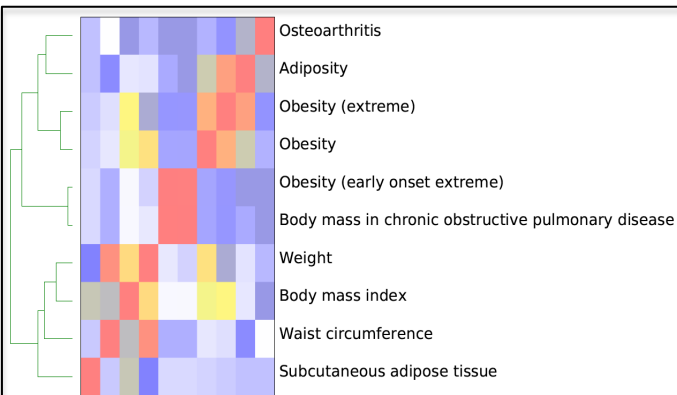

$p < 1 \times 10^{-7}$

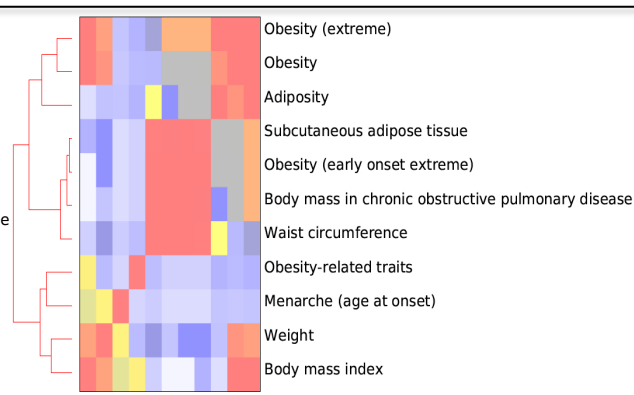

$p < 1 \times 10^{-10}$

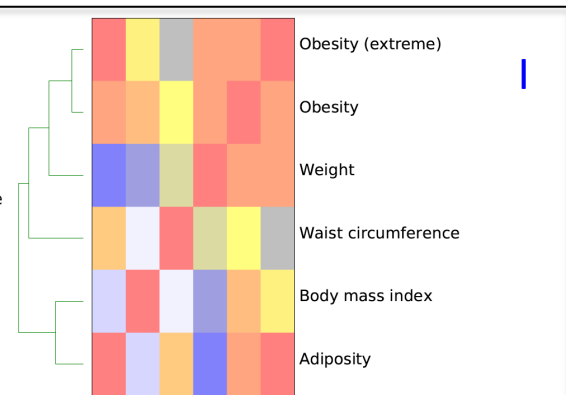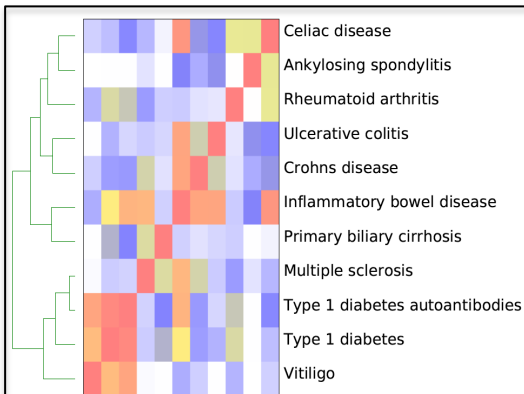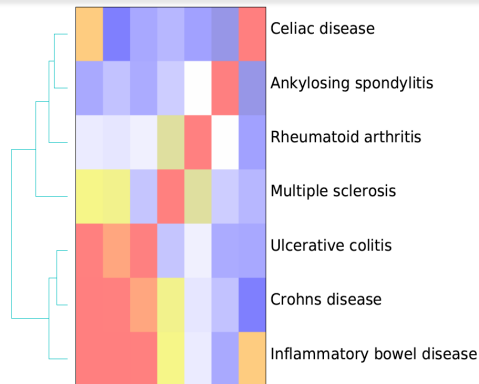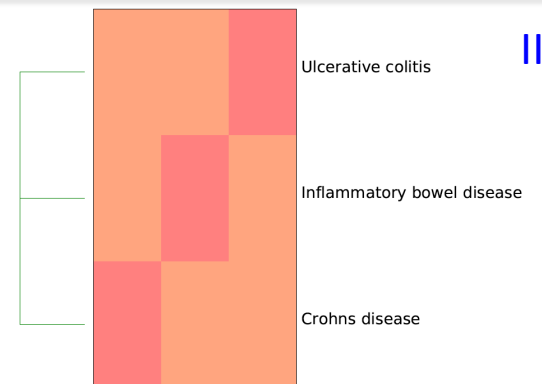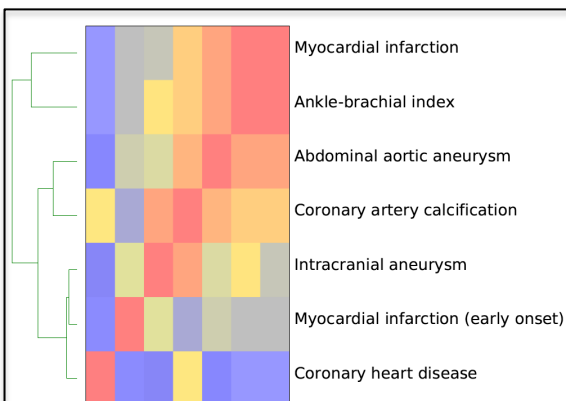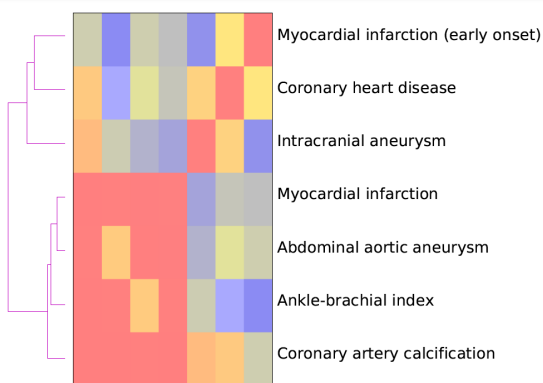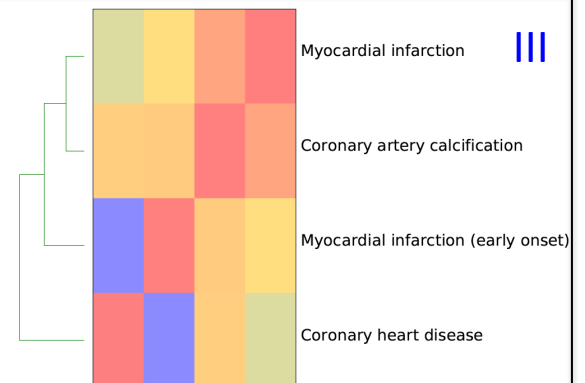

Supplement: Additional file 6: Figure S4. — Relationship between the p value threshold for SNP inclusion and trait clustering. a Similar clusters for obesity (I), autoimmunity (II), and atherosclerosis (III) are observed with the different p value thresholds. Therefore, the detection of informative clusters is robust to varying the number of SNPs in the analysis, although traits are lost as the p value threshold is made more stringent. In all cases, the number of clusters (k) in the analysis was set to 20. The locations of the three clusters were also marked in the entire dendrogram and heat map of pairwise human traits for the p value threshold of 10−5 (b), 10−7 (c) and 10−10 (d). (ZIP 3188 kb) [file 13059_2015_722_MOESM6_ESM.zip › 13059_2015_722_add4.pdf]

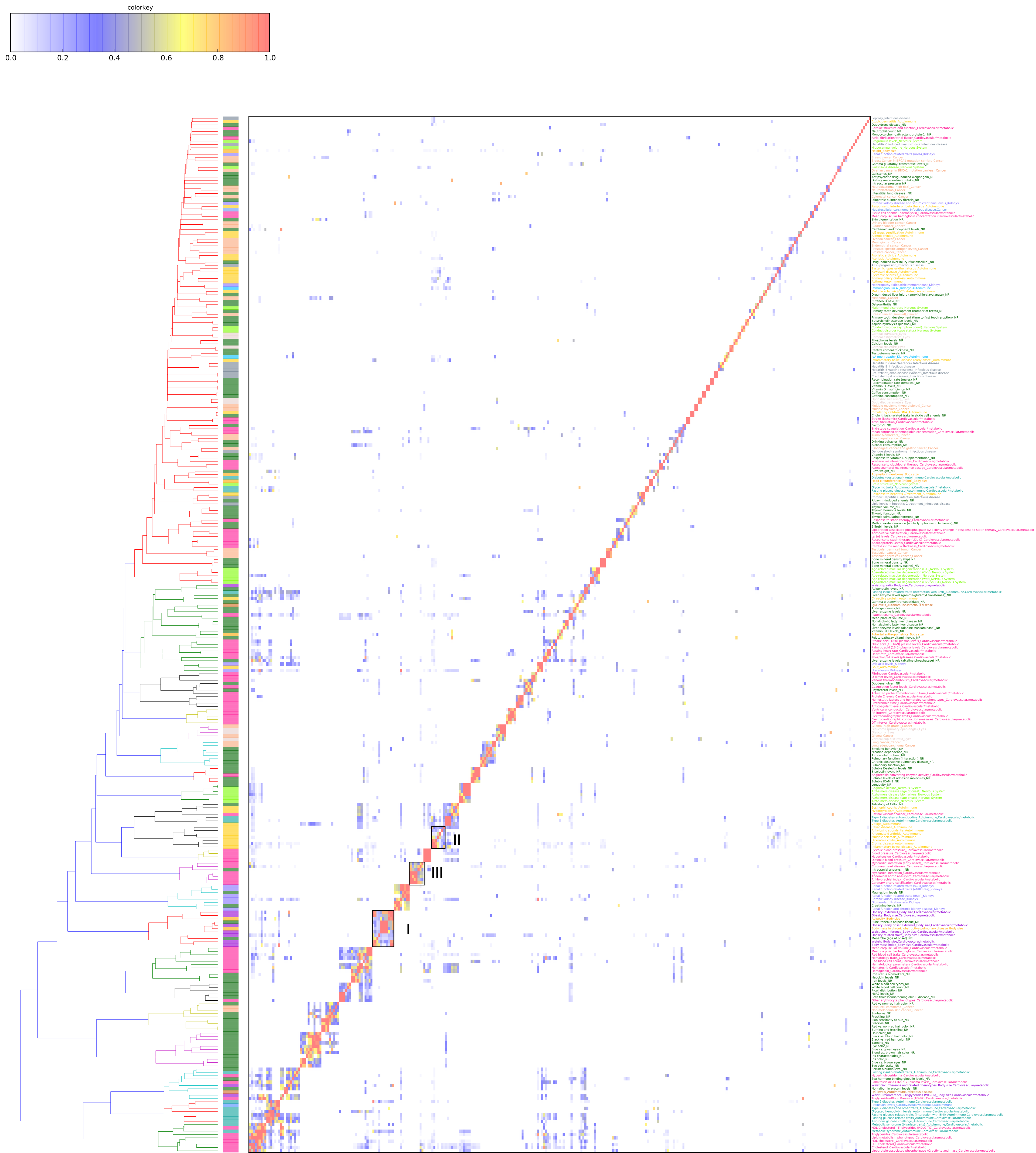

Supplement: Additional file 6: Figure S4. — Relationship between the p value threshold for SNP inclusion and trait clustering. a Similar clusters for obesity (I), autoimmunity (II), and atherosclerosis (III) are observed with the different p value thresholds. Therefore, the detection of informative clusters is robust to varying the number of SNPs in the analysis, although traits are lost as the p value threshold is made more stringent. In all cases, the number of clusters (k) in the analysis was set to 20. The locations of the three clusters were also marked in the entire dendrogram and heat map of pairwise human traits for the p value threshold of 10−5 (b), 10−7 (c) and 10−10 (d). (ZIP 3188 kb) [file 13059_2015_722_MOESM6_ESM.zip › 13059_2015_722_add4c.pdf]

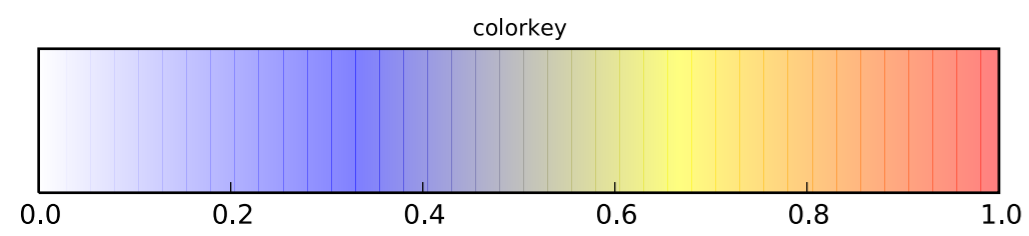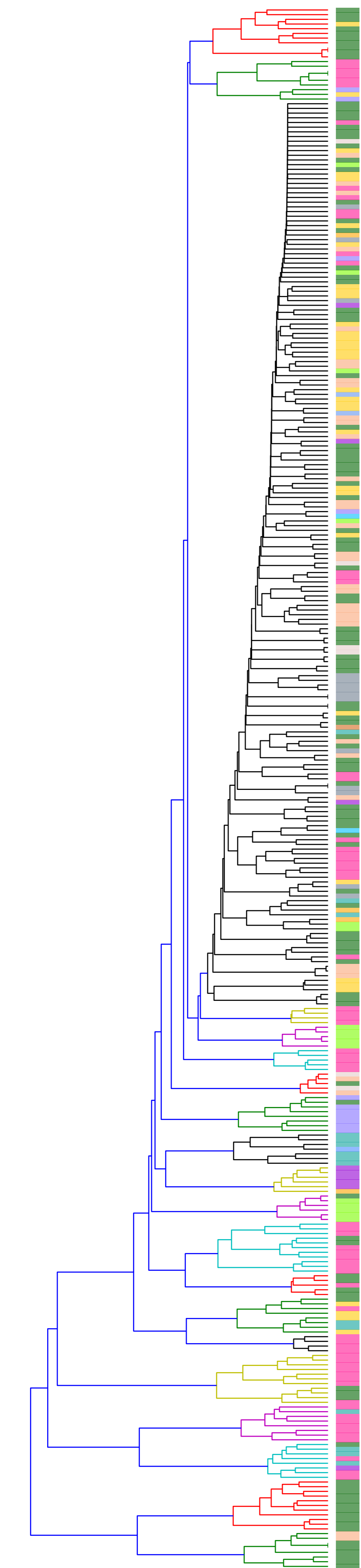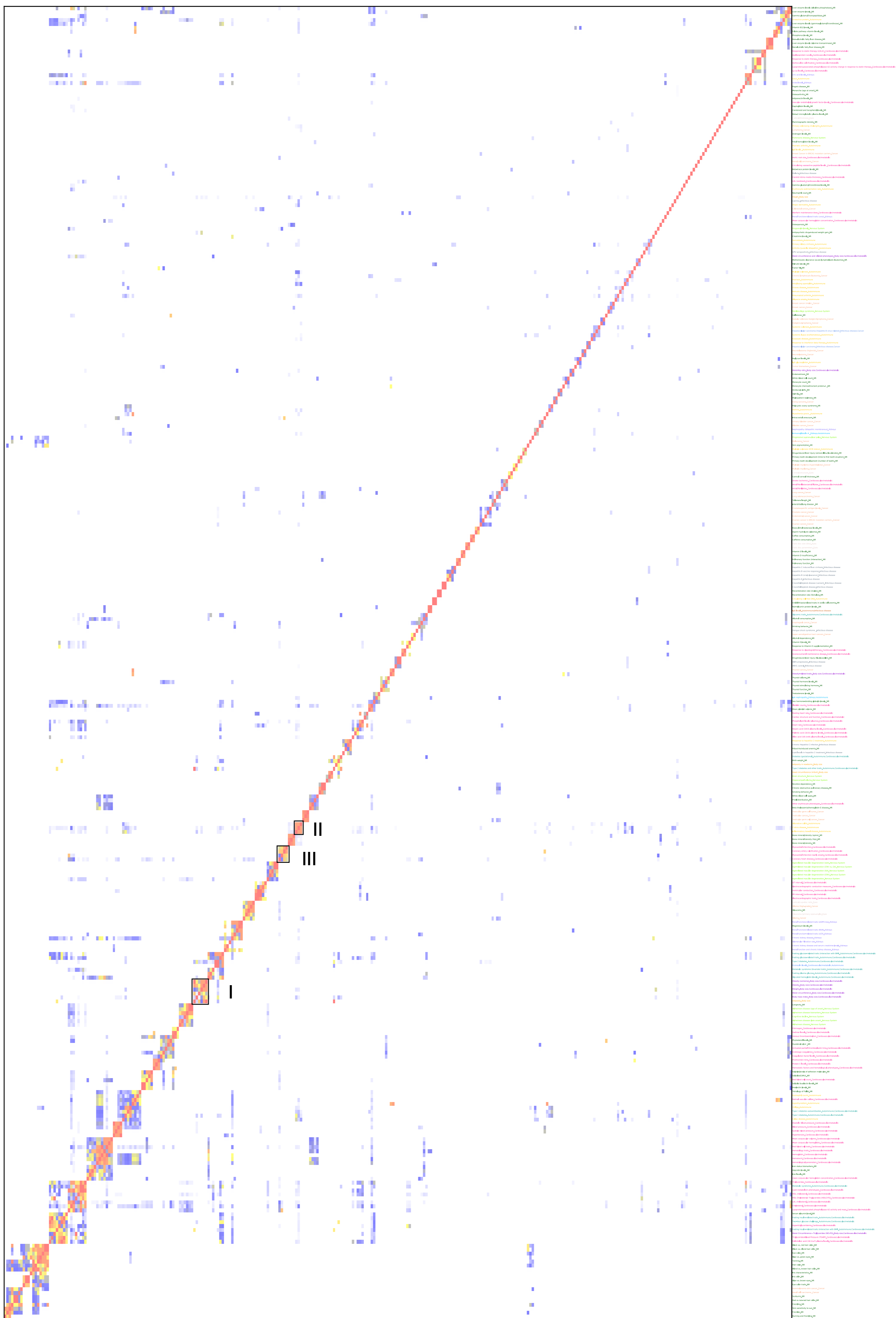

Supplement: Additional file 6: Figure S4. — Relationship between the p value threshold for SNP inclusion and trait clustering. a Similar clusters for obesity (I), autoimmunity (II), and atherosclerosis (III) are observed with the different p value thresholds. Therefore, the detection of informative clusters is robust to varying the number of SNPs in the analysis, although traits are lost as the p value threshold is made more stringent. In all cases, the number of clusters (k) in the analysis was set to 20. The locations of the three clusters were also marked in the entire dendrogram and heat map of pairwise human traits for the p value threshold of 10−5 (b), 10−7 (c) and 10−10 (d). (ZIP 3188 kb) [file 13059_2015_722_MOESM6_ESM.zip › 13059_2015_722_add4d.pdf]

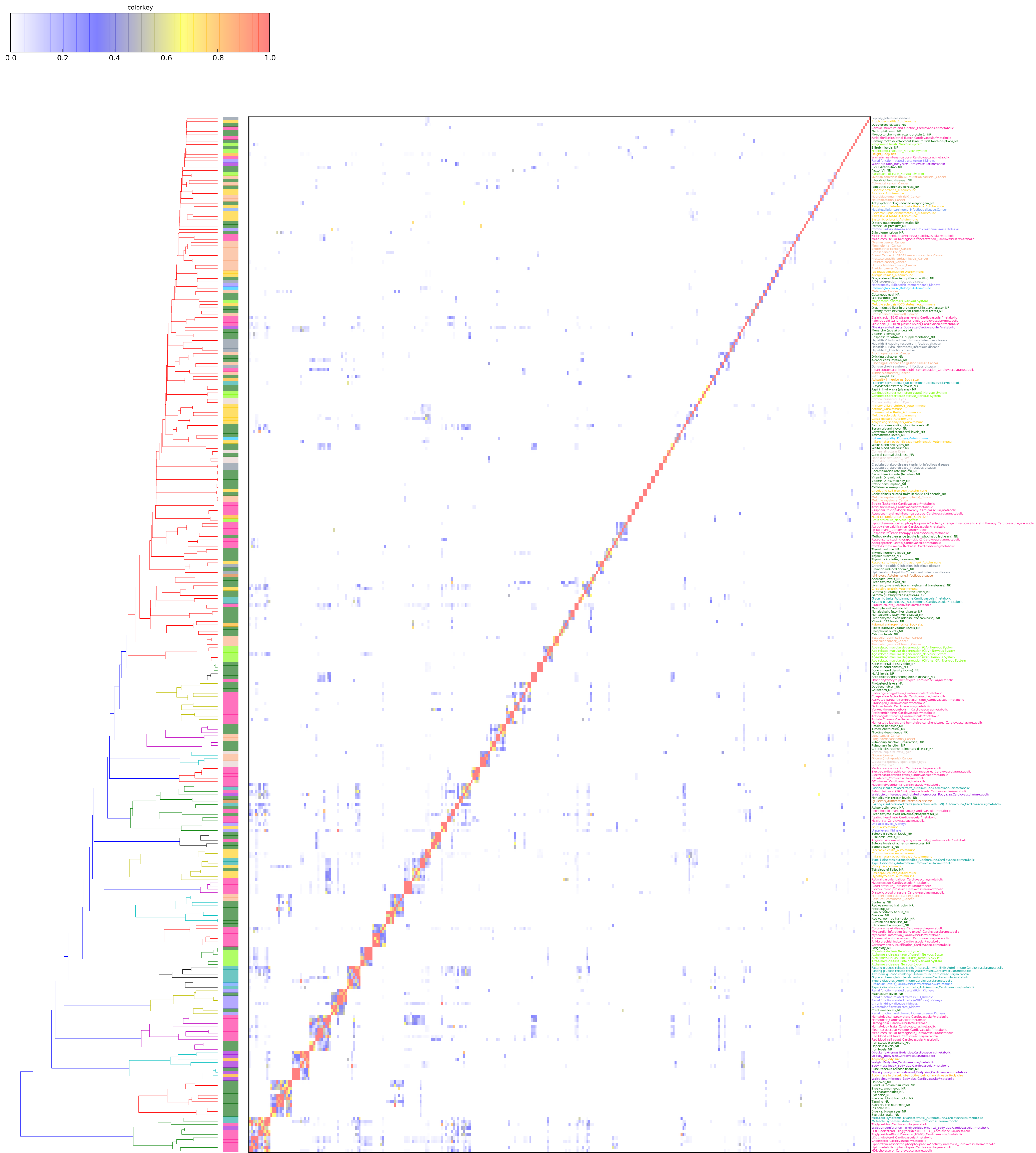

Supplement: Additional file 7: Figure S5. — Hierarchical clustering of NHGRI human traits based on Chao–Jaccard index. The hierarchical dendrogram and heat map of similarity for pairwise human traits were constructed based on the Chao–Jaccard similarity index, and significance of similarity was measured using a hypergeometric test implemented in the CPAG program. Only traits having at least one significant association (p < 0.05) against other traits are shown here. Colors in the heat map are based on the similarity index and scaled according to the color key. Colored blocks along the y-axis of the heat map and color of text for trait names are indicative of the nine assigned categories of traits. (PDF 78 kb) [file 13059_2015_722_MOESM7_ESM.pdf]

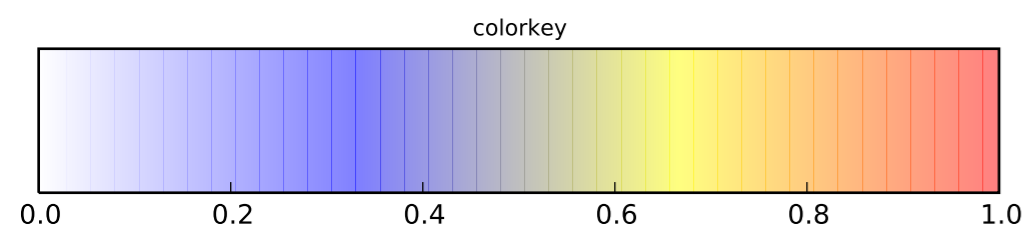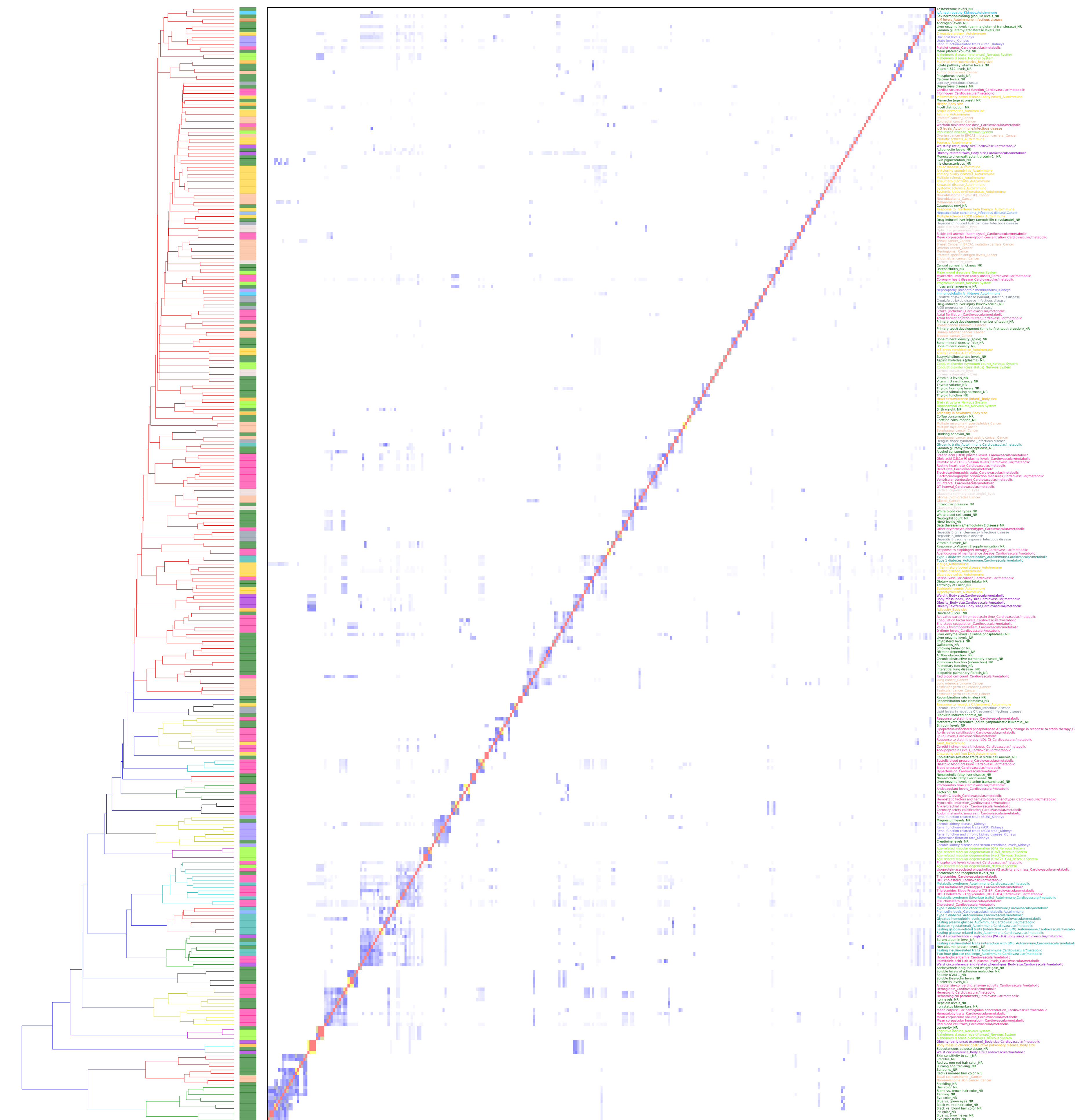

Supplement: Additional file 8: Figure S6. — Hierarchical clustering of NHGRI human traits based on Sorensen index. The hierarchical dendrogram and heat map of similarity for pairwise human traits were constructed based on the Sorensen similarity index, and significance of similarity was measured using a hypergeometric test implemented in the CPAG program. Only traits having at least one significant association (p < 0.05) against other traits are shown here. Colors in the heat map are based on the similarity index and scaled according to the color key. Colored blocks along the y-axis of the heat map and color of text for trait names are indicative of the nine assigned categories of traits. (PDF 76 kb) [file 13059_2015_722_MOESM8_ESM.pdf]

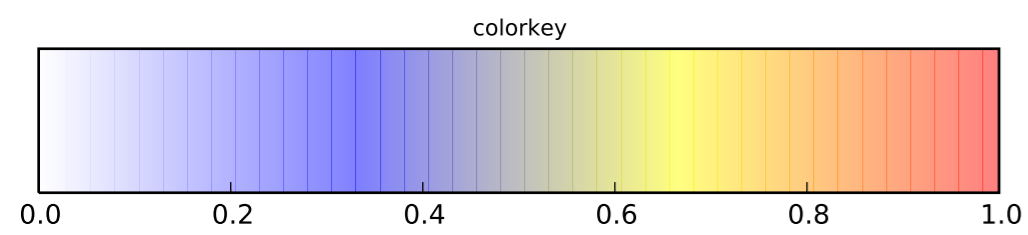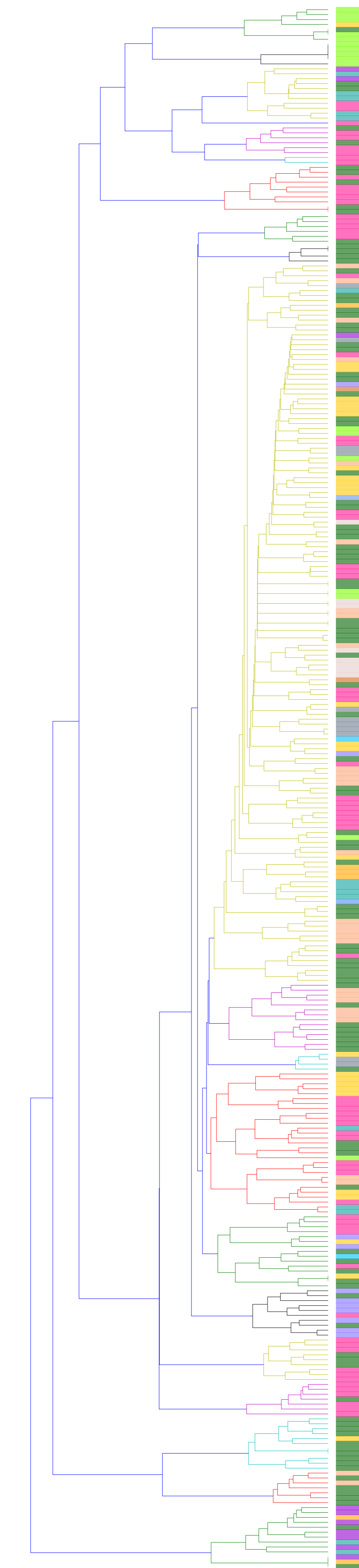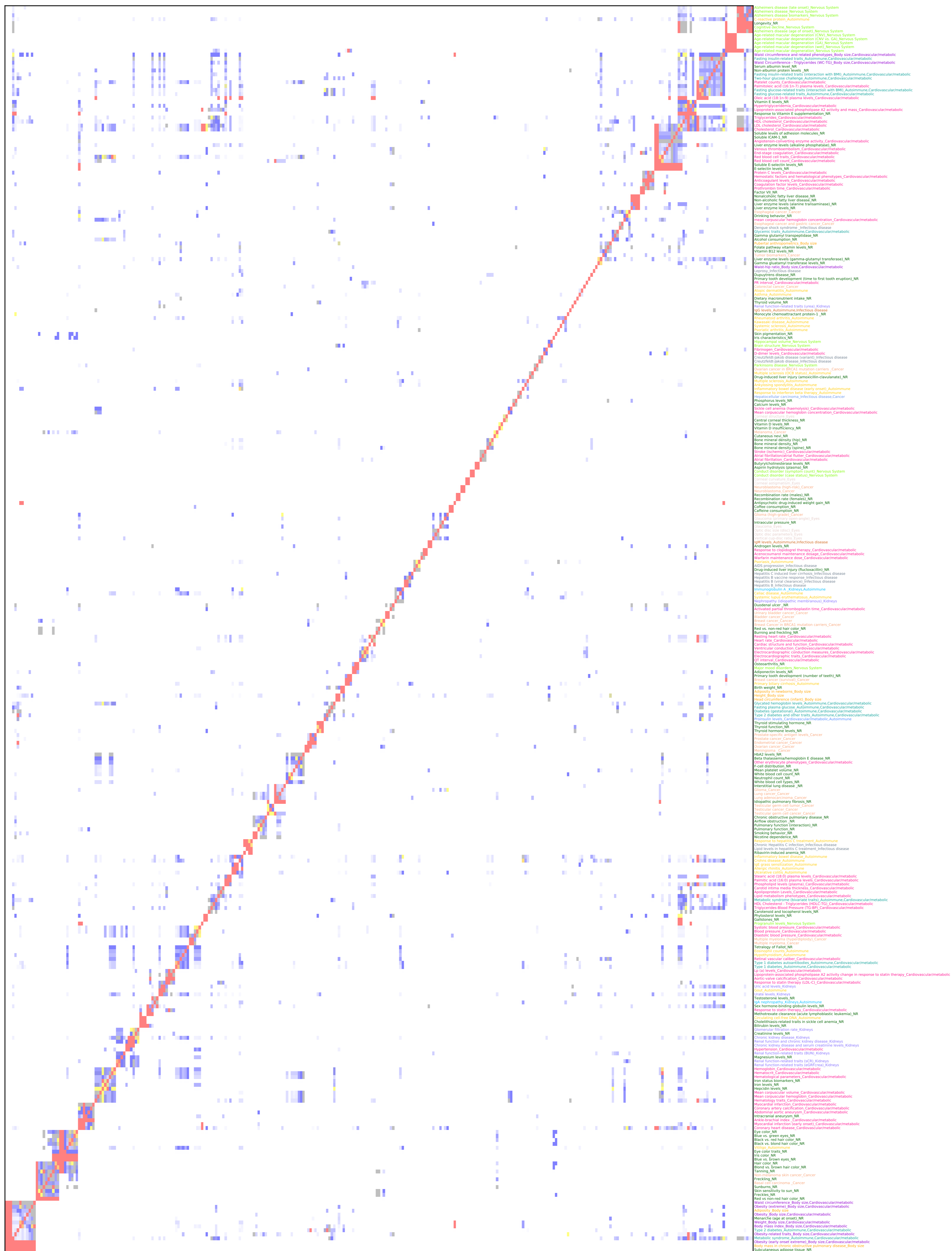

Supplement: Additional file 9: Figure S7. — Hierarchical clustering of NHGRI human traits based on Simpson index. The hierarchical dendrogram and heat map of similarity for pairwise human traits were constructed based on the Simpson similarity index, and significance of similarity was measured using a hypergeometric test implemented in the CPAG program. Only traits having at least one significant association (p < 0.05) against other traits are shown here. Colors in the heat map are based on the similarity index and scaled according to the color key. Colored blocks along the y-axis of the heat map and color of text for trait names are indicative of the nine assigned categories of traits. (PDF 75 kb) [file 13059_2015_722_MOESM9_ESM.pdf]

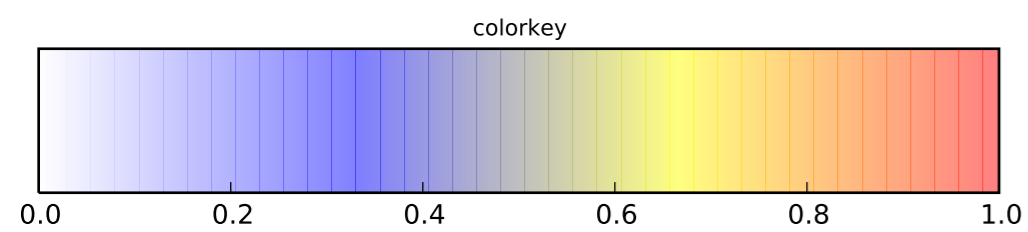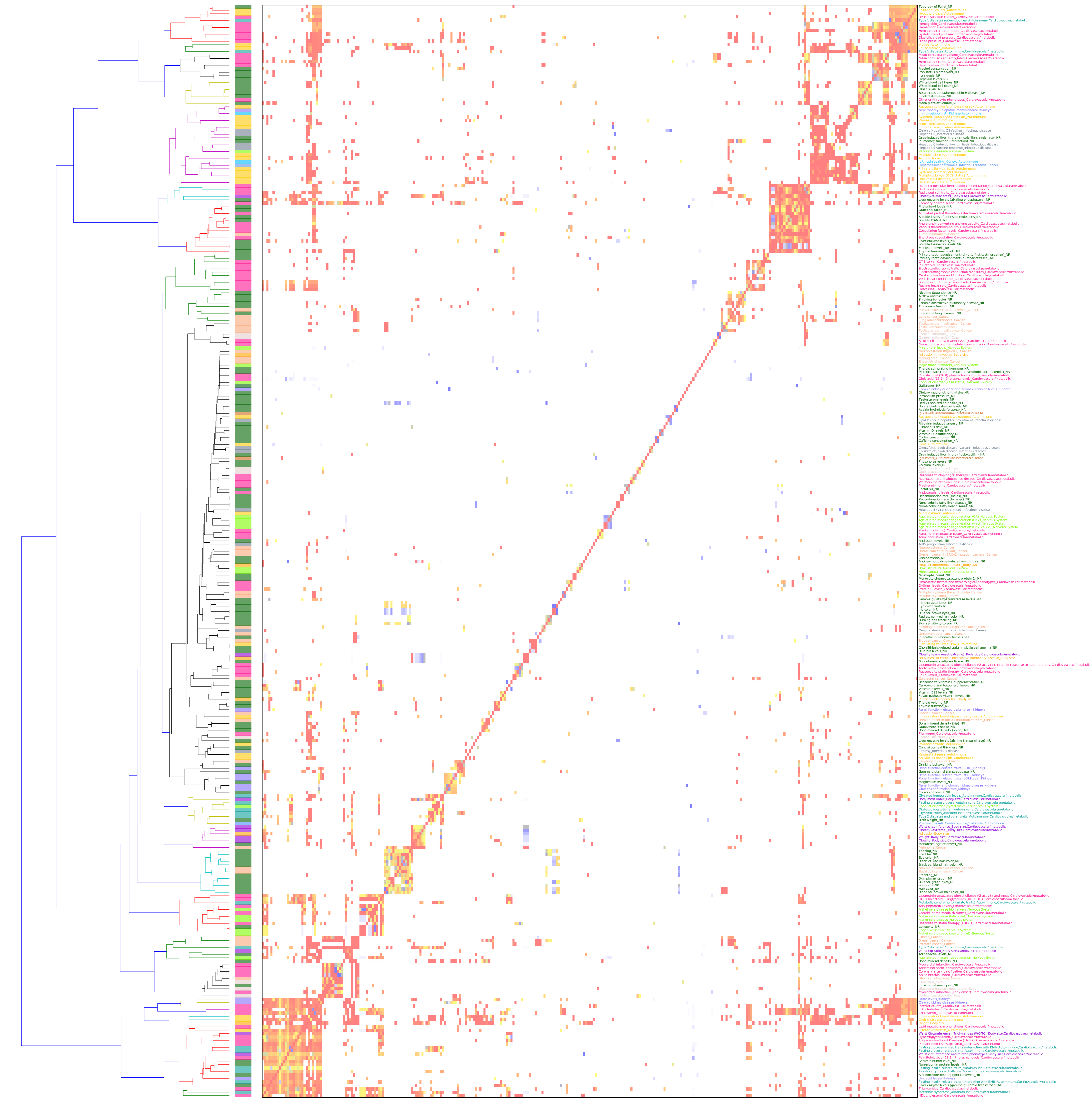

Supplement: Additional file 10: Figure S8. — Hierarchical clustering of NHGRI human traits based on Pearson correlation coefficient (PCC) index. The hierarchical dendrogram and heat map of similarity for pairwise human traits were constructed based on the PCC similarity index, and significance of similarity was measured using a hypergeometric test implemented in the CPAG program. Only traits having at least one significant association (p < 0.05) against other traits are shown here. Colors in the heat map are based on the similarity index and scaled according to the color key. Colored blocks along the y-axis of the heat map and color of text for trait names are indicative of the nine assigned categories of traits. (PDF 82 kb) [file 13059_2015_722_MOESM10_ESM.pdf]

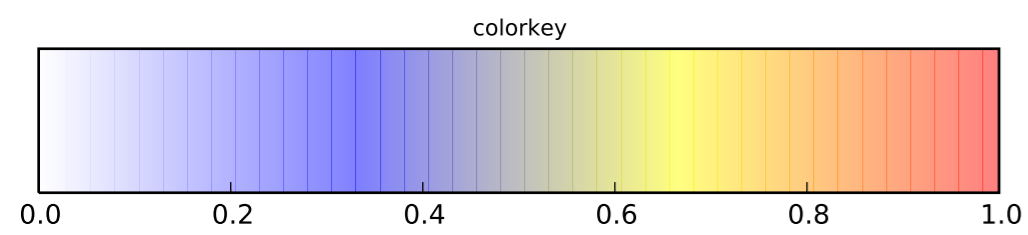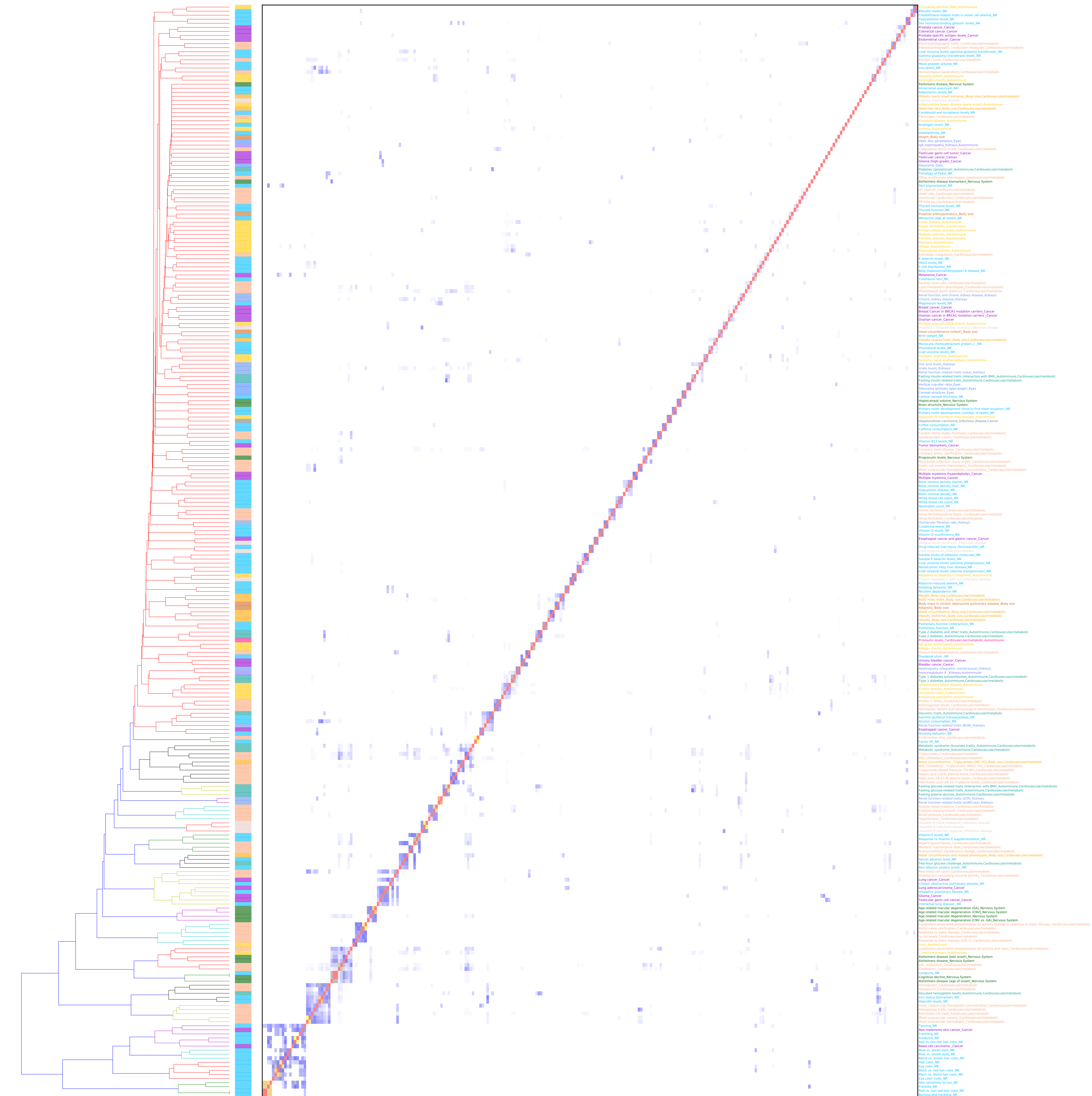

Supplement: Additional file 11: Figure S9. — Hierarchical clustering of NHGRI human traits based on Morisita-Horn index. The hierarchical dendrogram and heat map of similarity for pairwise human traits were constructed based on the Morisita-Horn similarity index, and significance of similarity was measured using a hypergeometric test implemented in CPAG program. Only traits having at least one significant association (p < 0.05) against other traits are shown here. Colors in the heat map are based on the similarity index and scaled according to the color key. Colored blocks along the y-axis of the heat map and color of text for trait names are indicative of the nine assigned categories of traits. (PDF 67 kb) [file 13059_2015_722_MOESM11_ESM.pdf]

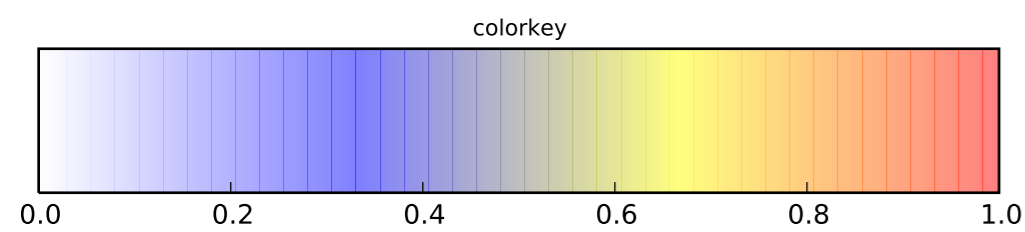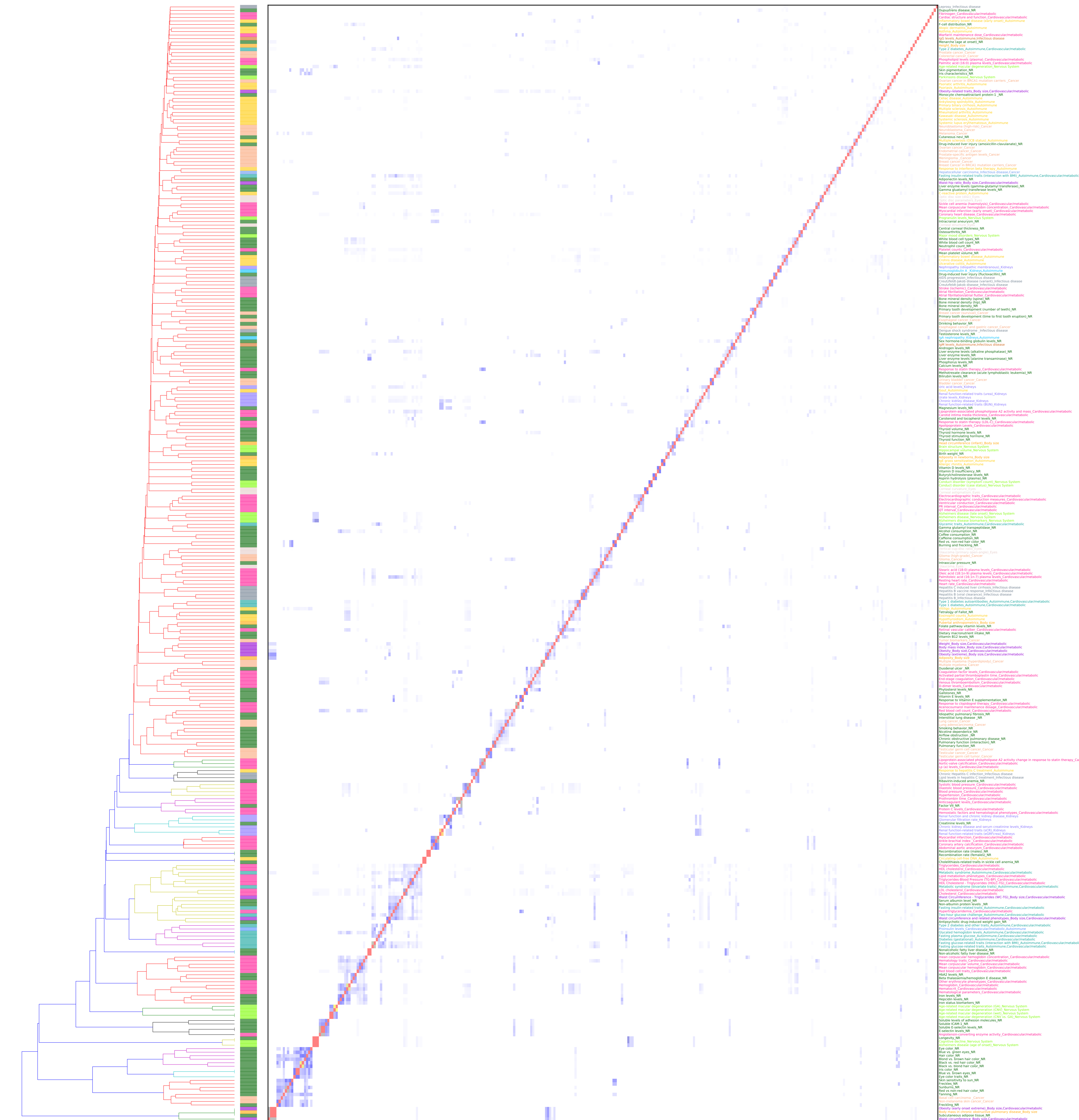

Supplement: Additional file 12: Figure S10. — Hierarchical clustering of NHGRI human traits based on Jaccard index. The hierarchical dendrogram and heat map of similarity for pairwise human traits were constructed based on the Jaccard similarity index, and significance of similarity was measured using a hypergeometric test implemented in the CPAG program. Only traits having at least one significant association (p < 0.05) against other traits are shown here. Colors in the heat map are based on the similarity index and scaled according to the color key. Colored blocks along the y-axis of the heat map and color of text for trait names are indicative of the nine assigned categories of traits. (PDF 75 kb) [file 13059_2015_722_MOESM12_ESM.pdf]

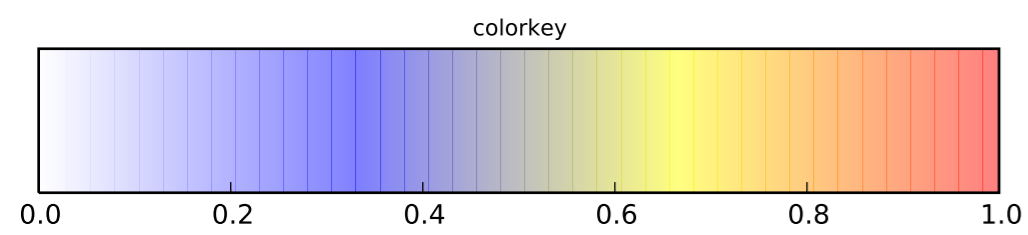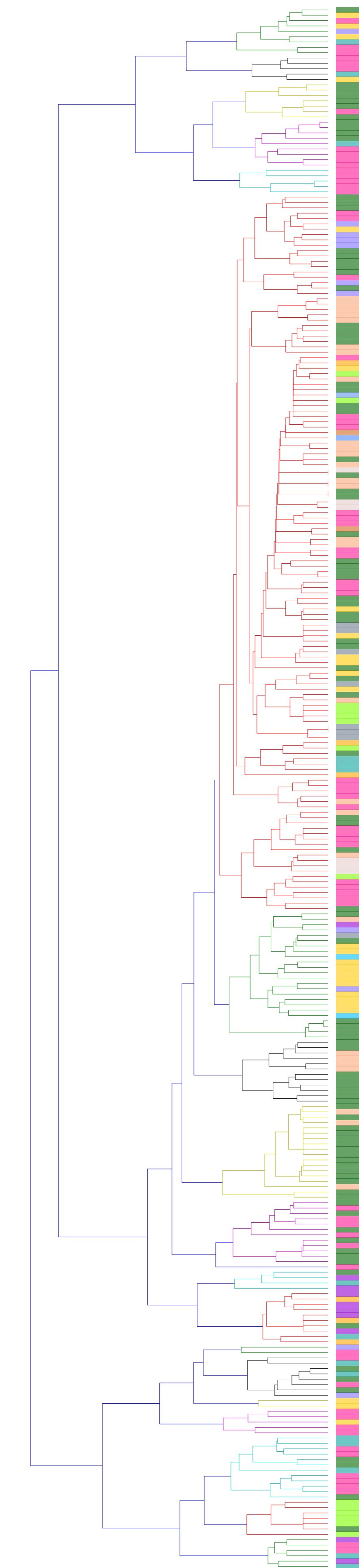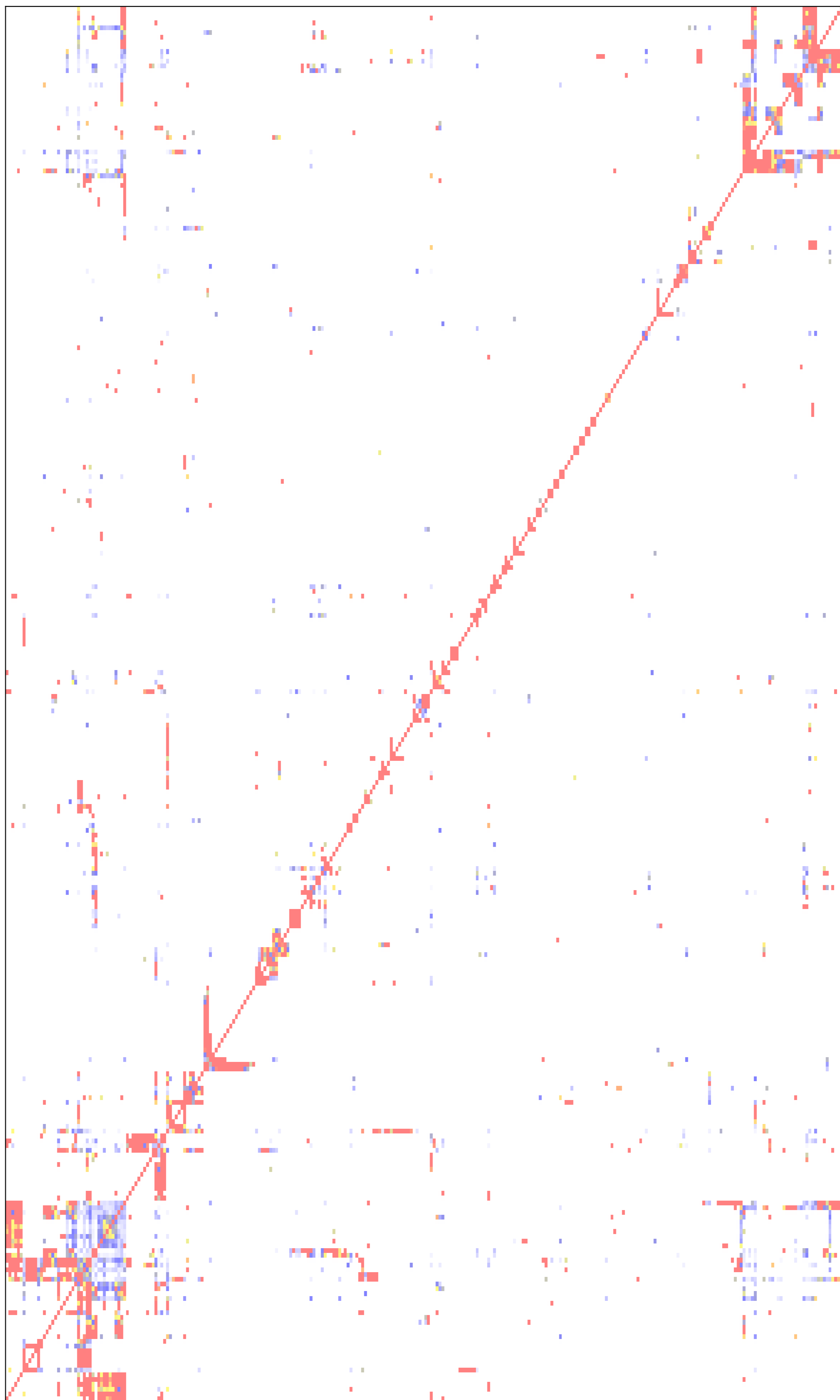[illegible]

Supplement: Additional file 13: Figure S11. — Hierarchical clustering of NHGRI human traits based on Morisita index. The hierarchical dendrogram and heat map of similarity for pairwise human traits were constructed based on the Morisita similarity index, and significance of similarity was measured using a hypergeometric test implemented in the CPAG program. Only traits having at least one significant association (p < 0.05) against other traits are shown here. Colors in the heat map are based on the similarity index and scaled according to the color key. Colored blocks along the y-axis of the heat map and color of text for trait names are indicative of the nine assigned categories of traits. (PDF 70 kb) [file 13059_2015_722_MOESM13_ESM.pdf]

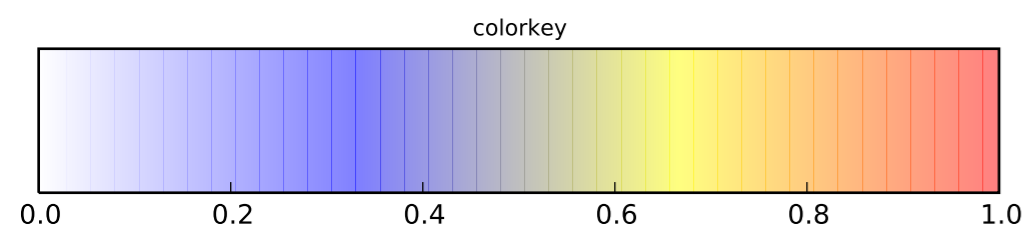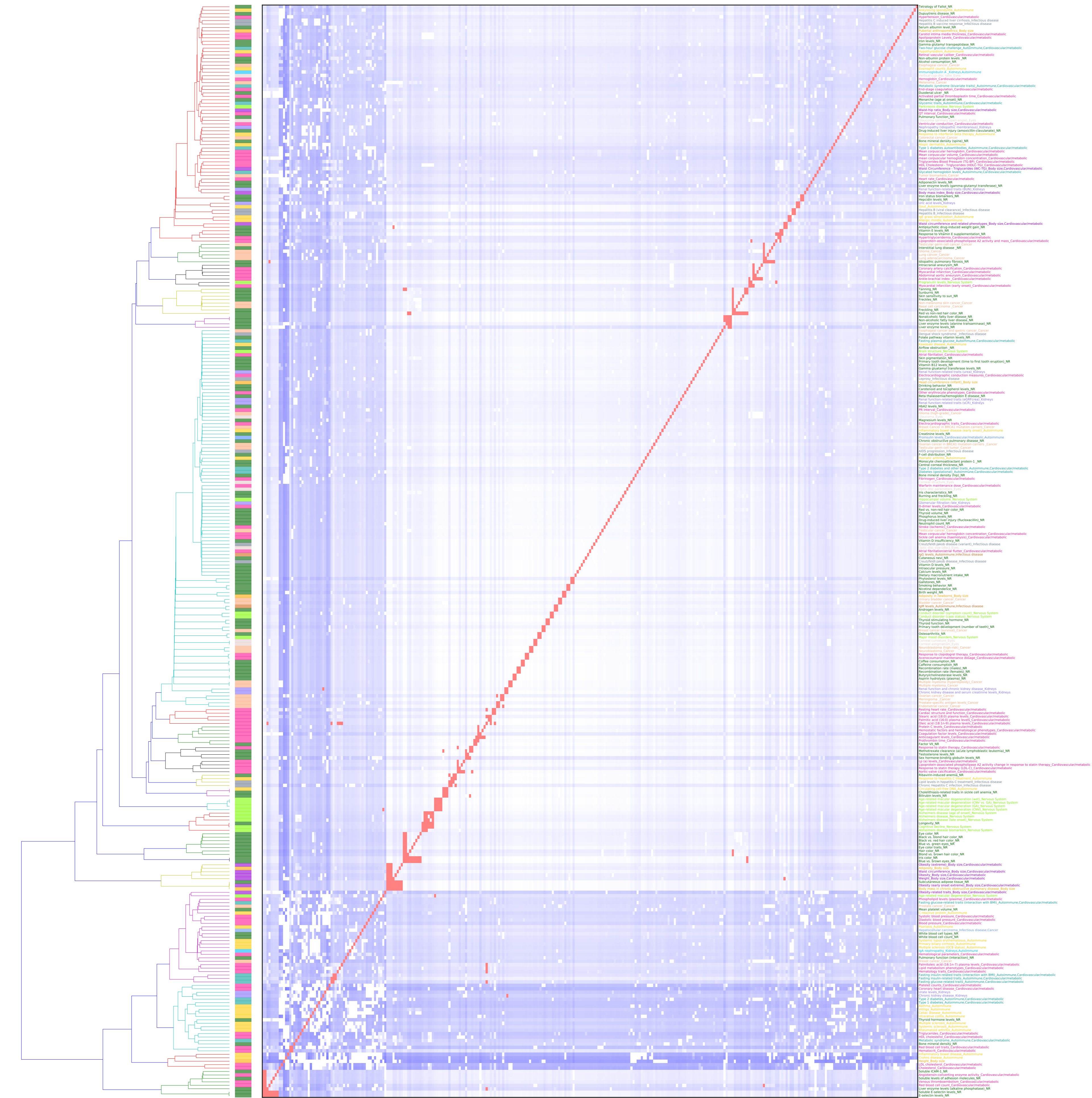

Supplement: Additional file 14: Figure S12. — Hierarchical clustering of NHGRI human traits based on connection specificity index (CSI). The hierarchical dendrogram and heat map of similarity for pairwise human traits were constructed based on the CSI, and significance of similarity was measured using a hypergeometric test implemented in the CPAG program. Only traits having at least one significant association (p < 0.05) against other traits are shown here. Colors in the heat map are based on the similarity index and scaled according to the color key. Colored blocks along the y-axis of the heat map and color of text for trait names are indicative of the nine assigned categories of traits. (PDF 142 kb) [file 13059_2015_722_MOESM14_ESM.pdf]

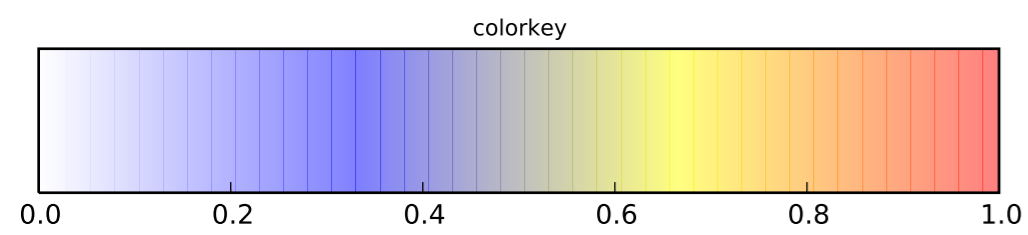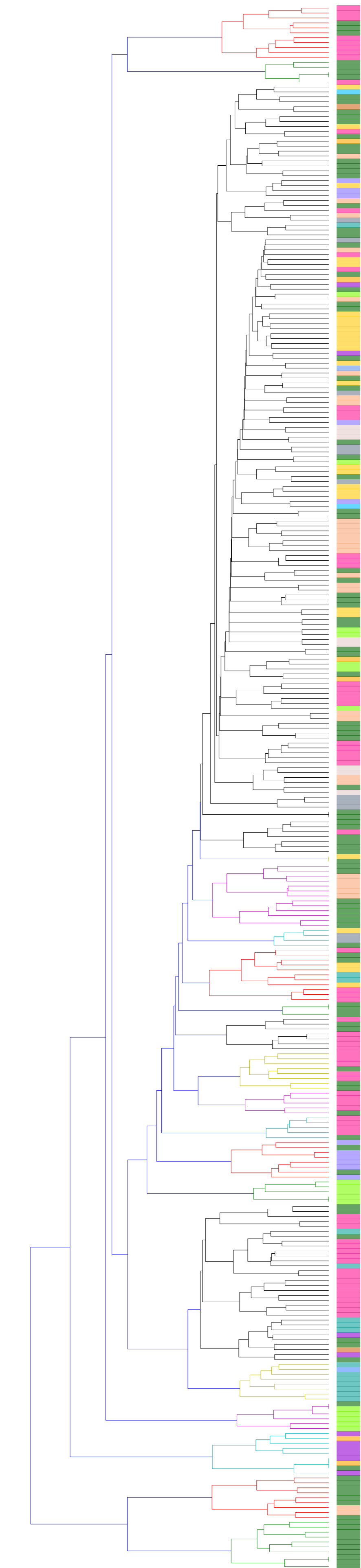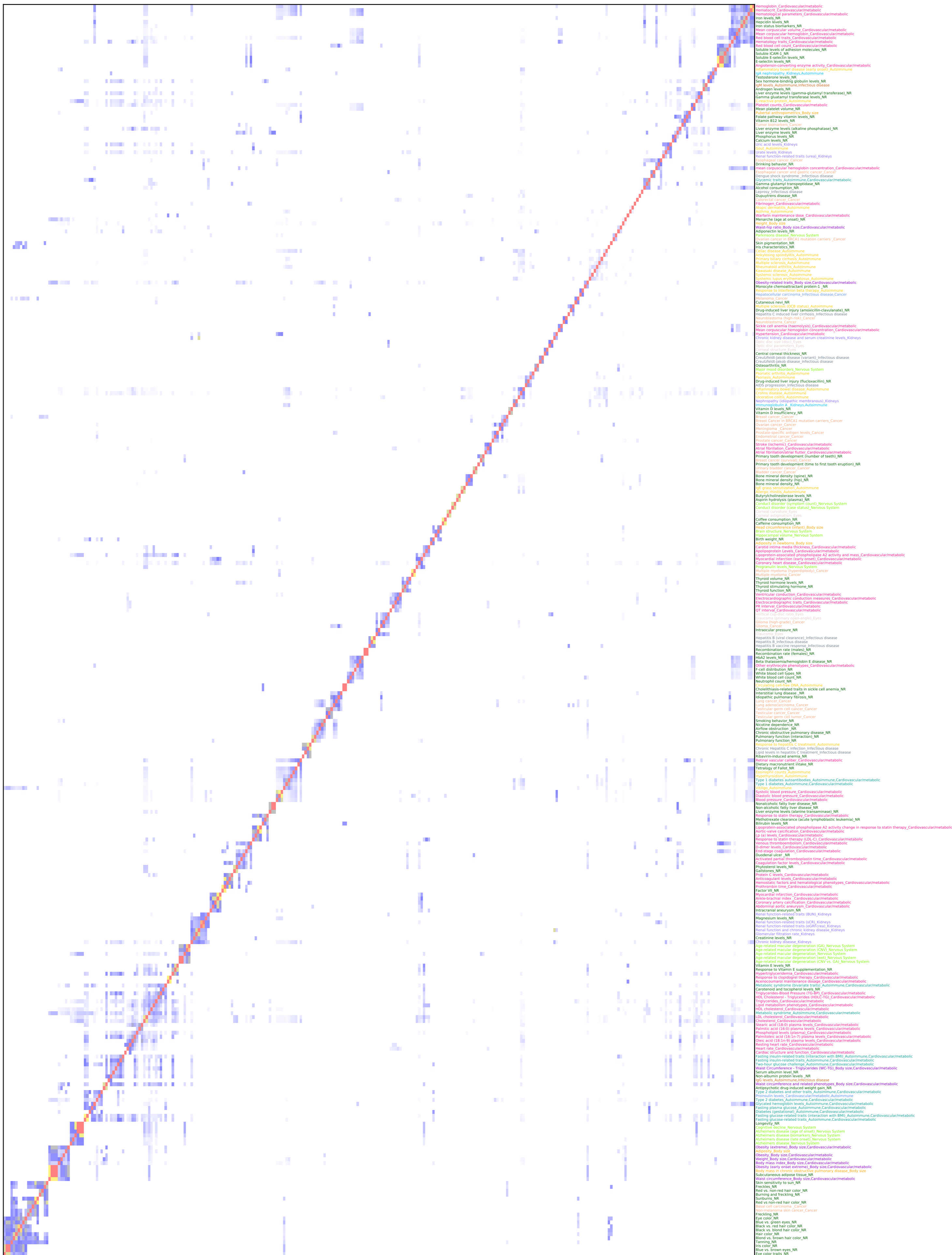

Supplement: Additional file 15: Figure S13. — Hierarchical clustering of NHGRI human traits based on Cosine index. The hierarchical dendrogram and heat map of similarity for pairwise human traits were constructed based on the Cosine similarity index, and significance of similarity was measured using a hypergeometric test implemented in CPAG program. Only traits having at least one significant association (p < 0.05) against other traits are shown here. Colors in the heat map are based on the similarity index and scaled according to the color key. Colored blocks along the y-axis of the heat map and color of text for trait names are indicative of the nine assigned categories of traits. (PDF 77 kb) [file 13059_2015_722_MOESM15_ESM.pdf]

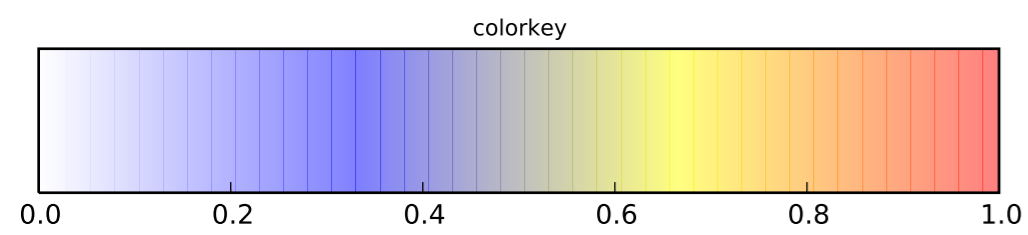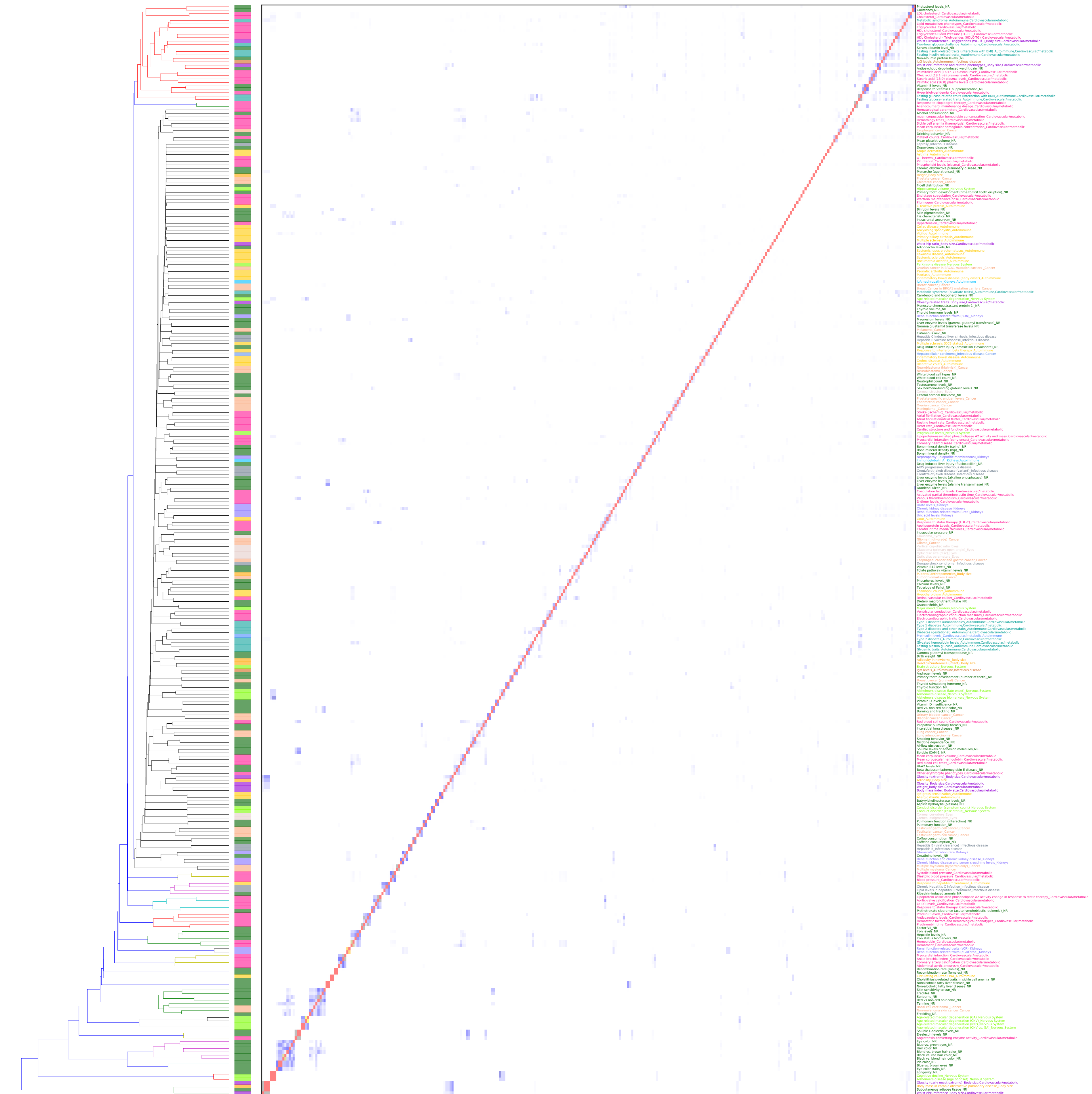

Supplement: Additional file 16: Figure S14. — Hierarchical clustering of NHGRI human traits based on geometric index. The hierarchical dendrogram and heat map of similarity for pairwise human traits were constructed based on the geometric similarity index, and significance of similarity was measured using a hypergeometric test implemented in the CPAG program. Only traits having at least one significant association (p < 0.05) against other traits are shown here. Colors in the heat map are based on the similarity index and scaled according to the color key. Colored blocks along the y-axis of the heat map and color of text for trait names are indicative of the nine assigned categories of traits. (PDF 73 kb) [file 13059_2015_722_MOESM16_ESM.pdf]

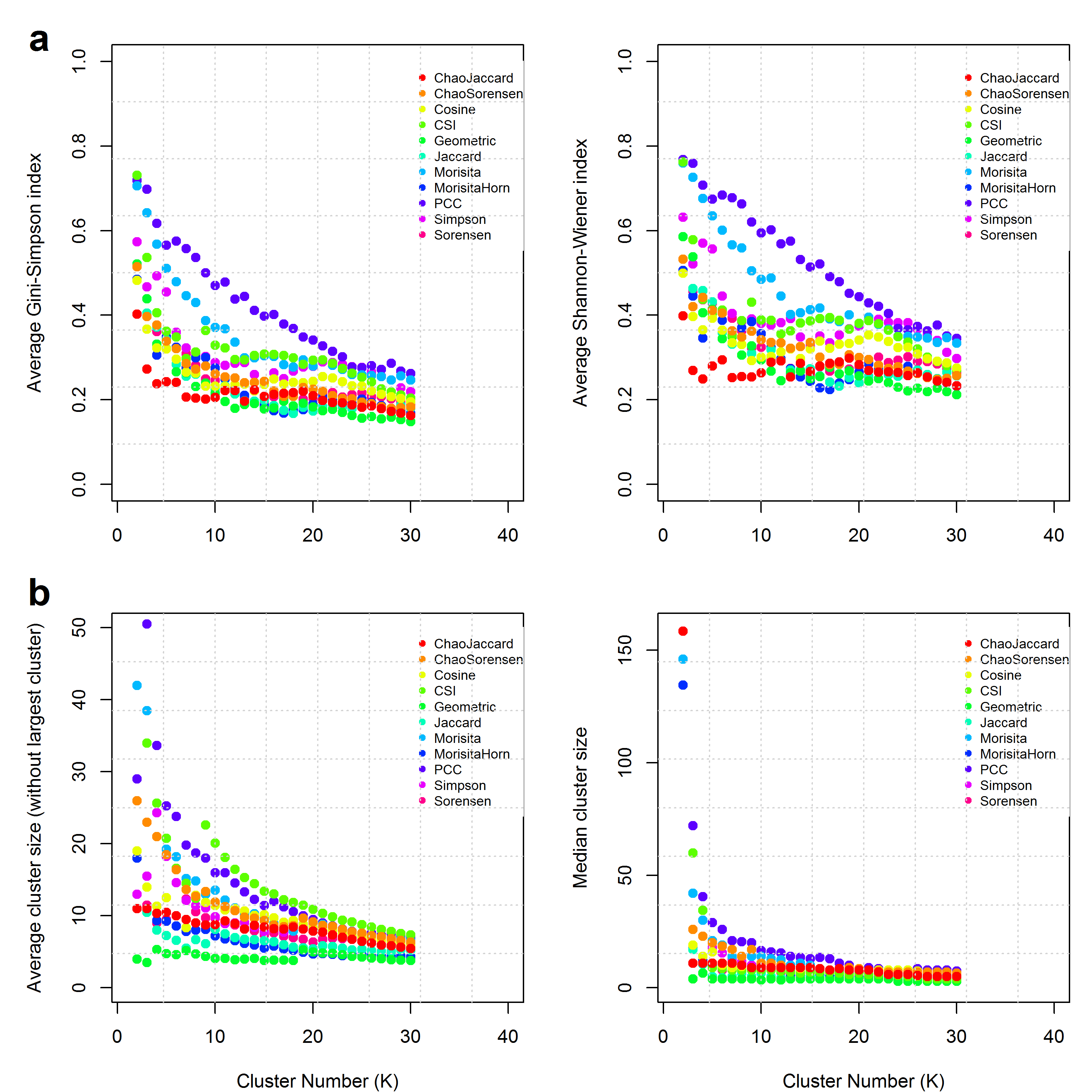

Supplement: Additional file 17: Figure S15. — Entropy-based comparisons of 11 similarity indices. a) Average Gini-Simpson entropy index and Shannon-Wiener entropy index were calculated for each cluster number K for each similarity index. Both entropy indices are unweighted without considering effect of cluster size. The Chao-Sorensen similarity index had the least heterogeneity across different K for both entropy indices. b) Average and median cluster size were plotted against cluster number K. For Chao-Sorensen, the cluster sizes have slight variation while K increases from 2 to 30, and also generates medium to large cluster. We removed the largest cluster of the hierarchical clustering tree from this analysis to reduce possible bias to the average cluster size. (TIFF 346 kb) [file 13059_2015_722_MOESM17_ESM.tiff]
