# Supplementary material for: CPAG: software for leveraging pleiotropy in GWAS to reveal similarity between human traits links plasma fatty acids and intestinal inflammation
Source: Genome Biol. 2015 Sep 15;16(1):190. doi: 10.1186/s13059-015-0722-1 (PMC4570686; doi:10.1186/s13059-015-0722-1)
Supplement: Additional file 18: Figure S16. — Density distributions of non-zero similarity values for inter-group pairwise comparisons and for pairwise comparisons within predefined groups. Pairwise comparisons within seven pre-defined groups generally show higher similarity than pairwise comparisons of traits between the pre-defined groups (inter-group). (PDF 25 kb) [file 13059_2015_722_MOESM18_ESM.pdf]

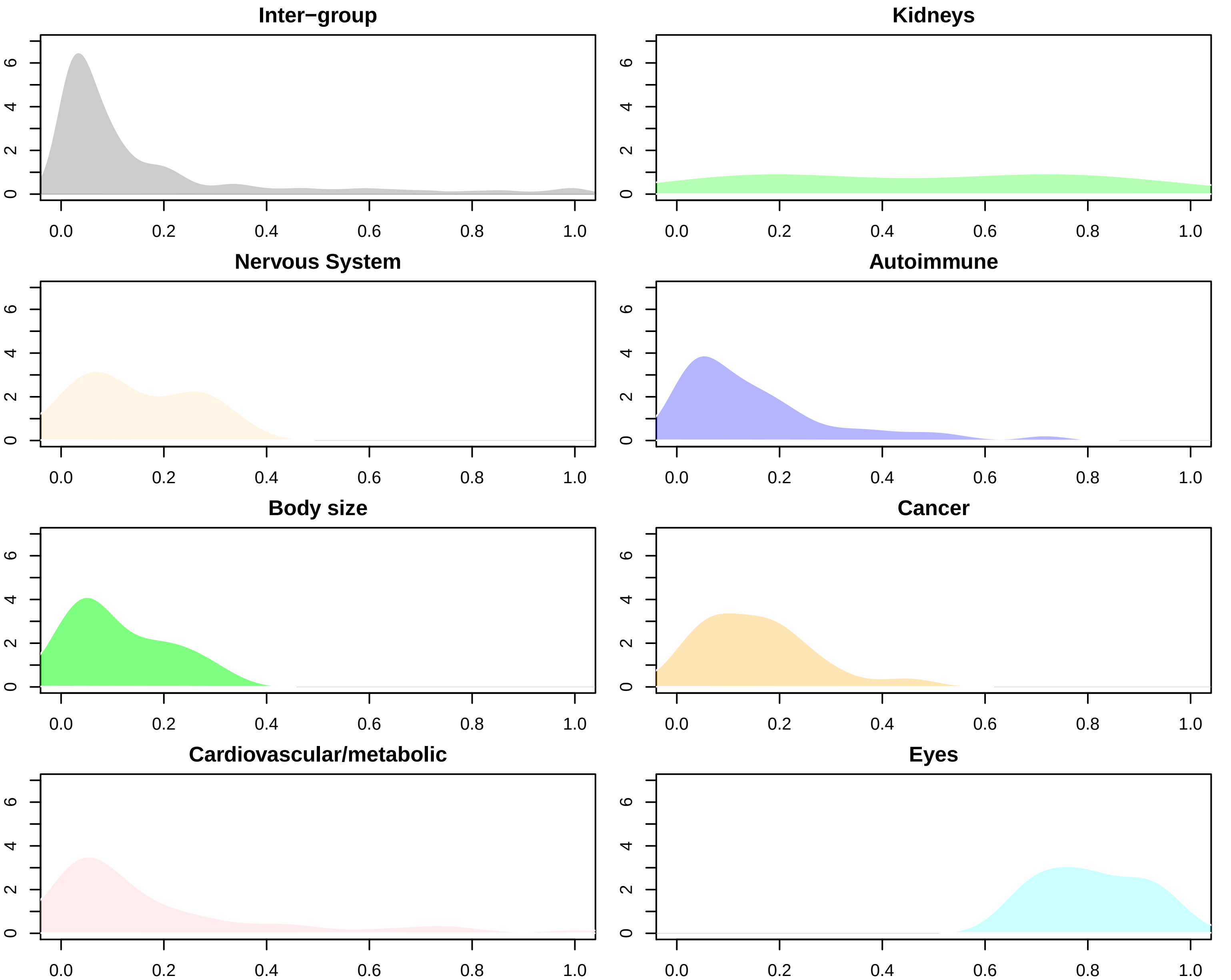

Inter−group
Kidneys
Nervous System
Autoimmune
Body size
Cancer
Cardiovascular/metabolic
Eyes
0
2
4
6
0.0
0.2
0.4
0.6
0.8
1.0
