# Supplementary material for: CPAG: software for leveraging pleiotropy in GWAS to reveal similarity between human traits links plasma fatty acids and intestinal inflammation
Source: Genome Biol. 2015 Sep 15;16(1):190. doi: 10.1186/s13059-015-0722-1 (PMC4570686; doi:10.1186/s13059-015-0722-1)
Supplement: Additional file 19: Table S3. — Potentially novel raw trait pairs revealed by CPAG and a lack of any co-occurrences in PubMed (last visit on 20 March 2015). The two raw traits with significant similarity (p < 0.05, Fisher’s exact test after Bonferroni correction) are listed as “Trait1” and “Trait2”. The text was modified to remove general terms such as “levels” to broaden the PubMed query (see "Materials and methods"). The number of PubMed hits for each individual trait is given. Out of 741 raw trait pairs with p < 0.05, these 43 had no PubMed co-occurrences. The trait pair we tested experimentally in zebrafish (Crohn’s disease and palmitoleic acid plasma levels) is highlighted in orange. (DOCX 20 kb) [file 13059_2015_722_MOESM19_ESM.docx]

**Table S3. Potentially novel raw trait pairs revealed by CPAG and a lack of any co-occurrences in PubMed.** The link between Crohn’s disease and palmitoleic acid (highlighted in yellow) was examined experimentally in a zebrafish model of intestinal inflammation, as described in the text.

| Trait 1 | Trait 2 | PubMed Query Trait1 | PubMed Query Trait2 | Trait1 Hits | Trait 2 Hits |
| --- | --- | --- | --- | --- | --- |
| AIDS progression | Psoriasis | AIDS progression | Psoriasis | 371 | 28096 |
| AIDS progression | Drug-induced liver injury (flucloxacillin) | AIDS progression | Drug-induced liver injury | 371 | 1093 |
| Angiotensin-converting enzyme activity | Soluble E-selectin levels | Angiotensin-converting enzyme activity | Soluble E-selectin | 969 | 686 |
| Angiotensin-converting enzyme activity | Soluble ICAM-1 | Angiotensin-converting enzyme activity | Soluble ICAM-1 | 969 | 596 |
| Ankle-brachial index | Intracranial aneurysm | Ankle-brachial index | Intracranial aneurysm | 3019 | 2833 |
| Beta thalassemia/hemoglobin E disease | F-cell distribution | Beta thalassemia/hemoglobin E disease | F-cell distribution | 26 | 3 |
| Bilirubin levels | Methotrexate clearance (acute lymphoblastic leukemia) | Bilirubin | Methotrexate clearance | 28314 | 55 |
| Bilirubin levels | Circulating cell-free DNA | Bilirubin | Circulating cell-free DNA | 28314 | 152 |
| Celiac disease | Type 1 diabetes autoantibodies | Celiac disease | Type 1 diabetes autoantibodies | 8437 | 6 |
| Chronic Hepatitis C infection | Ribavirin-induced anemia | Chronic Hepatitis C infection | Ribavirin-induced anemia | 1014 | 39 |
| Coagulation factor levels | End-stage coagulation | Coagulation factor | End-stage coagulation | 5740 | 3 |
| Coronary heart disease | End-stage coagulation | Coronary heart disease | End-stage coagulation | 40879 | 3 |
| Crohns disease | Type 1 diabetes autoantibodies | Crohn's disease | Type 1 diabetes autoantibodies | 30593 | 6 |
| Crohns disease | Palmitoleic acid (16:1n-7) plasma levels | Crohn's disease | Palmitoleic acid | 30593 | 742 |
| Dietary macronutrient intake | Retinal vascular caliber | Dietary macronutrient intake | Retinal vascular caliber | 46 | 125 |
| Duodenal ulcer | Phytosterol levels | Duodenal ulcer | Phytosterol | 15192 | 729 |
| Dupuytrens disease | Inflammatory bowel disease | Dupuytrens disease | Inflammatory bowel disease | 6 | 26108 |
| End-stage coagulation | Venous thromboembolism | End-stage coagulation | Venous thromboembolism | 3 | 12420 |
| Eosinophil counts | Tetralogy of Fallot | Eosinophil counts | Tetralogy of Fallot | 1957 | 6799 |
| E-selectin levels | Red blood cell count | E-selectin | Red blood cell count | 6308 | 1242 |
| Glycated hemoglobin levels | Iron status biomarkers | Glycated hemoglobin | Iron status | 3617 | 4018 |
| Hepatitis B vaccine response | Hepatitis C induced liver cirrhosis | Hepatitis B vaccine response | Hepatitis C induced liver cirrhosis | 17 | 10 |
| Hypothyroidism | Type 1 diabetes autoantibodies | Hypothyroidism | Type 1 diabetes autoantibodies | 24152 | 6 |
| Idiopathic pulmonary fibrosis | Testicular germ cell cancer | Idiopathic pulmonary fibrosis | Testicular germ cell cancer | 4344 | 244 |
| Idiopathic pulmonary fibrosis | Red blood cell count | Idiopathic pulmonary fibrosis | Red blood cell count | 4344 | 1242 |
| Inflammatory bowel disease | Type 1 diabetes autoantibodies | Inflammatory bowel disease | Type 1 diabetes autoantibodies | 26108 | 6 |
| Iris color | Tanning | Iris color | Tanning | 234 | 2043 |
| Liver enzyme levels (alkaline phosphatase) | Soluble E-selectin levels | Liver enzyme | Soluble E-selectin | 4367 | 686 |
| Magnesium levels | Renal function-related traits (sCR) | Magnesium | Renal function-related | 45698 | 37 |
| Magnesium levels | Renal function-related traits (eGRFcrea) | Magnesium | Renal function-related | 45698 | 37 |
| Magnesium levels | Renal function-related traits (BUN) | Magnesium | Renal function-related | 45698 | 37 |
| Major mood disorders | Osteoarthritis | Major mood disorders | Osteoarthritis | 130 | 38703 |
| Myocardial infarction (early onset) | Progranulin levels | Myocardial infarction | Progranulin | 137664 | 588 |
| Oleic acid (18:1n-9) plasma levels | Resting heart rate | Oleic acid | Resting heart rate | 11206 | 2705 |
| Palmitoleic acid (16:1n-7) plasma levels | Platelet counts | Palmitoleic acid | Platelet counts | 742 | 9217 |
| Platelet counts | Sex hormone-binding globulin levels | Platelet counts | Sex hormone-binding globulin | 9217 | 5051 |
| Red blood cell count | Soluble E-selectin levels | Red blood cell count | Soluble E-selectin | 1242 | 686 |
| Renal function-related traits (urea) | Urate levels | Renal function-related | Urate | 37 | 5890 |
| Retinal vascular caliber | Tetralogy of Fallot | Retinal vascular caliber | Tetralogy of Fallot | 125 | 6799 |
| Rheumatoid arthritis | Type 1 diabetes autoantibodies | Rheumatoid arthritis | Type 1 diabetes autoantibodies | 81554 | 6 |
| Soluble E-selectin levels | Venous thromboembolism | Soluble E-selectin | Venous thromboembolism | 686 | 12420 |
| Type 1 diabetes autoantibodies | Vitiligo | Type 1 diabetes autoantibodies | Vitiligo | 6 | 4867 |
| White blood cell count | White blood cell types | White blood cell count | White blood cell types | 7455 | 23 |
