# Supplementary material for: CPAG: software for leveraging pleiotropy in GWAS to reveal similarity between human traits links plasma fatty acids and intestinal inflammation
Source: Genome Biol. 2015 Sep 15;16(1):190. doi: 10.1186/s13059-015-0722-1 (PMC4570686; doi:10.1186/s13059-015-0722-1)
Supplement: Additional file 20: Table S4. — Potentially novel modified trait pairs revealed by CPAG and a lack of any co-occurrences in PubMed (last visit on 20 March 2015). The two modified traits with significant similarity (p < 0.05, Fisher’s exact test after Bonferroni correction) are listed as “Trait1” and “Trait2”. The text was modified to remove general terms such as “levels” to broaden the PubMed query (see "Materials and methods"). The number of PubMed hits for each individual trait is given. Out of 277 modified trait pairs with p < 0.05, these 30 had no PubMed co-occurrences. (DOCX 19 kb) [file 13059_2015_722_MOESM20_ESM.docx]

**Table S4. Potentially novel modified trait pairs revealed by CPAG and a lack of any co-occurrences in PubMed.**

| Trait1 | Trait2 | PubMed Query Trait1 | PubMed Query Trait2 | Trait1 Hits | Trait 2 Hits |
| --- | --- | --- | --- | --- | --- |
| Abdominal aortic aneurysm | Migraine | Abdominal aortic aneurysm | Migraine | 10210 | 25137 |
| Alcohol associated | Liver enzyme levels | Alcohol associated | Liver enzyme | 492 | 4367 |
| Angiotensin-converting enzyme activity | Soluble E-selectin levels | Angiotensin-converting enzyme activity | Soluble E-selectin | 969 | 686 |
| Angiotensin-converting enzyme activity | Soluble ICAM-1 | Angiotensin-converting enzyme activity | Soluble ICAM-1 | 969 | 596 |
| Ankle-brachial index | Intracranial aneurysm | Ankle-brachial index | Intracranial aneurysm | 3019 | 2833 |
| Beta thalassemia/hemoglobin E disease | F-cell distribution | Beta thalassemia/hemoglobin E disease | F-cell distribution | 26 | 3 |
| Beta-2 microglubulin plasma levels | Tetralogy of Fallot | Beta-2 microglubulin | Tetralogy of Fallot | 10 | 6799 |
| Beta-2 microglubulin plasma levels | Retinal vascular caliber | Beta-2 microglubulin | Retinal vascular caliber | 10 | 125 |
| Beta-trace protein levels | Corneal structure | Beta-trace protein | Corneal structure | 193 | 130 |
| Bilirubin levels | Methotrexate clearance (acute lymphoblastic leukemia) | Bilirubin | Methotrexate clearance | 28314 | 55 |
| Bilirubin levels | Circulating cell-free DNA | Bilirubin | Circulating cell-free DNA | 28314 | 152 |
| Blood vessel measurements | Drinking behavior | Blood vessel | Drinking behavior | 14305 | 2463 |
| Chronic kidney disease | Rhegmatogenous retinal detachment | Chronic kidney disease | Rhegmatogenous retinal detachment | 22341 | 1739 |
| Dehydroepiandrosterone sulphate levels | Ewing sarcoma | Dehydroepiandrosterone sulphate | Ewing sarcoma | 1060 | 1958 |
| Dietary macronutrient intake | Retinal vascular caliber | Dietary macronutrient intake | Retinal vascular caliber | 46 | 125 |
| Duodenal ulcer | Phytosterol levels | Duodenal ulcer | Phytosterol | 15192 | 729 |
| Dupuytrens disease | Inflammatory bowel disease | Dupuytrens disease | Inflammatory bowel disease | 6 | 26108 |
| Eosinophil counts | Tetralogy of Fallot | Eosinophil counts | Tetralogy of Fallot | 1957 | 6799 |
| Eosinophil counts | Retinal vascular caliber | Eosinophil counts | Retinal vascular caliber | 1957 | 125 |
| HPV seropositivity | Primary biliary cirrhosis | HPV seropositivity | Primary biliary cirrhosis | 56 | 6972 |
| Hypothyroidism | Retinal vascular caliber | Hypothyroidism | Retinal vascular caliber | 24152 | 125 |
| Idiopathic pulmonary fibrosis | Testicular cancer | Idiopathic pulmonary fibrosis | Testicular cancer | 4344 | 4609 |
| Ig Levels | Non-albumin protein levels | Ig | Non-albumin protein | 30526 | 9 |
| Methotrexate clearance (acute lymphoblastic leukemia) | Sex hormone-binding globulin levels | Methotrexate clearance | Sex hormone-binding globulin | 55 | 5051 |
| Myocardial infarction | Progranulin levels | Myocardial infarction | Progranulin | 137664 | 588 |
| N-glycan levels | Vitamin B12 levels | N-glycan | Vitamin B12 | 2079 | 13276 |
| Pancreatic cancer | Phytosterol levels | Pancreatic cancer | Phytosterol | 22706 | 729 |
| Retinal vascular caliber | Tetralogy of Fallot | Retinal vascular caliber | Tetralogy of Fallot | 125 | 6799 |
| Serum urate | Thyroid hormone levels | Serum urate | Thyroid hormone | 780 | 21504 |
| Vitamin E levels | Warfarin maintenance dose | Vitamin E | Warfarin maintenance dose | 23450 | 107 |
