# Supplementary material for: CPAG: software for leveraging pleiotropy in GWAS to reveal similarity between human traits links plasma fatty acids and intestinal inflammation
Source: Genome Biol. 2015 Sep 15;16(1):190. doi: 10.1186/s13059-015-0722-1 (PMC4570686; doi:10.1186/s13059-015-0722-1)
Supplement: Additional file 21: Table S5. — NHGRI raw trait names, modified names and their pre-defined groups. NHGRI disease names (“Raw traits”) are from the NHGRI GWAS Catalog. Closely related phenotypes were merged and phenotypes in the NHGRI GWAS Catalog that combined multiple diseases were removed for “modified phenotypes”. Each trait was assigned to one or two broad categories (autoimmune, infectious disease, cardiovascular/metabolic, body size, eyes, kidneys, nervous system, cancer, or other) based on medical knowledge of the authors prior to running the CPAG analysis. (DOCX 104 kb) [file 13059_2015_722_MOESM21_ESM.docx]

**Table S5. NHGRI GWAS Catalog raw trait names, modified trait names (merging closely related traits), and their assignment to predefined groups for inter-group vs. intra-group similarity analyses.** NA = not assigned.

| Raw Trait Name | Modified Name | Disease group |
| --- | --- | --- |
| Adiposity in newborns | Adiposity | Body size |
| Age-related macular degeneration | Age-related macular degeneration | Nervous System |
| Age-related macular degeneration (CNV vs. GA) | Age-related macular degeneration | Nervous System |
| Age-related macular degeneration (CNV) | Age-related macular degeneration | Nervous System |
| Age-related macular degeneration (GA) | Age-related macular degeneration | Nervous System |
| Age-related macular degeneration (wet) | Age-related macular degeneration | Nervous System |
| Aging | Aging | NA |
| Aging (facial) | Aging | NA |
| Aging (time to death) | Aging | NA |
| Aging (time to event) | Aging | NA |
| Aging traits | Aging | NA |
| Alcohol and nictotine co-dependence | Alcohol associated | NA |
| Alcohol consumption | Alcohol associated | NA |
| Alcohol dependence | Alcohol associated | NA |
| Alcoholism (12-month weekly alcohol consumption) | Alcohol associated | NA |
| Alcoholism (alcohol dependence factor score) | Alcohol associated | NA |
| Alcoholism (alcohol use disorder factor score) | Alcohol associated | NA |
| Alcoholism (heaviness of drinking) | Alcohol associated | NA |
| Alzheimers disease | Alzheimers disease | Nervous System |
| Alzheimers disease (age of onset) | Alzheimers disease | Nervous System |
| Alzheimers disease (cognitive decline) | Alzheimers disease | Nervous System |
| Alzheimers disease (late onset) | Alzheimers disease | Nervous System |
| Alzheimers disease biomarkers | Alzheimers disease | Nervous System |
| Response to cholinesterase inhibitors in Alzheimers disease | Alzheimers disease | Nervous System |
| Amyotrophic lateral sclerosis | Amyotrophic lateral sclerosis | Nervous System |
| Amyotrophic lateral sclerosis (age of onset) | Amyotrophic lateral sclerosis | Nervous System |
| Aortic root size | Aortic | Cardiovascular |
| Aortic stiffness | Aortic | Cardiovascular |
| Aortic-valve calcification | Aortic | Cardiovascular |
| Asthma | Asthma | Autoimmune |
| Asthma (bronchodilator response) | Asthma | Autoimmune |
| Asthma (childhood onset) | Asthma | Autoimmune |
| Asthma (toluene diisocyanate-induced) | Asthma | Autoimmune |
| Attention deficit hyperactivity disorder | Attention deficit hyperactivity disorder | Nervous System |
| Attention deficit hyperactivity disorder (time to onset) | Attention deficit hyperactivity disorder | Nervous System |
| Attention deficit hyperactivity disorder and conduct disorder | Attention deficit hyperactivity disorder | Nervous System |
| Attention deficit hyperactivity disorder motor coordination | Attention deficit hyperactivity disorder | Nervous System |
| Attention deficit hyperactivity disorder symptoms (interaction) | Attention deficit hyperactivity disorder | Nervous System |
| Bipolar disorder | Bipolar disorder | Nervous System |
| Bipolar disorder (age of onset and psychomotor symptoms) | Bipolar disorder | Nervous System |
| Bipolar disorder (mania) | Bipolar disorder | Nervous System |
| Bipolar disorder (mood-incongruent) | Bipolar disorder | Nervous System |
| Bipolar disorder and major depressive disorder (combined) | Bipolar disorder | Nervous System |
| Bipolar disorder and schizophrenia | Bipolar disorder | Nervous System |
| Body mass (lean) | Body mass index | Body size |
| Body mass in chronic obstructive pulmonary disease | Body mass index | Body size |
| Body mass index | Body mass index | Body size |
| Body mass index (interaction) | Body mass index | Body size |
| Body mass index and cholesterol (psychopharmacological treatment) | Body mass index | Body size |
| Bone mineral density | Bone mineral density | NA |
| Bone mineral density (hip) | Bone mineral density | NA |
| Bone mineral density (spine) | Bone mineral density | NA |
| Breast cancer | Breast cancer | Cancer |
| Breast cancer (male) | Breast cancer | Cancer |
| Breast cancer (prognosis) | Breast cancer | Cancer |
| Breast cancer (survival) | Breast cancer | Cancer |
| Breast Cancer in BRCA1 mutation carriers | Breast cancer | Cancer |
| Cardiac hypertrophy | Cardiac | Cardiovascular |
| Cardiac repolarization | Cardiac | Cardiovascular |
| Cardiac structure and function | Cardiac | Cardiovascular |
| Cardiac Troponin-T levels | Cardiac | Cardiovascular |
| Left ventricular mass | Cardiac | Cardiovascular |
| Total ventricular volume | Cardiac | Cardiovascular |
| Celiac disease | Celiac disease | Autoimmune |
| Celiac disease and Rheumatoid arthritis | Celiac disease | Autoimmune |
| Cholesterol | Cholesterol | Cardiovascular |
| Cholesterol and Triglycerides | Cholesterol | Cardiovascular |
| Cholesterol, total | Cholesterol | Cardiovascular |
| HDL cholesterol | Cholesterol | Cardiovascular |
| HDL Cholesterol - Triglycerides (HDLC-TG) | Cholesterol | Cardiovascular |
| Hypertriglyceridemia | Cholesterol | Cardiovascular |
| LDL (oxidized) | Cholesterol | Cardiovascular |
| LDL cholesterol | Cholesterol | Cardiovascular |
| Lipid metabolism phenotypes | Cholesterol | Cardiovascular |
| Lipid traits | Cholesterol | Cardiovascular |
| Lipoprotein-associated phospholipase A2 activity and mass | Cholesterol | Cardiovascular |
| Lipoprotein-associated phospholipase A2 activity change in response to statin therapy | Cholesterol | Cardiovascular |
| Response to fenofibrate | Cholesterol | Cardiovascular |
| Response to fenofibrate (adiponectin levels) | Cholesterol | Cardiovascular |
| Response to statin therapy | Cholesterol | Cardiovascular |
| Response to statin therapy (LDL-C) | Cholesterol | Cardiovascular |
| Triglycerides | Cholesterol | Cardiovascular |
| Triglycerides-Blood Pressure (TG-BP) | Cholesterol | Cardiovascular |
| Chronic obstructive pulmonary disease | Chronic obstructive pulmonary disease | NA |
| Chronic obstructive pulmonary disease-related biomarkers | Chronic obstructive pulmonary disease | NA |
| Cognitive decline | Cognitive | Nervous System |
| Cognitive function | Cognitive | Nervous System |
| Cognitive performance | Cognitive | Nervous System |
| Cognitive test performance | Cognitive | Nervous System |
| Conduct disorder (case status) | Conduct disorder | Nervous System |
| Conduct disorder (interaction) | Conduct disorder | Nervous System |
| Conduct disorder (symptom count) | Conduct disorder | Nervous System |
| Coronary artery calcification | Coronary disease | Cardiovascular |
| Coronary heart disease | Coronary disease | Cardiovascular |
| Coronary restenosis | Coronary disease | Cardiovascular |
| Coronary spasm | Coronary disease | Cardiovascular |
| Creutzfeldt-Jakob disease | Creutzfeldt-Jakob diseaes | Infectious disease |
| Creutzfeldt-Jakob disease (variant) | Creutzfeldt-Jakob diseaes | Infectious disease |
| Metabolite levels (5-HIAA) | CSF metabolites | Nervous System |
| Metabolite levels (5-HIAA/ MHPG Ratio) | CSF metabolites | Nervous System |
| Metabolite levels (HVA) | CSF metabolites | Nervous System |
| Metabolite levels (HVA/5-HIAA ratio) | CSF metabolites | Nervous System |
| Metabolite levels (HVA/MHPG ratio) | CSF metabolites | Nervous System |
| Metabolite levels (HVA-5-HIAA Factor score) | CSF metabolites | Nervous System |
| Metabolite levels (MHPG) | CSF metabolites | Nervous System |
| Depression (quantitative trait) | Depression | Nervous System |
| Depression and alcohol dependence | Depression | Nervous System |
| Major depressive disorder | Depression | Nervous System |
| Major depressive disorder (broad) | Depression | Nervous System |
| Major mood disorders | Depression | Nervous System |
| Response to antidepressant treatment | Depression | Nervous System |
| Response to antidepressants | Depression | Nervous System |
| Diabetes (gestational) | Diabetes | Autoimmune,Cardiovascular |
| Diabetes (incident) | Diabetes | Autoimmune,Cardiovascular |
| Diabetes related insulin traits | Diabetes | Autoimmune,Cardiovascular |
| Diabetic retinopathy | Diabetes | Autoimmune,Cardiovascular |
| Fasting glucose-related traits | Diabetes | Autoimmune,Cardiovascular |
| Fasting glucose-related traits (interaction with BMI) | Diabetes | Autoimmune,Cardiovascular |
| Fasting insulin-related traits | Diabetes | Autoimmune,Cardiovascular |
| Fasting insulin-related traits (interaction with BMI) | Diabetes | Autoimmune,Cardiovascular |
| Fasting plasma glucose | Diabetes | Autoimmune,Cardiovascular |
| Glycated hemoglobin levels | Diabetes | Autoimmune,Cardiovascular |
| Glycemic traits | Diabetes | Autoimmune,Cardiovascular |
| Insulin resistance/response | Diabetes | Autoimmune,Cardiovascular |
| Insulin-related traits | Diabetes | Autoimmune,Cardiovascular |
| Metabolic syndrome | Diabetes | Autoimmune,Cardiovascular |
| Metabolic syndrome (bivariate traits) | Diabetes | Autoimmune,Cardiovascular |
| Proinsulin levels | Diabetes | Cardiovascular,Autoimmune |
| Response to metformin | Diabetes | Autoimmune,Cardiovascular |
| Two-hour glucose challenge | Diabetes | Autoimmune,Cardiovascular |
| Type 1 diabetes | Diabetes | Autoimmune,Cardiovascular |
| Type 1 diabetes autoantibodies | Diabetes | Autoimmune,Cardiovascular |
| Type 1 diabetes nephropathy | Diabetes | Autoimmune,Cardiovascular |
| Type 2 diabetes | Diabetes | Autoimmune,Cardiovascular |
| Type 2 diabetes (dietary heme iron intake interaction) | Diabetes | Autoimmune,Cardiovascular |
| Type 2 diabetes and other traits | Diabetes | Autoimmune,Cardiovascular |
| Type 2 diabetes nephropathy | Diabetes | Autoimmune,Cardiovascular |
| Drug-induced liver injury | Drug-induced liver injury | NA |
| Drug-induced liver injury (amoxicillin-clavulanate) | Drug-induced liver injury | NA |
| Drug-induced liver injury (flucloxacillin) | Drug-induced liver injury | NA |
| Eating disorders | Eating disorders | Nervous System |
| Eating disorders (purging via substances) | Eating disorders | Nervous System |
| Antipsychotic-induced QTc interval prolongation | ECG parameters | Cardiovascular |
| Atrial fibrillation | ECG parameters | Cardiovascular |
| Atrial fibrillation/atrial flutter | ECG parameters | Cardiovascular |
| Atrioventricular conduction | ECG parameters | Cardiovascular |
| Electrocardiographic conduction measures | ECG parameters | Cardiovascular |
| Electrocardiographic traits | ECG parameters | Cardiovascular |
| Heart rate | ECG parameters | Cardiovascular |
| Heart rate variability traits | ECG parameters | Cardiovascular |
| Life threatening arrhythmia | ECG parameters | Cardiovascular |
| PR interval | ECG parameters | Cardiovascular |
| QRS duration | ECG parameters | Cardiovascular |
| QT interval | ECG parameters | Cardiovascular |
| QT interval (interaction) | ECG parameters | Cardiovascular |
| Resting heart rate | ECG parameters | Cardiovascular |
| RR interval (heart rate) | ECG parameters | Cardiovascular |
| Ventricular conduction | ECG parameters | Cardiovascular |
| Economic and political preferences | Economic and political preferences | NA |
| Economic and political preferences (environmentalism) | Economic and political preferences | NA |
| Economic and political preferences (fairness) | Economic and political preferences | NA |
| Economic and political preferences (feminism/equality) | Economic and political preferences | NA |
| Economic and political preferences (immigration/crime) | Economic and political preferences | NA |
| Economic and political preferences (time) | Economic and political preferences | NA |
| Epilepsy | Epilepsy | Nervous System |
| Epilepsy (generalized) | Epilepsy | Nervous System |
| Esophageal cancer | Esophageal cancer | Cancer |
| Esophageal cancer (alcohol interaction) | Esophageal cancer | Cancer |
| Esophageal cancer (squamous cell) | Esophageal cancer | Cancer |
| Esophageal cancer and gastric cancer | Esophageal cancer | Cancer |
| Exercise (leisure time) | Exercise | NA |
| Exercise treadmill test traits | Exercise | NA |
| Blue vs. brown eyes | Eye color | NA |
| Blue vs. green eyes | Eye color | NA |
| Eye color | Eye color | NA |
| Eye color traits | Eye color | NA |
| Iris characteristics | Eye color | NA |
| Iris color | Eye color | NA |
| Oleic acid (18:1n-9) plasma levels | Fatty acid plasma levels | Cardiovascular |
| Palmitic acid (16:0) plasma levels | Fatty acid plasma levels | Cardiovascular |
| Palmitoleic acid (16:1n-7) plasma levels | Fatty acid plasma levels | Cardiovascular |
| Stearic acid (18:0) plasma levels | Fatty acid plasma levels | Cardiovascular |
| Freckles | Freckles | NA |
| Freckling | Freckles | NA |
| Central corneal thickness | Glaucoma | NA |
| Vertical cup-disc ratio | Glaucoma | Eyes |
| Glioblastoma | Glioma | Cancer |
| Glioma | Glioma | Cancer |
| Glioma (high-grade) | Glioma | Cancer |
| Black vs. blond hair color | Hair color | NA |
| Black vs. red hair color | Hair color | NA |
| Blond vs. brown hair color | Hair color | NA |
| Hair color | Hair color | NA |
| Red vs non-red hair color | Hair color | NA |
| Red vs. non-red hair color | Hair color | NA |
| Hematocrit | Hematological parameters | Cardiovascular |
| Hematological parameters | Hematological parameters | Cardiovascular |
| Hematology traits | Hematological parameters | Cardiovascular |
| Hemoglobin | Hematological parameters | Cardiovascular |
| Hemostatic factors and hematological phenotypes | Hematological parameters | Cardiovascular |
| Mean corpuscular hemoglobin | Hematological parameters | Cardiovascular |
| Mean corpuscular hemoglobin concentration | Hematological parameters | Cardiovascular |
| Mean corpuscular volume | Hematological parameters | Cardiovascular |
| Other erythrocyte phenotypes | Hematological parameters | Cardiovascular |
| Red blood cell count | Hematological parameters | Cardiovascular |
| Red blood cell traits | Hematological parameters | Cardiovascular |
| Hepatitis B | Hepatitis B | Infectious disease |
| Hepatitis B (viral clearance) | Hepatitis B | Infectious disease |
| Hepatitis B vaccine response | Hepatitis B | Infectious disease |
| Chronic Hepatitis C infection | Hepatitis C | Infectious disease |
| Hepatitis C induced liver cirrhosis | Hepatitis C | Infectious disease |
| Hepatitis C induced liver fibrosis | Hepatitis C | Infectious disease |
| Lipid levels in hepatitis C treatment | Hepatitis C | Infectious disease |
| Response to hepatitis C treatment | Hepatitis C | Autoimmune |
| Hepatocellular carcinoma | Hepatocellular carcinoma | Infectious disease,Cancer |
| Hepatocellular carcinoma (hepatitis B virus related) | Hepatocellular carcinoma | Infectious disease,Cancer |
| Hippocampal atrophy | Hippocampal | Nervous System |
| Hippocampal volume | Hippocampal | Nervous System |
| AIDS | HIV | Infectious disease |
| AIDS progression | HIV | Infectious disease |
| HIV-1 control | HIV | Infectious disease |
| HIV-1 replication | HIV | Infectious disease |
| HIV-1 susceptibility | HIV | Infectious disease |
| HIV-1 viral setpoint | HIV | Infectious disease |
| HIV-associated dementia | HIV | Infectious disease |
| Hypertension | Hypertension | Cardiovascular |
| Hypertension (young onset) | Hypertension | Cardiovascular |
| Hypertension risk in short sleep duration | Hypertension | Cardiovascular |
| IgA levels | Ig Levels | Kidneys,Autoimmune |
| IgA nephropathy | Ig Levels | Kidneys,Autoimmune |
| IgE grass sensitization | Ig Levels | Autoimmune |
| IgE levels | Ig Levels | Autoimmune |
| IgG glycosylation | Ig Levels | Autoimmune |
| IgG levels | Ig Levels | Autoimmune,Infectious disease |
| IgM levels | Ig Levels | Autoimmune,Infectious disease |
| Immunoglobulin A | Ig Levels | Kidneys,Autoimmune |
| Immune reponse to smallpox (secreted IFN-alpha) | Immune response | Infectious disease |
| Immune reponse to smallpox (secreted IL-10) | Immune response | Infectious disease |
| Immune reponse to smallpox (secreted IL-12p40) | Immune response | Infectious disease |
| Immune reponse to smallpox (secreted IL-1beta) | Immune response | Infectious disease |
| Immune reponse to smallpox (secreted IL-2) | Immune response | Infectious disease |
| Immune reponse to smallpox (secreted TNF-alpha) | Immune response | Infectious disease |
| Immune response to anthrax vaccine | Immune response | Infectious disease |
| Immune response to smallpox vaccine (IL-6) | Immune response | Infectious disease |
| Inflammatory biomarkers | Immune response | Autoimmune |
| Interleukin-18 levels | Immune response | Autoimmune,Infectious disease |
| Crohns disease | Inflammatory bowel disease | Autoimmune |
| Crohns disease and celiac disease | Inflammatory bowel disease | Autoimmune |
| Crohns disease and psoriasis | Inflammatory bowel disease | Autoimmune |
| Crohns disease and sarcoidosis (combined) | Inflammatory bowel disease | Autoimmune |
| Inflammatory bowel disease | Inflammatory bowel disease | Autoimmune |
| Inflammatory bowel disease (early onset) | Inflammatory bowel disease | Autoimmune |
| Ulcerative colitis | Inflammatory bowel disease | Autoimmune |
| Intelligence | Intelligence | Nervous System |
| Intelligence (childhood) | Intelligence | Nervous System |
| Hepcidin levels | Iron | NA |
| Iron deficiency | Iron | NA |
| Iron levels | Iron | NA |
| Iron status biomarkers | Iron | NA |
| Kidney stones | Kidney stones | Kidneys |
| Nephrolithiasis | Kidney stones | Kidneys |
| Liver enzyme levels | Liver enzyme levels | NA |
| Liver enzyme levels (alanine transaminase) | Liver enzyme levels | NA |
| Liver enzyme levels (alkaline phosphatase) | Liver enzyme levels | NA |
| Liver enzyme levels (gamma-glutamyl transferase) | Liver enzyme levels | NA |
| Lung adenocarcinoma | Lung cancer | Cancer |
| Lung cancer | Lung cancer | Cancer |
| Lung Cancer (DNA repair capacity) | Lung cancer | Cancer |
| Lung cancer-asbestos exposure interaction | Lung cancer | Cancer |
| Lean body mass and age at menarche (combined) | Menarche | Body size |
| Monocyte count | Monocyte | NA |
| Monocyte early outgrowth colony forming units | Monocyte | NA |
| Multiple myeloma | Multiple myeloma | Cancer |
| Multiple myeloma (hyperdiploidy) | Multiple myeloma | Cancer |
| Multiple myeloma (IgH translocation) | Multiple myeloma | Cancer |
| Multiple sclerosis | Multiple sclerosis | Autoimmune |
| Multiple sclerosis (age of onset) | Multiple sclerosis | Autoimmune |
| Multiple sclerosis (OCB status) | Multiple sclerosis | Autoimmune |
| Multiple sclerosis (severity) | Multiple sclerosis | Autoimmune |
| Multiple sclerosis--Brain Glutamate Levels | Multiple sclerosis | Autoimmune |
| Myocardial infarction | Myocardial infarction | Cardiovascular |
| Myocardial infarction (early onset) | Myocardial infarction | Cardiovascular |
| Narcolepsy | Narcolepsy | Nervous System |
| Narcolepsy with cataplexy | Narcolepsy | Nervous System |
| Neuroblastoma | Neuroblastoma | Cancer |
| Neuroblastoma (high-risk) | Neuroblastoma | Cancer |
| Nonalcoholic fatty liver disease | Nonalcoholic fatty liver disease | NA |
| Non-alcoholic fatty liver disease | Nonalcoholic fatty liver disease | NA |
| Non-alcoholic fatty liver disease histology (AST) | Nonalcoholic fatty liver disease | NA |
| Non-alcoholic fatty liver disease histology (lobular) | Nonalcoholic fatty liver disease | NA |
| Non-alcoholic fatty liver disease histology (other) | Nonalcoholic fatty liver disease | NA |
| 2-Glycoprotein Iplasma levels | 2-Glycoprotein Iplasma levels | NA |
| 5-HTT brain serotonin transporter levels | 5-HTT brain serotonin transporter levels | Nervous System |
| AB1-42 | AB1-42 | Nervous System |
| Abdominal aortic aneurysm | Abdominal aortic aneurysm | Cardiovascular |
| Acenocoumarol maintenance dosage | Acenocoumarol maintenance dosage | Cardiovascular |
| Activated partial thromboplastin time | Activated partial thromboplastin time | Cardiovascular |
| Acute lymphoblastic leukemia (childhood) | Acute lymphoblastic leukemia (childhood) | Cancer |
| Addiction | Addiction | NA |
| Adiponectin levels | Adiponectin levels | NA |
| Adiposity | Adiposity | Body size |
| Adverse response to aromatase inhibitors | Adverse response to aromatase inhibitors | Cancer |
| Adverse response to carbamapezine | Adverse response to carbamapezine | Nervous System |
| Adverse response to lamotrigine and phenytoin | Adverse response to lamotrigine and phenytoin | Nervous System |
| Airflow obstruction | Airflow obstruction | NA |
| Allergic rhinitis | Allergic rhinitis | Autoimmune |
| Alopecia areata | Alopecia areata | Autoimmune |
| Amyloid A Levels | Amyloid A Levels | NA |
| Androgen levels | Androgen levels | NA |
| Angiotensin-converting enzyme activity | Angiotensin-converting enzyme activity | Cardiovascular |
| Ankle-brachial index | Ankle-brachial index | Cardiovascular |
| Ankylosing spondylitis | Ankylosing spondylitis | Autoimmune |
| Anorexia nervosa | Anorexia nervosa | Nervous System |
| Anthropometric traits | Anthropometric traits | Body size |
| Anticoagulant levels | Anticoagulant levels | Cardiovascular |
| Anti-cyclic Citrullinated Peptide Antibody | Anti-cyclic Citrullinated Peptide Antibody | Autoimmune |
| Antineutrophil cytoplasmic antibody-associated vasculitis | Antineutrophil cytoplasmic antibody-associated vasculitis | Autoimmune |
| Antipsychotic drug-induced weight gain | Antipsychotic drug-induced weight gain | NA |
| Apolipoprotein Levels | Apolipoprotein Levels | Cardiovascular |
| Arterial stiffness | Arterial stiffness | Cardiovascular |
| Arthritis (juvenile idiopathic) | Arthritis (juvenile idiopathic) | Autoimmune |
| Aspartate aminotransferase | Aspartate aminotransferase | NA |
| Asperger disorder | Asperger disorder | Nervous System |
| Aspirin exacerbated respiratory disease in asthmatics | Aspirin exacerbated respiratory disease in asthmatics | NA |
| Aspirin hydrolysis (plasma) | Aspirin hydrolysis (plasma) | NA |
| Atopic dermatitis | Atopic dermatitis | Autoimmune |
| Atopy | Atopy | Autoimmune |
| Autism | Autism | Nervous System |
| Autism spectrum disorder, attention deficit-hyperactivity disorder, bipolar disorder, major depressive disorder, and schizophrenia (combined) | Autism spectrum disorder, attention deficit-hyperactivity disorder, bipolar disorder, major depressive disorder, and schizophrenia (combined) | Nervous System |
| Barretts esophagus | Barretts esophagus | NA |
| Basal cell carcinoma | Basal cell carcinoma | Cancer |
| Behcets disease | Behcets disease | Autoimmune |
| Beta thalassemia/hemoglobin E disease | Beta thalassemia/hemoglobin E disease | NA |
| Beta-2 microglubulin plasma levels | Beta-2 microglubulin plasma levels | NA |
| Beta-trace protein levels | Beta-trace protein levels | NA |
| Biliary atresia | Biliary atresia | NA |
| Bilirubin levels | Bilirubin levels | NA |
| Biochemical measures | Biochemical measures | NA |
| Biomedical quantitative traits | Biomedical quantitative traits | NA |
| Birth weight | Birth weight | NA |
| Bitter taste response | Bitter taste response | NA |
| Bladder cancer | Bladder cancer | Cancer |
| Bleomycin sensitivity | Bleomycin sensitivity | NA |
| Blood pressure | Blood pressure | Cardiovascular |
| Brachial circumference | Brachial circumference | Body size |
| Brain connectivity | Brain connectivity | Nervous System |
| Brain imaging in schizophrenia (interaction) | Brain imaging in schizophrenia (interaction) | Nervous System |
| Brain lesion load | Brain lesion load | Nervous System |
| Brain structure | Brain structure | Nervous System |
| Breast size | Breast size | NA |
| Bulimia nervosa | Bulimia nervosa | Nervous System |
| Burning and freckling | Burning and freckling | NA |
| Butyrylcholinesterase levels | Butyrylcholinesterase levels | NA |
| C4b binding protein levels | C4b binding protein levels | Cardiovascular |
| Caffeine consumption | Caffeine consumption | NA |
| Calcium levels | Calcium levels | NA |
| Cannabis dependence | Cannabis dependence | NA |
| Cannbis use (initiation) | Cannbis use (initiation) | NA |
| Capecitabine sensitivity | Capecitabine sensitivity | NA |
| Cardiovascular disease risk factors | Cardiovascular disease risk factors | Cardiovascular |
| Carotenoid and tocopherol levels | Carotenoid and tocopherol levels | NA |
| Carotid atherosclerosis in HIV infection | Carotid atherosclerosis in HIV infection | Cardiovascular |
| Carotid intima media thickness | Carotid intima media thickness | Cardiovascular |
| Cataracts in type 2 diabetes | Cataracts in type 2 diabetes | Autoimmune |
| Caudate nucleus volume | Caudate nucleus volume | Nervous System |
| CD4:CD8 lymphocyte ratio | CD4:CD8 lymphocyte ratio | Autoimmune |
| Cervical cancer | Cervical cancer | Cancer |
| Chemerin levels | Chemerin levels | NA |
| Cholelithiasis-related traits in sickle cell anemia | Cholelithiasis-related traits in sickle cell anemia | NA |
| Chronic kidney disease | Chronic kidney disease | Kidneys |
| Chronic kidney disease and serum creatinine levels | Chronic kidney disease and serum creatinine levels | Kidneys |
| Chronic lymphocytic leukemia | Chronic lymphocytic leukemia | Cancer |
| Chronic myeloid leukemia | Chronic myeloid leukemia | Cancer |
| Circulating cell-free DNA | Circulating cell-free DNA | Autoimmune |
| Circulating vasoactive peptide levels | Circulating vasoactive peptide levels | Cardiovascular |
| Cleft lip | Cleft lip | NA |
| Coagulation factor levels | Coagulation factor levels | Cardiovascular |
| Coffee consumption | Coffee consumption | NA |
| Colorectal cancer | Colorectal cancer | Cancer |
| Common traits (Other) | Common traits (Other) | NA |
| Complement C3 and C4 levels | Complement C3 and C4 levels | Cardiovascular |
| Comprehensive strength and appendicular lean mass | Comprehensive strength and appendicular lean mass | Body size |
| Corneal astigmatism | Corneal astigmatism | Eyes |
| Corneal curvature | Corneal curvature | Eyes |
| Corneal structure | Corneal structure | Eyes |
| Cortical structure | Cortical structure | Eyes |
| Cortical thickness | Cortical thickness | Eyes |
| C-reactive protein | C-reactive protein | Autoimmune |
| C-reactive protein and white blood cell count | C-reactive protein and white blood cell count | Autoimmune |
| Creatinine levels | Creatinine levels | NA |
| Cutaneous nevi | Cutaneous nevi | NA |
| Cystatin C | Cystatin C | Kidneys |
| Cystic fibrosis severity | Cystic fibrosis severity | NA |
| Cytomegalovirus antibody response | Cytomegalovirus antibody response | Infectious disease |
| D-dimer levels | D-dimer levels | Cardiovascular |
| Dehydroepiandrosterone sulphate levels | Dehydroepiandrosterone sulphate levels | NA |
| Dengue shock syndrome | Dengue shock syndrome | Infectious disease |
| Dental caries | Dental caries | NA |
| Dialysis-related mortality | Dialysis-related mortality | NA |
| Diastolic blood pressure | Diastolic blood pressure | Cardiovascular |
| Dietary macronutrient intake | Dietary macronutrient intake | NA |
| Digit length ratio | Digit length ratio | NA |
| Dilated cardiomyopathy | Dilated cardiomyopathy | Cardiovascular |
| Disc degeneration (lumbar) | Disc degeneration (lumbar) | NA |
| Drinking behavior | Drinking behavior | NA |
| Duodenal ulcer | Duodenal ulcer | NA |
| Dupuytrens disease | Dupuytrens disease | NA |
| Echocardiographic traits | Echocardiographic traits | Cardiovascular |
| Electroencephalographic traits in alcoholism | Electroencephalographic traits in alcoholism | NA |
| Emphysema-related traits | Emphysema-related traits | NA |
| Endometrial cancer | Endometrial cancer | Cancer |
| Endometriosis | Endometriosis | NA |
| Endothelial function traits | Endothelial function traits | NA |
| End-stage coagulation | End-stage coagulation | Cardiovascular |
| End-stage renal disease | End-stage renal disease | Kidneys |
| End-stage renal disease (non-diabetic) | End-stage renal disease (non-diabetic) | Kidneys |
| Entorhinal cortical thickness | Entorhinal cortical thickness | Eyes |
| Eosinophil counts | Eosinophil counts | Autoimmune |
| Eosinophilic esophagitis (pediatric) | Eosinophilic esophagitis (pediatric) | Autoimmune |
| Epirubicin-induced leukopenia | Epirubicin-induced leukopenia | NA |
| Epstein-Barr virus immune response (EBNA-1) | Epstein-Barr virus immune response (EBNA-1) | Infectious disease |
| Erectile dysfunction | Erectile dysfunction | NA |
| Erectile dysfunction and prostate cancer treatment | Erectile dysfunction and prostate cancer treatment | NA |
| Erythrocyte sedimentation rate | Erythrocyte sedimentation rate | Autoimmune |
| E-selectin levels | E-selectin levels | NA |
| Essential tremor | Essential tremor | Nervous System |
| Estradiol levels | Estradiol levels | NA |
| Estradiol plasma levels (breast cancer) | Estradiol plasma levels (breast cancer) | NA |
| Event-related brain oscillations | Event-related brain oscillations | Nervous System |
| Ewing sarcoma | Ewing sarcoma | Cancer |
| Facial morphology | Facial morphology | NA |
| Factor VII | Factor VII | NA |
| Fat distribution (HIV) | Fat distribution (HIV) | NA |
| F-cell distribution | F-cell distribution | NA |
| Femoral neck bone geometry | Femoral neck bone geometry | NA |
| Fetal hemoglobin levels | Fetal hemoglobin levels | NA |
| Fibrinogen | Fibrinogen | Cardiovascular |
| Folate pathway vitamin levels | Folate pathway vitamin levels | NA |
| Follicular lymphoma | Follicular lymphoma | Cancer |
| Formal thought disorder in schizophrenia | Formal thought disorder in schizophrenia | Nervous System |
| Fuchss corneal dystrophy | Fuchss corneal dystrophy | Eyes |
| Functional MRI | Functional MRI | Nervous System |
| Gallbladder cancer | Gallbladder cancer | Cancer |
| Gallstones | Gallstones | NA |
| Gambling | Gambling | NA |
| Gamma gluatamyl transferase levels | Gamma gluatamyl transferase levels | NA |
| Gamma glutamyl transpeptidase | Gamma glutamyl transpeptidase | NA |
| Gastric cancer | Gastric cancer | Cancer |
| Gaucher disease severity | Gaucher disease severity | NA |
| Glaucoma | Glaucoma | Eyes |
| Glaucoma (exfoliation) | Glaucoma (exfoliation) | Eyes |
| Glaucoma (primary open-angle) | Glaucoma (primary open-angle) | Eyes |
| Glomerular filtration rate | Glomerular filtration rate | Kidneys |
| Glomerulosclerosis | Glomerulosclerosis | Kidneys |
| Gout | Gout | Autoimmune |
| Graves disease | Graves disease | Autoimmune |
| Hair morphology | Hair morphology | NA |
| Handedness in dyslexia | Handedness in dyslexia | NA |
| Haptoglobin levels | Haptoglobin levels | NA |
| HbA2 levels | HbA2 levels | NA |
| Head circumference (infant) | Head circumference (infant) | Body size |
| Hearing impairment | Hearing impairment | NA |
| Heart failure | Heart failure | Cardiovascular |
| Height | Height | Body size |
| Hip bone size | Hip bone size | Body size |
| Hip geometry | Hip geometry | Body size |
| Hirschsprungs disease | Hirschsprungs disease | NA |
| Hoarding | Hoarding | NA |
| Hodgkins lymphoma | Hodgkins lymphoma | Cancer |
| Homocysteine levels | Homocysteine levels | NA |
| HPV seropositivity | HPV seropositivity | Infectious disease |
| Hyperactive-impulsive symptoms | Hyperactive-impulsive symptoms | NA |
| Hypersomnia (HLA-DQB1*06:02 negative) | Hypersomnia (HLA-DQB1*06:02 negative) | Nervous System |
| Hypospadias | Hypospadias | NA |
| Hypothyroidism | Hypothyroidism | Autoimmune |
| Idiopathic pulmonary fibrosis | Idiopathic pulmonary fibrosis | NA |
| IFN-related cytopenia | IFN-related cytopenia | NA |
| Ileal carcinoids | Ileal carcinoids | Cancer |
| Inattentive symptoms | Inattentive symptoms | NA |
| Infantile hypertrophic pyloric stenosis | Infantile hypertrophic pyloric stenosis | NA |
| Information processing speed | Information processing speed | Nervous System |
| Insomnia (caffeine-induced) | Insomnia (caffeine-induced) | NA |
| Insulin-like growth factors | Insulin-like growth factors | Body size,Cardiovascular |
| Interstitial lung disease | Interstitial lung disease | NA |
| Intracranial aneurysm | Intracranial aneurysm | NA |
| Intracranial volume | Intracranial volume | NA |
| Intraocular pressure | Intraocular pressure | NA |
| Kawasaki disease | Kawasaki disease | Autoimmune |
| Keloid | Keloid | NA |
| Knee osteoarthritis | Knee osteoarthritis | NA |
| Large B-cell lymphoma | Large B-cell lymphoma | Cancer |
| Leishmaniasis (visceral) | Leishmaniasis (visceral) | Infectious disease |
| Lentiform nucleus volume | Lentiform nucleus volume | Nervous System |
| Leprosy | Leprosy | Infectious disease |
| Longevity | Longevity | NA |
| Lp (a) levels | Lp (a) levels | Cardiovascular |
| Lumiracoxib-related liver injury | Lumiracoxib-related liver injury | NA |
| Lymphocyte counts | Lymphocyte counts | Autoimmune,Infectious disease |
| Lymphoma | Lymphoma | Cancer |
| Magnesium levels | Magnesium levels | NA |
| Major CVD | Major CVD | Cardiovascular |
| Malaria | Malaria | Infectious disease |
| Male infertility | Male infertility | NA |
| Male-pattern baldness | Male-pattern baldness | NA |
| Mammographic density | Mammographic density | NA |
| Mathematical ability in children with dyslexia | Mathematical ability in children with dyslexia | NA |
| Matrix metalloproteinase levels | Matrix metalloproteinase levels | NA |
| Mean forced vital capacity from 2 exams | Mean forced vital capacity from 2 exams | NA |
| Melanoma | Melanoma | Cancer |
| Menarche (age at onset) | Menarche (age at onset) | NA |
| Menarche and menopause (age at onset) | Menarche and menopause (age at onset) | NA |
| Meningioma | Meningioma | Cancer |
| Meningococcal disease | Meningococcal disease | Infectious disease |
| Menopause (age at onset) | Menopause (age at onset) | NA |
| Metabolic traits | Metabolic traits | NA |
| Metabolite levels | Metabolite levels | NA |
| Methamphetamine dependence | Methamphetamine dependence | NA |
| Methotrexate clearance (acute lymphoblastic leukemia) | Methotrexate clearance (acute lymphoblastic leukemia) | NA |
| Migraine | Migraine | Nervous System |
| Monocyte chemoattractant protein-1 | Monocyte chemoattractant protein-1 | NA |
| Morbidity-free survival | Morbidity-free survival | NA |
| Mortality among heart failure patients | Mortality among heart failure patients | Cardiovascular |
| Moyamoya disease | Moyamoya disease | Cardiovascular |
| MRI atrophy measures | MRI atrophy measures | Nervous System |
| Multiple cancers (lung cancer, gastric cancer, and squamous cell carcinoma) | Multiple cancers (lung cancer, gastric cancer, and squamous cell carcinoma) | Cancer |
| Myasthenia gravis | Myasthenia gravis | Autoimmune |
| Myeloproliferative neoplasms | Myeloproliferative neoplasms | Cancer |
| Myopia (pathological) | Myopia (pathological) | Eyes |
| Nasopharyngeal carcinoma | Nasopharyngeal carcinoma | Cancer |
| Natriuretic peptide levels | Natriuretic peptide levels | Kidneys,Cardiovascular |
| Neonatal lupus | Neonatal lupus | Autoimmune |
| Nephropathy | Nephropathy | Kidneys |
| Nephropathy (idiopathic membranous) | Nephropathy (idiopathic membranous) | Kidneys |
| Nephrotic syndrome (acquired) | Nephrotic syndrome (acquired) | Kidneys |
| Neuranatomic and neurocognitive phenotypes | Neuranatomic and neurocognitive phenotypes | Nervous System |
| Neuroticism | Neuroticism | Nervous System |
| Neutrophil count | Neutrophil count | NA |
| Nevirapine-induced rash | Nevirapine-induced rash | NA |
| N-glycan levels | N-glycan levels | NA |
| Nicotine dependence | Nicotine dependence | NA |
| Nodular sclerosis Hodgkin lymphoma | Nodular sclerosis Hodgkin lymphoma | Cancer |
| Non-albumin protein levels | Non-albumin protein levels | NA |
| Non-melanoma skin cancer | Non-melanoma skin cancer | Cancer |
| Non-obstructive azoospermia | Non-obstructive azoospermia | NA |
| Non-small cell lung cancer | Non-small cell lung cancer | Cancer |
| Normalized brain volume | Normalized brain volume | Nervous System |
| Obsessive-compulsive disorder | Obsessive-compulsive disorder | Nervous System |
| Opioid sensitivity | Opioid sensitivity | Nervous System |
| Otosclerosis | Otosclerosis | NA |
| Ovarian cancer | Ovarian cancer | Cancer |
| Ovarian cancer in BRCA1 mutation carriers | Ovarian cancer in BRCA1 mutation carriers | Cancer |
| Ovarian reserve | Ovarian reserve | NA |
| Pagets disease | Pagets disease | NA |
| Pain | Pain | NA |
| Pancreatic cancer | Pancreatic cancer | Cancer |
| Pancreatitis | Pancreatitis | NA |
| Panic disorder | Panic disorder | Nervous System |
| Paraoxonase activity | Paraoxonase activity | NA |
| Partial epilepsies | Partial epilepsies | Nervous System |
| Pericardial fat | Pericardial fat | Cardiovascular |
| Periodontal microbiota | Periodontal microbiota | Infectious disease |
| Periodontitis | Periodontitis | Infectious disease |
| Peripheral artery disease | Peripheral artery disease | Cardiovascular |
| Personality dimensions | Personality dimensions | NA |
| Phospholipid levels (plasma) | Phospholipid levels (plasma) | Cardiovascular |
| Phosphorus levels | Phosphorus levels | NA |
| Phytosterol levels | Phytosterol levels | NA |
| Pit-and-Fissure caries | Pit-and-Fissure caries | NA |
| Plasminogen activator inhibitor type 1 levels (PAI-1) | Plasminogen activator inhibitor type 1 levels (PAI-1) | Cardiovascular |
| Polycystic ovary syndrome | Polycystic ovary syndrome | NA |
| Premature ovarian failure | Premature ovarian failure | NA |
| Presence of antiphospholipid antibodies | Presence of antiphospholipid antibodies | Autoimmune,Cardiovascular |
| Primary biliary cirrhosis | Primary biliary cirrhosis | Autoimmune |
| Primary sclerosing cholangitis | Primary sclerosing cholangitis | Autoimmune |
| Prion diseases | Prion diseases | Infectious disease |
| Progranulin levels | Progranulin levels | Nervous System |
| Progressive supranuclear palsy | Progressive supranuclear palsy | Nervous System |
| Protein biomarker | Protein biomarker | NA |
| Protein C levels | Protein C levels | Cardiovascular |
| Protein quantitative trait loci | Protein quantitative trait loci | NA |
| Prothrombin time | Prothrombin time | Cardiovascular |
| Psoriasis | Psoriasis | Autoimmune |
| Psoriatic arthritis | Psoriatic arthritis | Autoimmune |
| Psychosis (methamphetamine induced) | Psychosis (methamphetamine induced) | Nervous System |
| P-tau181p | P-tau181p | Nervous System |
| Pubertal anthropometrics | Pubertal anthropometrics | Body size |
| Pulmonary arterial hypertension (without BMPR2 mutations) | Pulmonary arterial hypertension (without BMPR2 mutations) | Cardiovascular |
| Quantitative traits | Quantitative traits | NA |
| Radiation response | Radiation response | NA |
| Reasoning | Reasoning | NA |
| Refractive error | Refractive error | NA |
| Renal cell carcinoma | Renal cell carcinoma | Cancer |
| Renal function and chronic kidney disease | Renal function and chronic kidney disease | Kidneys |
| Renal function-related traits (BUN) | Renal function-related traits (BUN) | Kidneys |
| Renal function-related traits (eGRFcrea) | Renal function-related traits (eGRFcrea) | Kidneys |
| Renal function-related traits (sCR) | Renal function-related traits (sCR) | Kidneys |
| Renal function-related traits (urea) | Renal function-related traits (urea) | Kidneys |
| Renal sinus fat | Renal sinus fat | Kidneys |
| Renal transplant outcome | Renal transplant outcome | Kidneys |
| Resistin levels | Resistin levels | NA |
| Response to acetaminophen (hepatotoxicity) | Response to acetaminophen (hepatotoxicity) | NA |
| Response to amphetamines | Response to amphetamines | NA |
| Response to angiotensin II receptor blocker therapy | Response to angiotensin II receptor blocker therapy | Cardiovascular |
| Response to angiotensin II receptor blocker therapy (opposite direction w/ diuretic therapy) | Response to angiotensin II receptor blocker therapy (opposite direction w/ diuretic therapy) | Cardiovascular |
| Response to antineoplastic agents | Response to antineoplastic agents | Cancer |
| Response to antipsychotic therapy (extrapyramidal side effects) | Response to antipsychotic therapy (extrapyramidal side effects) | Nervous System |
| Response to antipsychotic treatment | Response to antipsychotic treatment | Nervous System |
| Response to cerivastatin | Response to cerivastatin | Cardiovascular |
| Response to citalopram treatment | Response to citalopram treatment | Nervous System |
| Response to clopidogrel therapy | Response to clopidogrel therapy | Cardiovascular |
| Response to dabigatran etexilate treatment | Response to dabigatran etexilate treatment | Cardiovascular |
| Response to diuretic therapy | Response to diuretic therapy | Kidneys,Cardiovascular |
| Response to gemcitabine in pancreatic cancer | Response to gemcitabine in pancreatic cancer | Cancer |
| Response to iloperidone treatment (QT prolongation) | Response to iloperidone treatment (QT prolongation) | Cardiovascular |
| Response to interferon beta therapy | Response to interferon beta therapy | Autoimmune |
| Response to irinotecan and platinum-based chemotherapy in non-small-cell lung cancer | Response to irinotecan and platinum-based chemotherapy in non-small-cell lung cancer | Cancer |
| Response to irinotecan in non-small-cell lung cancer | Response to irinotecan in non-small-cell lung cancer | Cancer |
| Response to platinum-based agents | Response to platinum-based agents | Cancer |
| Response to platinum-based chemotherapy in non-small-cell lung cancer | Response to platinum-based chemotherapy in non-small-cell lung cancer | Cancer |
| Response to tamoxifen in breast cancer | Response to tamoxifen in breast cancer | Cancer |
| Response to taxane treatment (docetaxel) | Response to taxane treatment (docetaxel) | Cancer |
| Response to taxane treatment (placlitaxel) | Response to taxane treatment (placlitaxel) | Cancer |
| Response to temozolomide | Response to temozolomide | Cancer |
| Response to TNF antagonist treatment | Response to TNF antagonist treatment | Autoimmune |
| Response to TNF-alpha inhibitors in rheumatoid arthritis | Response to TNF-alpha inhibitors in rheumatoid arthritis | Autoimmune |
| Response to tocilizumab in rheumatoid arthritis | Response to tocilizumab in rheumatoid arthritis | Autoimmune |
| Response to treatment for acute lymphoblastic leukemia | Response to treatment for acute lymphoblastic leukemia | Cancer |
| Response to Vitamin E supplementation | Response to Vitamin E supplementation | NA |
| Restless legs syndrome | Restless legs syndrome | Nervous System |
| Retinal vascular caliber | Retinal vascular caliber | Cardiovascular |
| Retinol levels | Retinol levels | NA |
| Retinopathy in non-diabetics | Retinopathy in non-diabetics | Eyes |
| Rhegmatogenous retinal detachment | Rhegmatogenous retinal detachment | Eyes |
| Rheumatoid arthritis | Rheumatoid arthritis | Autoimmune |
| Ribavirin-induced anemia | Ribavirin-induced anemia | NA |
| Sagittal craniosynostosis | Sagittal craniosynostosis | NA |
| Sarcoidosis | Sarcoidosis | Autoimmune |
| Sclerosing cholangitis and ulcerative colitis (combined) | Sclerosing cholangitis and ulcerative colitis (combined) | Autoimmune |
| Scoliosis | Scoliosis | NA |
| Select biomarker traits | Select biomarker traits | NA |
| Self-rated health | Self-rated health | NA |
| Serum albumin level | Serum albumin level | NA |
| Serum ceruloplasmin levels | Serum ceruloplasmin levels | NA |
| Serum tamsulosin hydrochloride concentration | Serum tamsulosin hydrochloride concentration | NA |
| Serum total protein level | Serum total protein level | NA |
| Sex hormone-binding globulin levels | Sex hormone-binding globulin levels | NA |
| Sickle cell anemia (haemolysis) | Sickle cell anemia (haemolysis) | Cardiovascular |
| Skin pigmentation | Skin pigmentation | NA |
| Skin sensitivity to sun | Skin sensitivity to sun | NA |
| Small-cell lung cancer | Small-cell lung cancer | Cancer |
| Smooth-surface caries | Smooth-surface caries | NA |
| Soluble E-selectin levels | Soluble E-selectin levels | NA |
| Soluble ICAM-1 | Soluble ICAM-1 | NA |
| Soluble leptin receptor levels | Soluble leptin receptor levels | NA |
| Soluble levels of adhesion molecules | Soluble levels of adhesion molecules | NA |
| Speech perception in dyslexia | Speech perception in dyslexia | NA |
| Sphingolipid levels | Sphingolipid levels | NA |
| Spine bone size | Spine bone size | NA |
| Squamous cell carcinoma | Squamous cell carcinoma | Cancer |
| Stevens-Johnson syndrome and toxic epidermal necrolysis (SJS-TEN) | Stevens-Johnson syndrome and toxic epidermal necrolysis (SJS-TEN) | NA |
| Subclinical atherosclerosis traits (other) | Subclinical atherosclerosis traits (other) | Cardiovascular |
| Subcutaneous adipose tissue | Subcutaneous adipose tissue | NA |
| Substance dependence | Substance dependence | NA |
| Sudden cardiac arrest | Sudden cardiac arrest | Cardiovascular |
| Sunburns | Sunburns | NA |
| Systemic lupus erythematosus | Systemic lupus erythematosus | Autoimmune |
| Systemic sclerosis | Systemic sclerosis | Autoimmune |
| Systolic blood pressure | Systolic blood pressure | Cardiovascular |
| Tanning | Tanning | NA |
| Tardive dyskinesia | Tardive dyskinesia | Nervous System |
| Telomere length | Telomere length | NA |
| Testosterone levels | Testosterone levels | NA |
| Tetralogy of Fallot | Tetralogy of Fallot | NA |
| Thiazide-induced adverse metabolic effects in hypertensive patients | Thiazide-induced adverse metabolic effects in hypertensive patients | NA |
| Thoracic aortic aneurysms and dissections | Thoracic aortic aneurysms and dissections | Cardiovascular |
| Tonometry | Tonometry | NA |
| Tourette syndrome | Tourette syndrome | Nervous System |
| Treatment response for severe sepsis | Treatment response for severe sepsis | Infectious disease |
| T-tau | T-tau | Nervous System |
| Tuberculosis | Tuberculosis | Infectious disease |
| Tumor biomarkers | Tumor biomarkers | Cancer |
| Upper aerodigestive tract cancers | Upper aerodigestive tract cancers | Cancer |
| Urate levels | Urate levels | Kidneys |
| Uric acid levels | Uric acid levels | Kidneys |
| Urinary albumin excretion | Urinary albumin excretion | Kidneys |
| Urinary bladder cancer | Urinary bladder cancer | Cancer |
| Urinary metabolites | Urinary metabolites | Kidneys |
| Urinary symptoms in response to radiotherapy in prostate cancer | Urinary symptoms in response to radiotherapy in prostate cancer | NA |
| Uterine fibroids | Uterine fibroids | Cancer |
| Vaccine-related adverse events | Vaccine-related adverse events | Infectious disease |
| Vascular dementia | Vascular dementia | Cardiovascular |
| Vascular endothelial growth factor levels | Vascular endothelial growth factor levels | Cardiovascular |
| Vaspin levels | Vaspin levels | Cardiovascular |
| Vitamin B12 levels | Vitamin B12 levels | NA |
| Vitamin D insufficiency | Vitamin D insufficiency | NA |
| Vitamin D levels | Vitamin D levels | NA |
| Vitamin E levels | Vitamin E levels | NA |
| Vitiligo | Vitiligo | Autoimmune |
| Volumetric brain MRI | Volumetric brain MRI | Nervous System |
| vWF and FVIII levels | vWF and FVIII levels | Cardiovascular |
| Waist circumference | Waist circumference | Body size,Cardiovascular |
| Waist Circumference - Triglycerides (WC-TG) | Waist Circumference - Triglycerides (WC-TG) | Body size,Cardiovascular |
| Waist circumference and related phenotypes | Waist circumference and related phenotypes | Body size,Cardiovascular |
| Waist-hip ratio | Waist-hip ratio | Body size,Cardiovascular |
| Waist-to-hip circumference ratio (interaction) | Waist-to-hip circumference ratio (interaction) | Body size,Cardiovascular |
| Warfarin maintenance dose | Warfarin maintenance dose | Cardiovascular |
| Weight | Weight | Body size,Cardiovascular |
| Wilms tumor | Wilms tumor | Cancer |
| Working memory | Working memory | Nervous System |
| Wrist bone mass | Wrist bone mass | NA |
| YKL-40 levels | YKL-40 levels | NA |
| Obesity | Obesity | Body size,Cardiovascular |
| Obesity (early onset extreme) | Obesity | Body size,Cardiovascular |
| Obesity (extreme) | Obesity | Body size,Cardiovascular |
| Obesity and blood pressure | Obesity | Body size,Cardiovascular |
| Obesity and osteoporosis | Obesity | Body size,Cardiovascular |
| Obesity-related traits | Obesity | Body size,Cardiovascular |
| Optic disc parameters | Optic disc parameters | Eyes |
| Optic disc size (cup) | Optic disc parameters | Eyes |
| Optic disc size (disc) | Optic disc parameters | Eyes |
| Optic disc size (rim) | Optic disc parameters | Eyes |
| Orofacial clefts | Orofacial clefts | NA |
| Orofacial clefts (interaction) | Orofacial clefts | NA |
| Osteoarthritis | Osteoarthritis | NA |
| Osteonecrosis of the jaw | Osteoarthritis | NA |
| Osteoporosis | Osteoporosis | NA |
| Osteoporosis-related phenotypes | Osteoporosis | NA |
| Parkinsons disease | Parkinsons disease | Nervous System |
| Parkinsons disease (age of onset) | Parkinsons disease | Nervous System |
| Parkinsons disease (familial) | Parkinsons disease | Nervous System |
| Parkinsons disease (motor and cognition) | Parkinsons disease | Nervous System |
| Mean platelet volume | Platelets | NA |
| Platelet aggregation | Platelets | Cardiovascular |
| Platelet counts | Platelets | Cardiovascular |
| Platelet function and related traits | Platelets | Cardiovascular |
| Prostate cancer | Prostate cancer | Cancer |
| Prostate cancer (gene x gene interaction) | Prostate cancer | Cancer |
| Prostate-specific antigen levels | Prostate cancer | Cancer |
| Pulmonary function | Pulmonary function | NA |
| Pulmonary function (interaction) | Pulmonary function | NA |
| Pulmonary function decline | Pulmonary function | NA |
| Pulmonary function in asthmatics | Pulmonary function | NA |
| Recombination rate (females) | Recombination rate | NA |
| Recombination rate (males) | Recombination rate | NA |
| Schizophrenia | Schizophrenia | Nervous System |
| Schizophrenia (cytomegalovirus infection interaction) | Schizophrenia | Nervous System |
| Schizophrenia (negative symptoms) | Schizophrenia | Nervous System |
| Schizophrenia (treatment refractory) | Schizophrenia | Nervous System |
| Schizophrenia, bipolar disorder and depression (combined) | Schizophrenia | Nervous System |
| Sexual dysfunction (female) | Sexual dysfunction | NA |
| Sexual dysfunction (SSRI/SNRI-related) | Sexual dysfunction | NA |
| Sleep duration | Sleep | NA |
| Sleepiness | Sleep | NA |
| Smoking behavior | Smoking | NA |
| Smoking cessation | Smoking | NA |
| Stroke | Stroke | Cardiovascular |
| Stroke (ischemic) | Stroke | Cardiovascular |
| Stroke (pediatric) | Stroke | Cardiovascular |
| Suicidal ideation | Suicide | NA |
| Suicide attempts in bipolar disorder | Suicide | NA |
| Temperament | Temperament | Nervous System |
| Temperament (bipolar disorder) | Temperament | Nervous System |
| Testicular cancer | Testicular cancer | Cancer |
| Testicular germ cell cancer | Testicular cancer | Cancer |
| Testicular germ cell tumor | Testicular cancer | Cancer |
| Thyroid cancer | Thyroid | Cancer |
| Thyroid function | Thyroid | NA |
| Thyroid hormone levels | Thyroid | NA |
| Thyroid stimulating hormone | Thyroid | NA |
| Thyroid volume | Thyroid | NA |
| Thyrotoxic hypokalemic periodic paralysis | Thyroid | NA |
| Response to anti-TNF alpha therapy in inflammatory bowel disease | TNF treatment | Autoimmune |
| Response to anti-TNF treatment in rheumatoid arthritis | TNF treatment | Autoimmune |
| Permanent tooth development | Tooth development | NA |
| Primary tooth development (number of teeth) | Tooth development | NA |
| Primary tooth development (time to first tooth eruption) | Tooth development | NA |
| Venous thromboembolism | Venous thromboembolism | Cardiovascular |
| Venous thromboembolism (gene x gene interaction) | Venous thromboembolism | Cardiovascular |
| Visceral adipose tissue adjusted for BMI | Visceral adipose | Body size,Cardiovascular |
| Visceral adipose tissue/subcutaneous adipose tissue ratio | Visceral adipose | Body size,Cardiovascular |
| Visceral fat | Visceral adipose | Body size,Cardiovascular |
| White blood cell count | White blood cell | NA |
| White blood cell types | White blood cell | NA |
| White matter hyperintensity burden | White matter | Nervous System |
| White matter integrity | White matter | Nervous System |
| White matter integrity (interaction) | White matter | Nervous System |
