# Supplementary material for: CPAG: software for leveraging pleiotropy in GWAS to reveal similarity between human traits links plasma fatty acids and intestinal inflammation
Source: Genome Biol. 2015 Sep 15;16(1):190. doi: 10.1186/s13059-015-0722-1 (PMC4570686; doi:10.1186/s13059-015-0722-1)
Supplement: Additional file 6: Figure S4. — Relationship between the p value threshold for SNP inclusion and trait clustering. a Similar clusters for obesity (I), autoimmunity (II), and atherosclerosis (III) are observed with the different p value thresholds. Therefore, the detection of informative clusters is robust to varying the number of SNPs in the analysis, although traits are lost as the p value threshold is made more stringent. In all cases, the number of clusters (k) in the analysis was set to 20. The locations of the three clusters were also marked in the entire dendrogram and heat map of pairwise human traits for the p value threshold of 10−5 (b), 10−7 (c) and 10−10 (d). (ZIP 3188 kb) [file 13059_2015_722_add4b.pdf › 13059_2015_722_add4b.pdf]

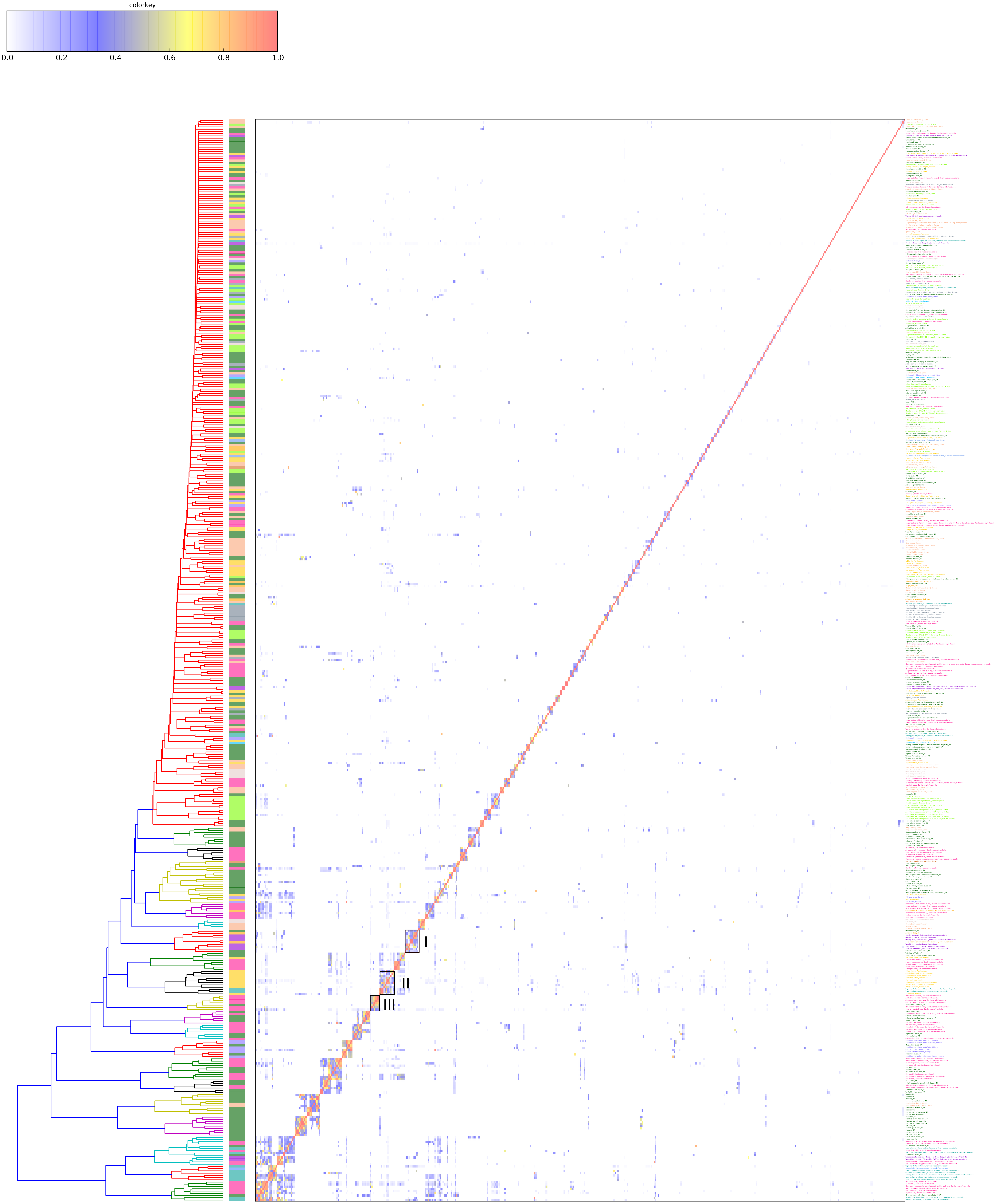
colorkey
0.0
0.2
0.4
0.6
0.8
1.0
I
II
III
